# Reprogramming homing endonuclease specificity through computational design and directed evolution

**DOI:** 10.1093/nar/gkt1212

**Published:** 2013-11-21

**Authors:** Summer B. Thyme, Sandrine J. S. Boissel, S. Arshiya Quadri, Tony Nolan, Dean A. Baker, Rachel U. Park, Lara Kusak, Justin Ashworth, David Baker

**Affiliations:** ^1^Department of Biochemistry, University of Washington, UW Box 357350, 1705 NE Pacific St., Seattle, WA 98195, USA, ^2^Graduate Program in Biomolecular Structure and Design, University of Washington, UW Box 357350, 1705 NE Pacific St., Seattle, WA 98195, USA, ^3^Graduate Program in Molecular and Cellular Biology, University of Washington, UW Box 357275, 1959 NE Pacific St., Seattle, WA 98195, USA, ^4^Department of Life Sciences, Sir Alexander Fleming Building, Imperial College London, Imperial College Road, London SW7 2AZ, UK, ^5^Department of Genetics, University of Cambridge, Downing Street, Cambridge CB1 3QA, UK, ^6^Institute for Systems Biology, 401 Terry Avenue N, Seattle, WA 98109, USA and ^7^Howard Hughes Medical Institute, University of Washington, UW Box 357350, 1705 NE Pacific St., Seattle, WA 98195, USA

## Abstract

Homing endonucleases (HEs) can be used to induce targeted genome modification to reduce the fitness of pathogen vectors such as the malaria-transmitting *Anopheles gambiae* and to correct deleterious mutations in genetic diseases. We describe the creation of an extensive set of HE variants with novel DNA cleavage specificities using an integrated experimental and computational approach. Using computational modeling and an improved selection strategy, which optimizes specificity in addition to activity, we engineered an endonuclease to cleave in a gene associated with *Anopheles* sterility and another to cleave near a mutation that causes pyruvate kinase deficiency. In the course of this work we observed unanticipated context-dependence between bases which will need to be mechanistically understood for reprogramming of specificity to succeed more generally.

## INTRODUCTION

Targeted gene correction requires site-specific DNA cleavage to initiate cellular programs for DNA break repair (1). A double-stranded break (DSB) can stimulate homologous recombination pathways to correct deleterious genetic mutations or insert a desired DNA sequence at the site of the break using a template with DNA arms that match the sequence surrounding the cleavage location. This process can be used to generate directed modifications in animal models and shows potential for human gene therapy (2). A number of nucleases—zinc-finger nucleases (ZFNs) (3), transcription activator-like effector nucleases (TALENs) (4), CRISPR nucleases (5,6) and homing endonucleases (HEs, also known as meganucleases) (7–9)—can make targeted breaks to activate gene conversion. Each of these reagents differs in its enzymatic properties and the ease of redesign of specificity.

**Figure 1.**
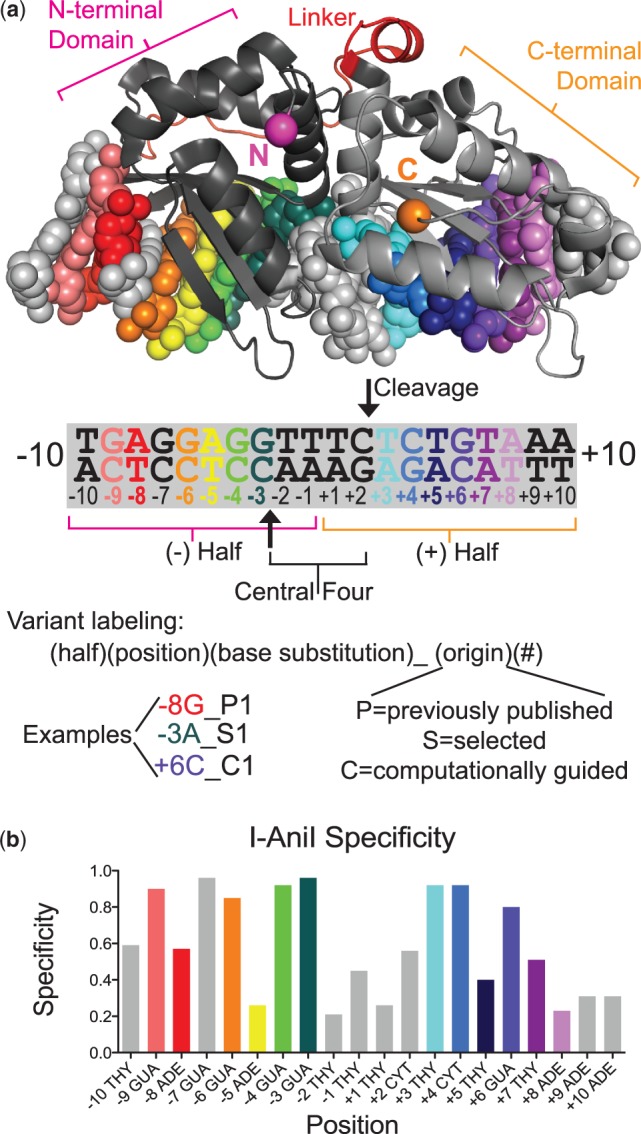
Structure and specificity of the I-AniI LAGLIDADG HE. (**a**) The I-AniI endonuclease (shown here, pdb code 2qoj) was used in this study, with the addition of activating mutations—Y2, M4 and M5, detailed in the methods—identified in previous work (32). Monomeric LAGLIDADG endonucleases are pseudo-symmetric, with two enzyme halves binding to the left (– half) and right (+ half) sides of the DNA target that flank the central four bases where cleavage occurs (arrow). The N-terminal domain binds to the (–) half-site and the C-terminal domain binds to the (+) half-site. The linker between the two regions is shown in red and the termini are marked with pink (N) and orange (C) spheres. The goal of our work is to alter the target site substrate preferences of these enzymes in order to direct their cleavage to genomic sites of interest. Many new variants cleaving single base-pair substitutions in the I-AniI target are presented in this work, and the labeling scheme for these variants is presented here. The ‘Selected’ enzymes were identified from fully randomized libraries, ‘Computationally guided’ enzymes were either improved versions of previous computational designs or selected from libraries containing computationally identified motif contacts, and the ‘Previously published’ enzymes are presented as well to show the full range of currently targeted positions in the I-AniI interface. (**b**) Experimentally determined specificity for the I-AniI endonuclease (Y2), derived from previously published kinetic data on each single base-pair substitution (12). Experimental specificity is defined in the Methods section on computational specificity prediction—a value close to 1.0 indicates that the enzyme has high specificity and a value of 0.25 indicates that all nucleotides are cleaved equally or that one other nucleotide is significantly preferred over the target nucleotide.

LAGLIDADG homing endonucleases (LHEs) (Figure 1a) have several desirable characteristics as gene therapy reagents (8). These enzymes are likely to cleave at few loci within a human genome because of their high specificity recognition (18–22 bp target sites, Figure 1b) and, because LHEs are typically single-chain proteins with coupled cleavage and binding activity (10,11), they have rapid cleavage kinetics (12) and a low potential for off-target cleavage compared to nucleases with less concerted activities. Other advantages of these nucleases include the 3' overhangs of the resultant DSB, potentially increasing rates of homologous recombination (13,14), and their small encoding ORFs without repetitive elements, desirable for nuclease delivery and maintenance in a host organism (15). The major disadvantage of these nucleases is that they are significantly more difficult to engineer to cleave novel target sites than some of their counterparts, such as the modular TALE nucleases (16). Overcoming this challenge is essential to using HEs in gene targeting applications.

Both experimental-directed evolution (17–19) and computational methods (20–24) have been developed for altering protein–DNA interaction specificity. The former approaches are limited by the number of protein positions that can be randomized and the need to maintain specificity while increasing activity on a desired target site; maintaining the naturally high specificity of these nucleases is critical if they are to be used as gene therapy reagents because off-target cleavage events can be detrimental (25). Computational design has the potential to resolve these issues by identifying a combination of amino acids that bind the desired target sequence with high affinity as well as specificity. However, while computational approaches have been used to design an enzyme cleaving multiple (three) adjacent base-pair substitutions (26), the success rate is still low (12,21,26,27), likely because features of endonuclease binding and catalysis are not yet well understood and therefore cannot be accurately modeled.

In this article, we present a combined experimental and computational method for reprogramming HE cleavage specificity. We approached the challenge of altering target site recognition by breaking it into two parts: first, the identification of many variants with specificity shifted by a single base-pair, and second, the utilization of this information to engineer nucleases cleaving more divergent target sites. New variants cleaving single base-pair substitutions were generated with an improved bacterial selection method (19), described in the first section of our report, which enables screening for cleavage specificity in addition to activity. We then engineered endonucleases cleaving target sites with multiple base-pair substitutions by integrating information from the single-site cleaving variants using computation and library selection. We generated endonucleases cutting full target sites in a gene for a human disease model and in a gene predicted to play a role in *Anopheles gambiae* reproduction (15,28).

## MATERIALS AND METHODS

### Construction of genes, libraries and target plasmids

The bacterial selection plasmids pENDO-HE and pCcdB for the original selection strategy were described previously (19) and were gifts from Barry Stoddard, originally from David Liu (Harvard University). The pET15-HE vector used for protein expression, a variant of pET15 that produces an N-terminal his-tag fusion protein using the NcoI and NotI restriction sites, was a gift from Barry Stoddard. The pCcdB plasmids containing the I-AniI LIB4 target sites (29) with single base-pair substitutions were built by ligating pCcdB vector cut with NheI and SacII to oligonucleotides (IDT, Integrated DNA Technologies) that were phosphorylated and annealed to form a duplex with compatible sticky ends. Two copies of each site were used for the selections against single base-pair substitutions. Some of the substrates (single copies of the site) used for enzyme assays were built the same way using the pCcdB vector, while others were constructed on pBluescript by site-directed mutagenesis (12,30). The tandem target site arrays were bought as minigenes from IDT and the arrays were inserted into the pCcdB plasmid with the NheI and SacII restriction sites. The negative selection vector was built by ligating two copies of wild-type (WT) I-AniI LIB4 target site into the NheI and SacII sites on the pENDO-HE vector. Amino acid libraries were built using assembly PCR (31) with oligonucleotides containing codons with degenerate nucleotides. These libraries were ligated into the modified pENDO-HE vector between the NcoI and NotI restriction sites. All C-terminal I-AniI libraries (starting at the interface position 150) were built in the context of the activating M5 mutations (F13Y, I55V, F91I, S92T, S111Y), and all N-terminal libraries (interface position 18 to position 72) were built in the context of M4, which is M5 without the I55V mutation (32) (Figure 1a). N-terminal variants were overexpressed with I-AniI-M4 mutations and C-terminal variants were overexpressed with I-AniI-Y2 mutations (F13Y, S111Y) because I-AniI-M5 expresses poorly (32).

### Directed evolution

A bacterial screen for variants of I-AniI that cleave single and multiple base-pair substitutions was carried out as previously described (19,33) with the improvements described in the results section. An electrocompetent strain of DH12S *Escherichia Coli* (Invitrogen) was transformed with a pCcdB plasmid. The pCcdB plasmids used contained either two adjacent copies of the activated LIB4 version (29) of the I-AniI target site with a single base-pair substitution or it contained a tandem array of 10–20 single copy target sites from genes of interest that contained various multiple base-pair substitutions. The number of cleavable target sites on the pCcdB plasmid is related to survival levels, and some endonucleases require as many as four WT sites to achieve near 100% survival (19). The optimized Y2 and M5 versions of I-AniI were previously identified by selection against four copies of the WT site (32), but show reasonable survival against two or even one (Supplementary Figure S1). A standard procedure for electrocompetent cell preparation was used to produce each pCcdB-containing strain.

For Round 1 selections, endonuclease libraries were ligated into the new negative selection pENDO-HE vector (containing two copies of the LIB4 WT I-AniI site), purified and transformed into 20 µl of the appropriate pCcdB-containing cell line. Ligation products were purified by running over a PCR cleanup column, leaving the wash buffer on the column for an extended period of time (5 min) to remove salts, and eluting the samples in 10 µl of water, all of which was transformed. Transformed bacteria were recovered in Terrific Broth (TB) media at 37°C for approximately one-half hour. The recovery was followed by a selection in 2 ml of liquid culture, containing 0.02% L-arabinose and 100 µg/ml carbenicillin, for 4 h at 30°C. After liquid selection, a volume of the culture was plated on a minimal media selection plate (100 µg/ml carbenicillin, 1 mM IPTG, 0.02% L-arabinose, 1.5% agar, M9 salt, 1% glycerol, 0.8% tryptone, 0.2% thiamine, 1 mM MgSO_4_, 1 mM CaCl_2_) and on a LB-agar, 100 µg/ml carbenicillin control plate. If the selection was a Round 1, the volumes plated were 1 µl on the control plate and the entire remainder of the selection (spun down and resuspended) on the selection plate. Plates are grown for ∼36 h at 30°C. For Round 2 selections, colonies were picked from the Round 1 selection plate, grown overnight, combined and miniprepped, and 1 µl of the mixed plasmid was re-transformed into 20 µl of the same competent cell line as the Round 1 ligation. The volumes plated on the Round 2 selection plates depended on the Round 1 survival and size of the starting library, but 0.2–1 µl was plated on the control plate and typically 0.2–1 µl and 200 µl on selection plates. Colonies on Round 2 selection plates were either grown up for a third round of selection or subjected to colony PCR to amplify the endonuclease ORF off of the pENDO-HE vector and the PCR products were sequenced.

### Protein production

Proteins were expressed and purified as previously described (34). The genes for each expressed I-AniI variant were either amplified from clones saved from the selection process or assembled *de novo* (31). These genes were cloned into pET15-HE with NcoI and NotI site, sequence verified and expressed in BL21 Star cells (Invitrogen) using autoinduction (35). Either a half- or full-liter of media was inoculated and grown at 37°C for 8–12 h. Expression at 18°C continued for 20–24 h, and then the cells were harvested, often frozen overnight, and resuspended in 20 mM Tris, pH 7.5, 30 mM Imidazole and 1.0 M NaCl. The cells were resuspended with lysozyme and sonicated, and proteins were isolated from the soluble fraction with nickel affinity chromatography and elution with 20 mM Tris, pH 7.5, 500 mM Imidazole and 500 mM NaCl. Proteins were concentrated and buffer exchanged into 20 mM Tris, pH 7.5, 500 mM NaCl using spin concentrators and stored in 50% (v/v) glycerol. The purity of the proteins assessed with SDS-PAGE and the mass was verified by mass spectrometry. The absorbance at 280 nm, collected with a NanoDrop, was used to determine the protein concentrations. Concentrations of proteins that were <90% pure were estimated from stained SDS-PAGE gels.

### *In vitro* endonuclease cleavage assays

Plasmid substrates were linearized, either with the restriction enzyme ScaI for targets on pBluescript (all sites with single base-pair substitution) or with XbaI for the pCcdB targets (multiple base-pair substitutions, full- and half-sites). Additional substrates, used in the assays shown in Figures 6a and 4, were generated by amplification of a 1 kb fragment from a pCcdB vector containing the sites. Experiments measuring kinetics always used the full-length plasmid substrates to maintain consistency with previously collected kinetic data (12). The enzyme reaction buffer was previously optimized for activity and stability of I-AniI, and contained final concentrations of 170 mM KCl, 10 mM MgCl_2_, 20 mM Tris, pH 9.0 and 1 mM DTT. To collect EC_1/2max_ data, corresponding to the half-maximal cleavage of the target site, endonuclease variants was assayed at several enzyme dilutions with ∼100 ng (5 nM) of linearized substrate for 30 min at 37°C. For every variant, eight serial 2-fold dilutions, ranging from 5 to 1500 nM of purified protein, were performed in 1.25X reaction buffer and then were diluted with substrate to 1X in a 10 µl volume. Reactions were halted with ∼17 nM EDTA, followed by 60°C incubation for 5–10 min. Cleavage products were separated on a 1.2% agarose TBE gel and stained with ethidium bromide. The spectral density of the substrate and product bands was quantified using ImageJ, as previously described. Fraction cleavage, calculated by dividing the total density of product and substrate bands by the product band densities, was plotted versus endonuclease concentration using GraphPad Prism. At least two independent assays were completed for each cleavage experiment. The EC_1/2max_ was calculated by fitting the fraction cleavage to a sigmoid function and using a value of 1.5 for the Hill coefficient, as previously described (34). Kinetics experiments were completed in the same way as these EC_1/2max_ experiments, and are previously described in further detail (12), except that the starting reaction volume was larger and samples were removed and the reaction halted at time points ranging from 15 s to 64 min. Reaction rates were calculated by plotting fraction cleavage versus time and the Michaelis–Menten parameters were calculated from relationship between reaction rate and enzyme concentration. For variants cleaving single base-pair substitutions, we defined specificity (36–39) as the following:

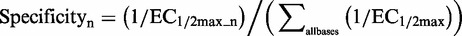


### Computational modeling for generating motif-biased libraries

All protocols were implemented within the Rosetta molecular modeling package (20). They are or will be available for free academic use through the Rosetta Commons, and are currently available to institutions participating in Rosetta Commons (or upon request). The code revision number used for the calculations in this work is 52 947.

Our strategy for using amino acid sidechain-base interactions frequently observed in the protein databank—interaction ‘motifs’—was introduced in (33). These motifs are defined as the spatial arrangement of six atoms, three on the DNA base and three on the protein residue interacting with that base. Protein–DNA motif pairs with high interaction energy, based on Rosetta calculations, were collected from previously published crystal structures and a library of motif interactions was generated. The information in these motifs was then used to bias the results of the computational modeling and design of libraries for experimental selection. In this work, amino acid conformations (rotamers) were built at protein positions in a 6 Å sphere surrounding the target base-pair and those conformations that were capable of forming a motif interaction from the motif library were identified. Starting from the backbone coordinates of the 2qoj protein–DNA complex, potential motif interactions with each base-pair substitution were identified using the Rosetta application motif_dna_packer_design. If a strong motif interaction could form between a particular amino acid type and the target base-pair, then the amino acid identity was fixed in the selected library for that base-pair. The exact tested libraries are available in Supplementary Tables S1 and S2.

### Target site selection for putative mosquito fertility genes

A position-specific scoring matrix (PSSM) matrix was applied to interrogate the *A. gambiae* genome and to produce a list of genes with potentially engineerable I-AniI target sites within their coding sequence, ranked in order of engineerability, to produce a subset of 500 genes. The code for the PSSM program that was used, written in C++ for rapid analysis of entire organism genomes, is available on github at the following address:

https://github.com/jashworth-isb/pssm–

From this subset of genes containing potential I-AniI sites, candidate genes with putative roles in female fertility were chosen based on either their tissue-specific expression profile as revealed from microarray data (28) or their annotated biology. Specifically, genes with high rank-normalized ovary expression and high tissue restriction (*tau*) (28) of this expression were prioritized, as were genes whose direct ortholog in the model insect *Drosophila melanogaster* were known to have at least one female sterile allele. From a list of genes that were considered to have suitable biology to warrant targeting by a re-engineered I-AniI, the genes containing the sites shown in Table 1 were chosen as targets based on the predicted ease of engineering an I-AniI variant to cleave the site.

**Table 1.** Target sites were identified in genes associated with *Anopheles* sterility and genetic diseases

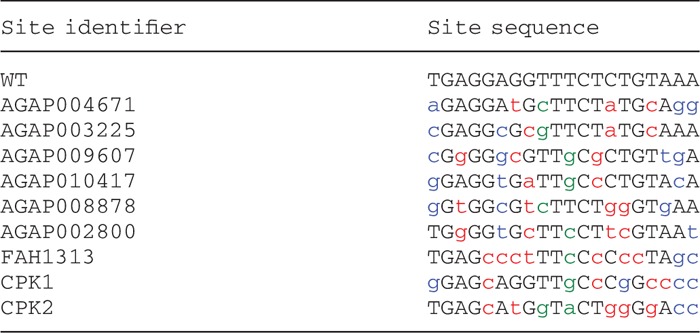

The WT I-AniI target sequence is shown in all upper-case letters, whereas substitutions in these target sites are in lower-case. Substitutions colored blue are tolerated by the WT I-AniI endonuclease as single changes and are not directly adjacent to a position that is not tolerated. Substitutions that are not tolerated and are the targets of interface engineering are colored red. Central four substitutions are colored green. Sites labeled with the AGAP prefix are in the *A. gambiae* genome, the FAH1313 site is in a murine model of tyrosinemia, and the CPK sites are in a canine model of PK deficiency.

## RESULTS

### Improving bacterial selection methods for achieving new cleavage specificities

**Figure 2.**
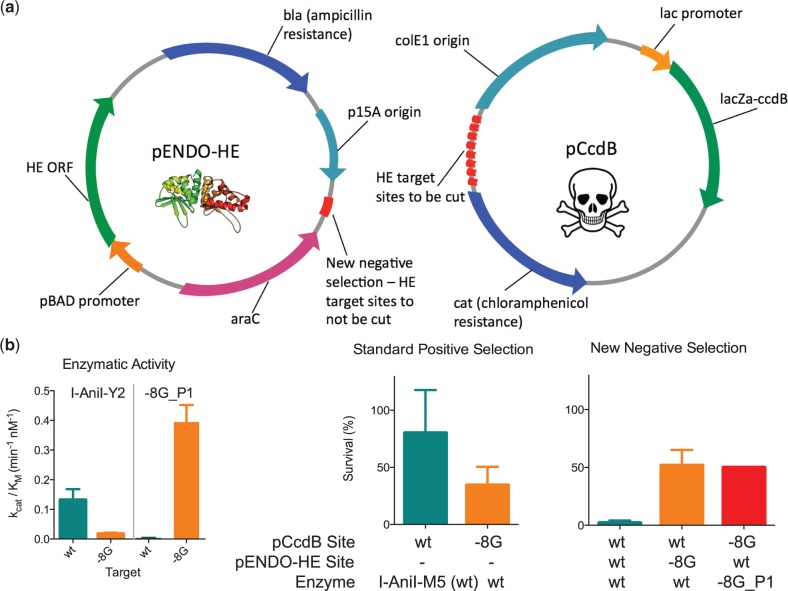
Bacterial directed evolution system for selecting endonuclease variants with high specificity. (**a**) Schematics of the plasmids used in the bacterial selection system. The pENDO-HE plasmid expresses the endonuclease and contains an HE target for negative selection. The pCcdB plasmid expresses the CcdB protein, a DNA gyrase poison, which causes bacterial cell death if the target sites on the plasmid are not cleaved by the endonuclease. Both the modifications to allow for specificity selection and increase system throughput are shown in red. (**b**) Comparisons of endonuclease activity with kinetic data from *in vitro* cleavage assays, survival in the original selection system and survival in the improved system with the specificity selection component. An extended comparison of survival and *in vitro* cleavage activity for multiple single base-pair substitutions in the I-AniI target is available in Supplementary Figure S1.

Bacterial selection can be a powerful tool for reprogramming protein–DNA interactions and screening computationally designed libraries. A previously described selection system couples survival of bacteria to cleavage of a plasmid containing both the target site of interest and an ORF coding for a toxic DNA gyrase poison (CcdB) (19). Target site cleavage causes degradation of the pCcdB plasmid, allowing for survival and retrieval of active variants (Figure 2a). The two drawbacks to this system are that it can favor non-specific enzymes and is quite labor-intensive. Non-specific enzymes are generated because the selective pressure of the system is for enzyme activity rather than specificity. The selection is low-throughput both because of high background levels of survival and because a different pCcdB-containing strain of bacteria must be prepared for every target site of interest. Even a background survival as low as 0.1% translates into significant secondary screening efforts for library sizes of 10^5^ or greater, as there would be on the order of 100 background colonies. To overcome these difficulties, we introduced an additional selective pressure for specificity that reduces background survival, and increased system throughput by including several target sites (>10) in tandem onto a single pCcdB plasmid, allowing a single bacterial strain to be used to test multiple targets.

To select for enzyme specificity in addition to activity, a target DNA sequence (typically the native endonuclease site) for which cleavage is not desired is placed on the plasmid expressing the endonuclease protein (pENDO-HE) so that its cleavage results in loss of the HE ORF and hence cell death due to CcdB expression. Bacterial survival is thus linked to cleavage of the desired site on the toxic pCcdB as well as lack of cleavage of the undesired site on the nuclease encoding pENDO-HE (Figure 2b). As expected, target sites that are cleaved well in *in vitro* cleavage assays resulted in low bacterial survival when included on the pENDO-HE plasmid, while positions that are cleaved with low efficiency displayed high survival (Supplementary Figure S1). Enzymes with high specificity for their intended target show high survival in a system with the WT site on pENDO-HE (Figure 2b), indicating that the new specificity selection works as designed. The addition of specificity selection also improved system throughput by eliminating background survival of low-specificity and high-activity WT enzymes, thus reducing the screening efforts necessary to identify variants with successful specificity switches. Further increasing system throughput, we alleviated the need for making a pCcdB-containing bacterial strain for every target site being tested by putting many divergent target sites, differing by more than a single base-pair not tolerated by the WT endonuclease, on one pCcdB plasmid (see Supplementary Methods). The improved system was used to engineer enzymes for multiple base-pair substitutions and full target sites in genes of interest.

### Generating new HE specificities by integrating computation and experimental selection

**Figure 3.**
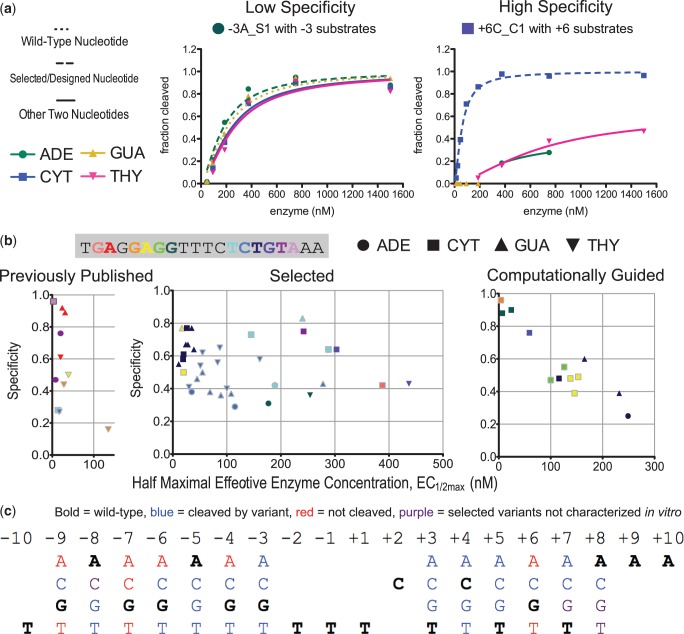
Combining directed evolution with computational design to successfully select for variants cleaving targets with single base-pair substitutions in the I-AniI target site. (**a**) Representative data from cleavage assays with each of the four single base-pair substitutions at the targeted position for variants exhibiting high or low specificity. Data for the other I-AniI variants are available in Supplementary Figure S2. (**b**) Activity and specificity of I-AniI endonuclease variants that cleave single base-pair substitutions (Supplementary Figure S2, Supplementary Table S1). Each point in the graphs is an enzyme variant and is colored to reflect the substituted nucleotide in the cleaved DNA target, both those toward which the enzyme was evolved or the preferred substitution, in the case where an alternate non-WT nucleotide was preferred with over twice the specificity to the target. These three plots include the majority of the 64 variants discussed in this work, with the exceptions of two published enzymes without EC_1/2max_ data available and three selected enzymes with EC_1/2max_-values of over 500 nM for all base-pairs at the target position. (**c**) Summary of the single base-pair substitutions in the I-AniI target site that can be cleaved with the engineered variants. Of the 39 substitutions for which selection was attempted—substitutions at −10, +9, +10, and the central four were skipped—variants cleaving 26 substitutions were expressed and characterized (Supplementary Table S1). Blue, sites cleaved by engineered variants; purple, sites for which high-surviving variants were identified but the endonucleases were not expressed; and red, sites for which cleaving variants were not obtained.

The bacterial selection system with the negative selection specificity modification was used to generate highly specific variants of the LAGLIDADG endonuclease I-AniI that cleave sites with single base-pair substitutions in the WT target (Supplementary Table S1, Supplementary Figure S2). Initially, libraries were made by randomizing several protein residues neighboring base-pairs for which no successful computational designs were previously recovered. In addition to generating variants using randomized libraries, several previously described I-AniI computational designs (12) with high specificity but low overall activity were improved using the modified selection system; the residues predicted to be most critical to the switch in specificity were fixed and the surrounding residues varied. Many of these selected endonucleases were expressed and purified, and their specificity was measured by *in vitro* cleavage assays with each of the four single base-pair substitutions at the targeted position (Figure 3a). Enzymes obtained from these experiments with high specificities often contain residues predicted to form strong hydrogen bonds with the target base-pair (Supplementary Figure S3).

To favor redesigns with such strong sidechain-base hydrogen bonds, similar to the improved computational designs, we used a computational procedure to search for canonical protein–DNA interactions, which we refer to as ‘motifs’, extracted from a large set of protein–DNA structures (33,40). The motifs are collected from high-resolution crystal structures based on calculated interaction energies between sidechain and base atoms. To incorporate these motif interactions in interface designs, sidechain conformations (rotamers) for all 20 amino acids are generated for protein positions near a substituted base and the rotamers are scored based on whether they can form one of the interactions seen in the motif library. We showed previously that inclusion of the rotamers making motif interactions improved results for computational benchmarks (33), but the methods were not evaluated with experimental testing.

For each substitution in the I-AniI target site, libraries were generated where an amino acid type computationally predicted to make a motif interaction was kept fixed and surrounding residues were varied. These libraries were built both for single base-pair substitutions for which variants had already been identified with the fully randomized approach, and also for positions were no variant had yet been identified. Endonuclease variants with new specificities were selected from these motif-derived libraries, including some for target site substitutions for which active nuclease variants were not previously recovered. The likelihood of identifying an active and specific variant is increased in these computational motif libraries because all members contain a predicted high-quality contact and because the library size is reduced (41). For example, libraries with full randomization of positions 18, 33, 59 and 68 yielded no high-surviving variants for position −4C, but a library with a motif lysine fixed at position 33 (and randomization of the other three positions, as well as position 31) showed 46% survival after two rounds of selection (Supplementary Table S1).

The combination of the selected variants described and those previously published (12,34) allow targeting of a large number of single base-pair substitutions in the I-AniI target site (Figure 3b, Supplementary Figure S2 and Supplementary Table S1). There are 60 possible single base-pair substitutions in the 20 bp I-AniI site (20 × 3). In this work, we attempted to generate variants cleaving 39 of the 60 substitutions, excluding those under the two edge loops of the endonuclease (–10, +9 and +10, relatively non-specific regions of the interface (12)) and those in the central four bases that are not directly contacted by amino acids. Both of these areas are challenging to engineer and model-loops are flexible and the sequence preference in the central four is not currently understood (12,34,42,43). In total, we characterized 64 I-AniI variants cleaving 26 distinct single base-pair substitutions (12,34) out of the 39 attempted, 51 of which were generated in this work (Figure 3c, Supplementary Table S1; we obtained libraries cleaving four additional sites but did not characterize individual clones). Libraries were attempted and no reasonably active variants were identified for −9A, −9T, −6A, −4A, +6A, +6T and all three substitutions at position −7. This extensive dataset of single base-pair specificity switches is a valuable benchmark that can be used for evaluating and guiding the improvement of computational design methods. We describe preliminary results comparing computational predictions and experimental data in the Supplementary Material (Supplementary Figures S4, S5 and S6, Supplementary Discussion A).

### Combining single base-pair variants to cleave target half-sites

We chose *Anopheles* reproduction (15) and several genetic disorders as model challenges for genome engineering with endonucleases. To identify near-native I-AniI target sites in the relevant genes, we used a PSSM (26), provided in the Supplementary Methods, that incorporates information about substitutions that can be cleaved by the WT endonuclease (Figure 1b) and those that are cleaved by the newly selected variants (Figure 3c). Including specificity data for the large number of new I-AniI variants evolved in this work was critical to identifying target sites requiring minimal enzyme engineering. Potential target sites for I-AniI engineering were found in the genes involved in the diseases pyruvate kinase (PK) deficiency (44) and tyrosinemia (45), as well as several genes putatively associated with *Anopheles* reproduction (Table 1) (28). Targets from genetic diseases in which the desired outcome is to correct mutations using homologous recombination were chosen to be very close to the site of mutation in order to ensure efficient gene conversion. We chose a site in the PK gene—CPK2 (canine PK site 2)—occurring only 53 bases away from the mutation found in the canine model of this disease (44).

Redesign to cleave target sites of interest will almost always involve changing specificity at multiple base-pairs. In early experiments, we found that mutations from two single base-pair variants could be successfully combined without further selection, even when they were separated by only a single base-pair (Supplementary Figure S7). However, this approach is unlikely to work for base substitutions directly neighboring one another as mutating amino acids to alter the specificity at one position could result in changes in specificity at the neighboring bases. Also, if multiple variants have been identified for a particular single base-pair substitution, utilizing only one such variant does not take advantage of the sequences of others that may prove more compatible with other desired target site changes. To address these challenges, we combined information from the already identified I-AniI single base-pair variants with computational modeling, assessing the compatibility of the amino acid mutations present in the single-site cleaving variants and identifying new motif residues for adjacent substitutions, and performed further *in vivo* selection. We broke down the target sites into groups of substitutions that were close together or into half-sites, building separate libraries for groups of substitutions in the two half-sites.

**Figure 4.**
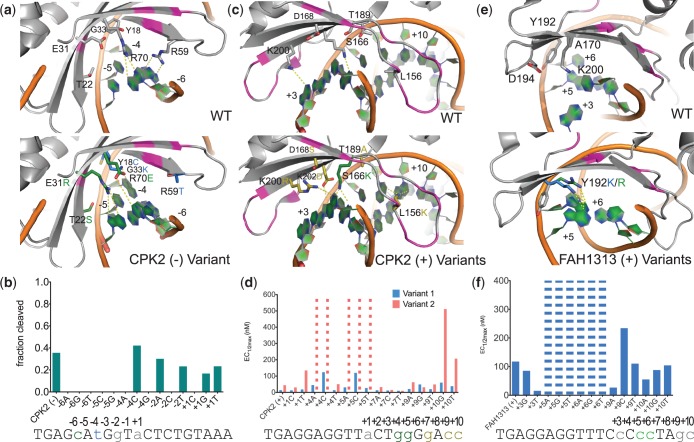
Combining computation and selection to cleave multiple base-pair substitutions with high specificity. Libraries screened to generate variants, exact mutations tested and the data shown are detailed in Supplementary Table S2 and Supplementary Figure S8. The WT amino acids in the region of interest are shown in gray in the upper portion of panels (**a**, **c** and **e**), and residues predicted to be important for activity and specificity (data in panels (**b**, **d** and **f**)), are shown in the lower portion. Protein backbone positions shown as magenta are supporting positions that were varied in the selection process, but did not yield the dominant contacts. Any substrates not shown in panels (b, d and f) are missing because the process used to generate the target sites utilized a batch method for large numbers of substrates mixed together and these missing constructs were not successfully derived from the group. Bars with dashes indicate that no cleavage was visible at the highest tested enzyme concentration. (a) The (–) half of the CPK2 site contains multiple base-pair substitutions, including −6C and −4T. An enzyme was engineered to target a site with both changes by combining amino acid mutations from variants previously engineered for each single base-pair substitution. Mutations targeting −6C (from −6C_C1) are shown in green and mutations for −4T (from −4C_C1) are shown in blue. (b) Cleavage of the CPK (–) half-site with the CPK2_N enzyme (exact mutations in Supplementary Table S2) and targets containing the indicated base substitution, measured by *in vitro* cleavage with ∼1700 nM of the CPK2_N enzyme. (c) The (+) half of the CPK2 site contains the adjacent substitutions +4G and +5G, as well as the substitutions +7G, +9C and +10C. The +4G/+5G pocket was targeted before the entire half-site by selecting enzymes from motif-biased libraries, and a library containing the S166K (green) motif produced successful variants that provided a starting place for further selections against the entire (+) half-site. Positions in yellow are contacts that were identified from selections against the CPK2 (+) half-site. For example, the mutation L156K is predicted to hydrogen bond with the two guanines on the reverse strand of the +9C/+10C double base-pair pocket and the D168S mutation caused a significant increase in survival over a D168A-containing variant (Supplementary Table S2). (d) Activity data for two enzyme variants, CPK2_C1 (variant 2) and CPK2_C3 (variant 1), selected from libraries based on the positions shown in panel (c) tested *in vitro* against the CPK2 (+) half-site and targets with the indicated single base-pair substitution. The two variants differ from each other by two mutations, D168S and T204A, in CPK2_C3 (variant 1) that increase activity over CPK2_C1 (variant 2). (e) The FAH1313 (+) half-site contains the adjacent substitutions +5C and +6C. A library incorporating a motif residue at position 192 was targeted to this double base-pair pocket. Both a lysine and an arginine residue were identified by the computational methods, and surviving variants included both mutations. (f) Activity data for the enzyme FAH1313_Ccc (Table S2), selected to cleave +5C/+6C and incorporating the Y192K motif residue (panel (e)). Data were collected for the full FAH1313 site and all single base-pair substitutions.

We used this approach to engineer variants cleaving the half-sites of a target site identified in the CPK2 gene (Figure 4). For the (–) half of the CPK2 site (Figure 4a), a library was generated incorporating mutations from two single base-pair variants cleaving −6C and −4T with randomization allowed in the region in between and surrounding the two positions (Supplementary Table S2). The mutations identified by screening of this library using the bacterial selection system resulted in a variant with increased specificity at position-5 (compared to the WT I-AniI that cleaves all 4 nt equally (Figure 1b)) (Figure 4b). The specificity at −6 and −4 was similar to that of the starting single base-pair variants (Supplementary Figure S2, Supplementary Table S1), and the WT specificity was maintained at positions −2 and +1 (Figure 1b). Modeling the sequence changes of amino acids arising from single base-pair variants ensured their compatibility and identified neighboring protein positions that were varied in the subsequent selection libraries. The (+) half of the CPK2 site contains multiple substitutions, including two adjacent substitutions, +4G and +5G, and a third, +7G, separated from the +4G/+5G base-pair pocket by only a single position. The close proximity of these changes made it impossible to directly combine already identified variants cleaving single base-pair substitutions. Therefore, candidate motifs compatible with the group of substitutions were identified computationally and incorporated into a library with surrounding randomization, which was selected against a target with the +4G/+5G substitutions alone. From this selection, variants with the S166K motif incorporated (Figure 4c) were found to have high survival (Supplementary Table S2). Sequences of active endonucleases from these S166K selections were used to generate further libraries that incorporated randomization over the +7G nt. The evolved variant chosen from this selection cleaved +7G well, whereas the WT enzyme did not (Figure 1b). While this endonuclease also tolerated other substitutions at +7, further optimization of protein positions near +9 and +10 resulted in greatly increased specificity at position +10 compared to the WT enzyme (Figure 1b) and high activity on the CPK2 (+) half-site (Figure 4d).

While this step-wise approach proved successful for engineering variants cleaving both halves of the CPK2 site, we had less success for a target in the FAH1313 gene (Table 1). We were able to generate a highly active and specific variant for two substitutions, +5C and +6C, by screening a library containing two *in silico* predicted motifs, Y192K and Y192R (Figure 4e and f, Supplementary Table S2). However, when these mutations were combined with those from several variants available that cleave +3C well (Figure 3, Supplementary Figure S2, Supplementary Table S1; positions 170, 194 and 200), none of the libraries tested yielded endonucleases with high specificity and activity for the entire (+) half-site. This result suggests that there may be unanticipated coupling between regions of the endonuclease interface.

### Characterizing unanticipated specificity shifts in endonuclease catalysis

**Figure 5.**
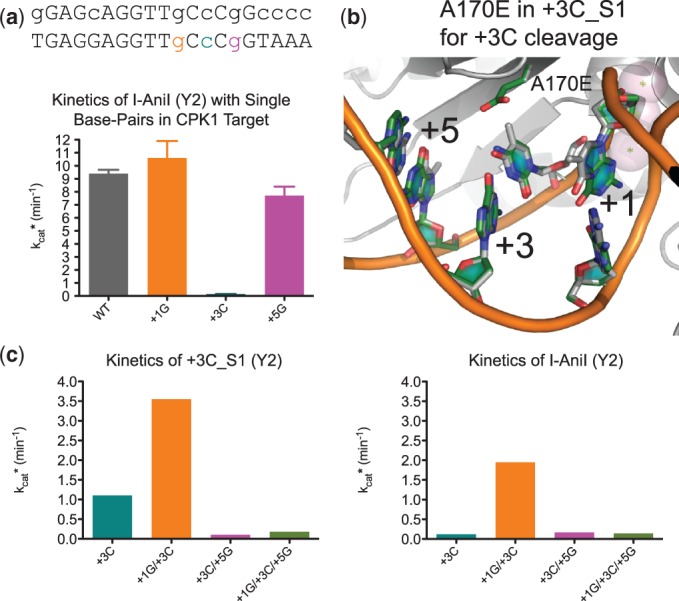
Combining (+) side target substitutions results in cleavage specificity switches that are independent of protein sequence. (**a**) Kinetic data (*k*_cat_*) for the I-AniI enzyme with three targets containing single base-pair substitutions found in the CPK1 site. The I-AniI enzyme cleaves two of three substitutions, +1G and +5G, as efficiently as the WT target and does not cleave the third, +3C. (**b**) Variants selected to cleave the +3C position contained the A170E mutation over the cytosine. This amino acid is predicted to form a motif interaction with the cytosine nucleotide. (**c**) Kinetic parameters for the +3C_S1 (A170E, D194K, K200A and Y2 mutations) and I-AniI-Y2 enzymes against targets with different combinations of +1G and +5G with the +3C substitution.

A particularly striking case of site coupling was observed in the course of targeting a second site in the canine PK gene, CPK1 (Table 1). This site contains the substitutions +1G, +3C and +5G, as well as additional substitutions farther away from the central four bases. Both +1G and +5G are cleaved as well as the WT nucleotides by I-AniI, but the +3C substitution is cleaved very poorly (Figure 5a) (12). We succeeded in generating several enzyme variants with high specificity and activity for +3C using the bacterial selection. The most specific of these, +3C_S1, contains the mutation A170E, predicted to form a direct contact with the +3 cytosine nucleotide (Figure 5b). This enzyme cleaves a target containing both the +1G and +3C substitutions more rapidly (higher *k*_cat_*) than the +3C target alone. Surprisingly, however, the triply substituted site +1G/+3C/+5G is cleaved very slowly (low *k*_cat_*). To determine whether this unexpected coupling is specific to the evolved variant, we measured the cleavage rates of the WT I-AniI endonuclease (Y2 version) on the same set of target substrates (+1G/+3C, +3C/+5G and +1G/+3C/+5G). The overall pattern of *k*_cat_*-values was very similar between the two enzymes (Figure 5c), suggesting there are substrate preferences that are largely independent of the protein sequence, further complicating the computational design process. Indirect readout and target preferences inherent to the DNA substrate have been previously reported for this family of endonucleases in the central four region (43), and the current results show that this context-dependence extends beyond the central four.

### Targeting full cleavage sites in mosquito and disease genes

We also identified several target sites associated with *Anopheles* sterility (Table 1). These targets contain fewer position changes than the CPK2 target, and a number of these changes can be cleaved individually by the new variants generated in this work (Figure 3c). Furthermore, through selection against the AGAP003225 (+) half-site combining the +7C_S1 variant with sequence information from variants targeting the +4A base-pair (Supplementary Figure S2, Supplementary Table S1), we generated a variant cleaving a site with two single base-pair substitutions present in both the AGAP004671 and AGAP003225 (+) half-sites. This variant cleaves the AGAP003225 (+) half-site with an *in vitro* EC_1/2max_ of 22 nM and shows activity in human cell assays (Supplementary Figure S9) (42,46).

**Figure 6.**
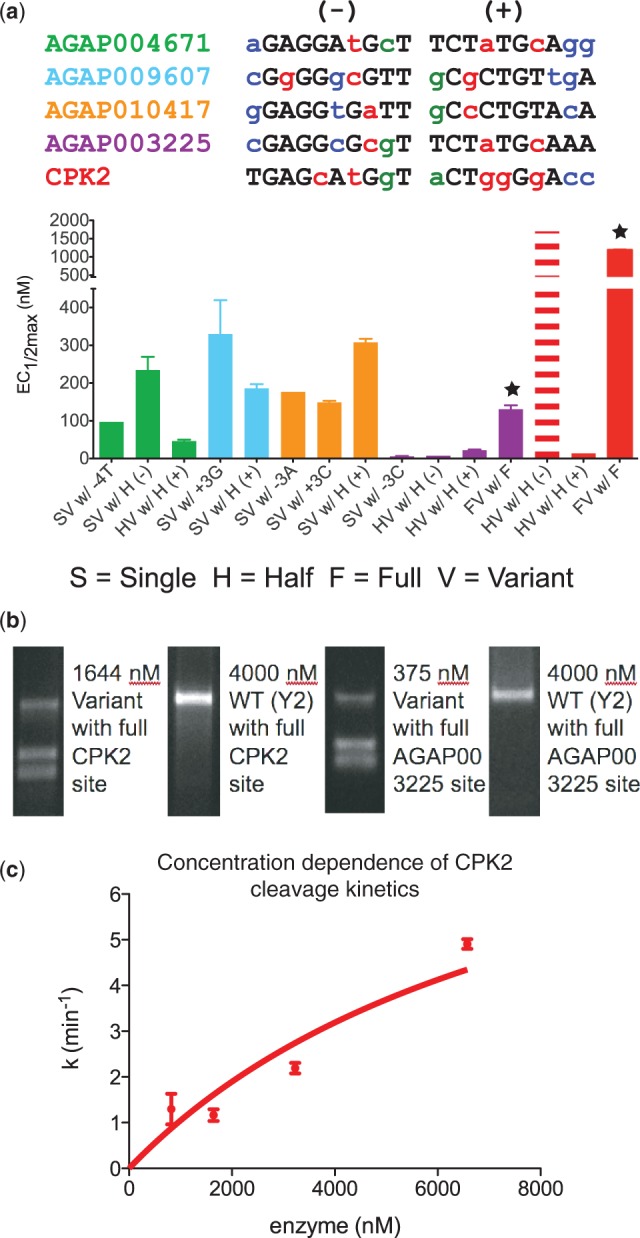
Cleaving full- and half-sites in genes associated with *Anopheles* sterility and genetic diseases. (**a**) Activity of variants selected against single base-pair substitutions (SV, single-site variant), half (– or +) (HV, half-site variant) and full-sites (FV, full-site variant) tested against the indicated target with *in vitro* cleavage assays. The color scheme for base-pair substitutions is the same as Table 1. Single variants are from the set of previously tested variants introduced in Figure 3, and sequences of all tested variants are in Supplementary Table S2. The dashed bars for the CPK2 HV w/ the (–) half-site indicate that an accurate EC_1/2max_-value could not be collected and this estimate was derived from data in Figure 5. Stars mark the two full variants shown to cleave their respective full-target sites. (**b**) Gel images showing specific cleavage of full target CPK2 and AGAP003225 sites by engineered enzymes, as well as lack of cleavage by the WT I-AniI scaffold (Y2) at even higher protein concentrations. Specificity for individual positions in the single base-pair changes in the AGAP003225 target and multiple base-pair changes in the CPK2 target are shown in, respectively, Figure 4 and Supplementary Figure S2. (**c**) Kinetic data for the endonuclease engineered to cleave the full CPK2 target. The rate of target site cleavage by the endonuclease engineered to cleave the full CPK2 target continues to increase above 3 uM indicating a high K_M_*.

The chosen *Anopheles* target sites also contain additional substitutions that are partially tolerated by the WT endonuclease (Figure 1b) and were not selected for, such as those in the central four and on the edges of the target site. It was unclear whether variants would exhibit context-dependent specificity changes, as observed for enzymes tested with the CPK1 and FAH1313 (+) half-sites, which would inhibit engineering for any of these targets. Thus, we tested five variants evolved to cleave different single base-pair substitutions present in *Anopheles* target genes (Table 1) and compared their cleavage activity against both the target with the singly substituted site and the *Anopheles* target half-sites containing the substitution as well as additional substitutions tolerated by the WT enzyme (Figure 6a). The majority of the variants cleaved both the singly substituted target and the half-sites with similar efficiency, and in one case (AGAP009607 (+)) was even more active against the latter. These results indicate that site context-dependence is not a complication for all target site combinations.

The half-site activity tests also indicated that the AGAP003225 and CPK2 targets were the most promising candidates for full-site cleavage, due to the strong activities obtained toward their (+) half-sites. We attempted to generate endonucleases to cleave these full-sites by combining the tested mutations for each half-site with no further engineering. Both the CPK2 and AGAP003225 sites were successfully cleaved (Figure 6a and b). The AGAP003225 site was cleaved relatively efficiently, with an EC_1/2max_ of 131 nM, comparable to many of the variants that successfully cleave single base-pair substitutions in the *in vivo* bacterial selection system. The variant targeting the CPK2 site cleaved its cognate target much less well; kinetics studies revealed that this enzyme has a *k*_cat_* of over 5 min^−^^1^, similar to the I-AniI endonuclease scaffold (Y2 variant) with a rate of 9.4 min^−^^1^ (12), but (Figure 6c) a K_M_* over 3 µM, compared to 81 nM for I-AniI. The high *k*_cat_* indicates that the catalytic center was successfully reconstituted when the two redesigned halves were combined, but further optimization is necessary to increase the overall affinity of the enzyme for the CPK2 site.

## DISCUSSION

We have engineered an extensive set of new I-AniI HE variants with distinct cleavage specificities (Figure 3). To generate these enzymes, we modified a bacterial selection system to select for higher specificity endonuclease variants while simultaneously improving system throughput (Figure 2). Guided by the observation that high specificity generally involved an amino acid sidechain that makes hydrogen bonds with the target base, we used a computational method for identifying such ‘motif’ interactions and used them to bias library design (Supplementary Figure S3, Figure 4). The combination of a highly efficient selection system with input from computational models in library design is an advance over previous approaches using random selection or computation alone (9,21,27,47,48).

These new methods, as well as the collection of data on protein–DNA interactions and catalysis, are applicable to the reprogramming of specificity for other nuclease families. For example, the CRISPR/Cas9 family of nucleases is becoming a popular catalyst for genome engineering (6,49–51), and the methods presented in this work could be used to increase the specificity of Cas9 (52–54) or expand the targeting potential of this class of enzymes, currently limited by the PAM sequence motif at the end of the target site (GG for Cas9) (5,55,56).

While our successes cleaving the CPK2 site and AGAP003225 site are promising, kinetic analysis of the CPK2 enzyme revealed that its binding affinity is suboptimal (Figure 6) and will require further improvements to become a useful gene therapy reagent. Determination of the crystal structure of the protein–DNA complex would greatly facilitate further rational design approaches to increasing affinity. Alternatively, the enzyme could be further optimized through selection, using either random mutagenesis across the entire protein sequence (32) or homolog-based sequence libraries (32,34,48). The large number of natural endonuclease homologs is a reservoir of potentially useful sequence information that can be used to improve specific properties of these enzymes; we discuss our progress using this information to improve I-AniI affinity in the Supplementary Material (Supplementary Figures S10 and S11, Supplementary Discussion B). Determining how to improve suboptimal enzymes would help to identify features of endonuclease function that are currently missing from our models.

We have identified several challenges that will need to be resolved for robust rational redesign of endonuclease specificity, notably unexpected context dependencies between base-pair substitutions (Figures 4, 5 and 6). While previous attempts to engineer the specificity of this nuclease family have also recognized this challenge (26,43,47), our kinetic analyses of the CPK1 target shows that indirect readout mechanisms extend far beyond the central four region of the substrate. Identifying the origins in the non-additivity in the effects of base substitutions on endonuclease cleavage, whether from the interactions between the N- and C-terminal domains (57,58), DNA bending at the center of the target site (43,59,60), or other causes is a current research challenge.

## SUPPLEMENTARY DATA

Supplementary Data are available at NAR Online, including [61–69].

## FUNDING

National Science Foundation (NSF) (graduate research fellowship to S.B.T.); US National Institutes of Health (NIH) and Foundation for the NIH through the Gates Foundation Grand Challenges in Global Health Initiative [GM084433, RL1CA133832 to D.B.]; Howard Hughes Medical Institute (HHMI). The content is solely the responsibility of the authors and does not necessarily represent the official views of the NIH. Funding for open access charge: NIH grants or HHMI funds (to D.B.).

*Conflict of interest statement*. None declared.

## Supplementary Material

Supplementary Data
