# Supplementary material for: Reprogramming homing endonuclease specificity through computational design and directed evolution
Source: Nucleic Acids Res. 2013 Nov 21;42(4):2564–76. doi: 10.1093/nar/gkt1212 (PMC3936771; doi:10.1093/nar/gkt1212)
Supplement: Supplementary Data [file supp_42_4_2564__index.html]

Reprogramming homing endonuclease specificity through computational design and directed evolution — Reprogramming homing endonuclease specificity through computational design and directed evolution — Supplementary Data 

# Reprogramming homing endonuclease specificity through computational design and directed evolution

## Supplementary Data

files

**Files in this Data Supplement:**

- Supplementary Data - pdf file
