# Supplementary material for: Reprogramming homing endonuclease specificity through computational design and directed evolution
Source: Nucleic Acids Res. 2013 Nov 21;42(4):2564–76. doi: 10.1093/nar/gkt1212 (PMC3936771; doi:10.1093/nar/gkt1212)
Supplement: Supplementary Data [file supp_gkt1212_nar-01480-v-2013-File009.pdf]

Supplementary Information

Contents:

Supplementary Methods

Supplementary Figures

- Figure S1. Control experiments for bacterial selection system and new negative selection component.
- Figure S2. Cleavage data for each newly selected endonuclease collected with *in vitro* assays.
- Figure S3. Examples of the strong hydrogen bonds in selected and designed I-AniI variants with high specificity.
- Figure S4. Computational specificity prediction for the I-AniI wild-type endonuclease.
- Figure S5. Detailed comparison of experimental and fixed-backbone computational specificities for the I-AniI wild-type endonuclease.
- Figure S6. Specificity prediction and recovery of engineered amino acids for selected I-AniI variants targeting single base-pair substitutions.
- Figure S7. Two computationally derived endonucleases targeting single base-pair changes can be combined without further optimization.
- Figure S8. Cleavage data for endonucleases targeting multiple base-pair pockets and full sites in genes of interest.
- Figure S9. Endonucleases targeting the (+) half-site changes in two mosquito sites show activity in human cells.
- Figure S10. Evidence for a role of C-terminal loop 190-199 in formation of the ground-state reaction complex.
- Figure S11. Mutations from I-AniI homologues that alter catalysis and binding.

Supplementary Discussion A. Rosetta computational predictions of experimental data.

Supplementary Discussion B. Improving the binding affinity of I-AniI.

Supplementary Tables

- Table S1. Activities, specificities, starting libraries, bacterial survivals, and computational predictions for all I-AniI variants shown in Figure S2 that target single base-pair substitutions.
- Table S2. Activities, specificities, starting libraries, and bacterial survivals for enzymes cleaving multiple base-pair pockets and full sites in genes of interest.

Supplementary Methods

Example target site primers with NheI and SacII sticky ends for building the pCcdB vector for single base-pair substitution selection:

Top primer, two copies of wild-type I-AniI LIB4 site:
ctagcTGAGGAGGTTaCTCTGTtAAgatacTGAGGAGGTTaCTCTGTtAAccgc
Bottom primer, two copies of wild-type I-AniI LIB4 site:
ggTTaACAGAGtAACCTCCTCAgtatcTTaACAGAGtAACCTCCTCAg
Top primer, -6C substitution:
ctagcTGAGcAGGTTaCTCTGTtAAgatacTGAGcAGGTTaCTCTGTtAAccgc

Primers used for amplifying the pCcdB plasmid to generate substrates for enzyme assays and for sequencing from pCcdB and pENDO-HE:

pEndo-SEQ-REV, AATGCTCTGCCAGTGTTACAACCA
pEndo-SEQ-FWD, CGGCGTCACACTTTGCTATG
pCcdB-SEQ-REV, TGCTGAAGCCAGTTACCTTCG
pCcdB-SEQ-FWD, CGAAGTGATCTTCCGTCACAGG
pCcdB-AMP-REV, CCCGACAGGACTATAAAGATACCAGGC
pCcdB-AMP-FWD, GAATTCCGGATGAGCATTCATCAGGC

Target site arrays:

Array containing a multiple base-pair substitution from the CPK2 (-) half-site, CPK2 (+) half-site:

```
TGAGGAGGTTTCatTGTAAA GCG
TGAGctGcTTTCTCTGTAAA TAGT
TGAGGAGGTTaCTggGTAAA CCCAG
TGAGccctTTTCTCTGTAAA AGT
TGAGGAGGTTTCTCacaAAA CTTATG
TGAGtAtGTTgCTCTGTAAA GGCAGG
TGAGGAGGTTTCTtTccAcA GCTC
TGAGttGGTTTCTCTGTAAA ATCCA
TGAGGAGGTTgCcCacaAAA TTAGC
TGAGcAtGTTTCTCTGTAAA GTCT
TGAGGAGGTTTCTCTccAAA GGTG
TGAGGccaTTgCTCTGTAAA CTTCTC
TGAGGAGGTTgCTtctTtcc AACG
gctGGAGGTTTCTCTGTAAA ACACGG
TGAGGAGGTTctTCTGaccA GAA
TGAGtctGTTaCTCTGTAAA
```

Array containing CPK2 (-) half-site, a multiple base-pair substitution region from the FAH1313 (+) half-site, CPK2 (+) half-site, and FAH1313 (+) half-site:

```
TGAGGAGGTTTCTCccTAAA GCG
ccAGtAtGTTgCTCTGTAAA TAGT
TGAGGAGGTTTCcCccTAgc CCCAG
TGAGcAtGgTaCTCTGTAAA AGT
TGAGGAGGTTaCTggGgAcc CTTATG
cGtGGccaTTgCTCTGTAAA GGCAGG
TGAGGAGGTTgCcCTGgAcA GCTC
gctGctGcTTTCTCTGTAAA ATCCA
TGAGGAGGTTTCTCTGaccA TTAGC
```

```
TGAGGAGGTTaCTaTGTttA GTCT
gGtGGttGcTTCTCTGTAAA GGTG
TGAGGAGagTTCTCTGTAAA CTTCTC
aGtGttGcTTTCTCTGTAAA AACG
TGAGGAGGTTTCatTGccAA ACACGG
TGAGGttGcTTCTCTGTAAA GAA
TGAGGAGGTTgCTtctTAAA
```

Array containing the CPK2 and FAH1313 full target sites:

```
TcCCtCTTATTCAACCTTTT GCG
TGAGccctTTcCcCccTAgc TAGT
TGAGcAtGgTaCTggGgAcc CCCAG
TTCCACTTATTCctgCagcT AGT
gctGctGcTTctTCTGaccA CTTATG
TGAGtctGTTaCTaTGTttA GGCAGG
gGtGGttGcTTCTtTccAcA GCTC
aGtGGtGGTTgCTtctTtcc ATCCA
TTCCACTTATTCAACCagcT TTAGC
gGtGGgtGcTaCTCctaAtg GTCT
TGAGGAGGTTaCTCctaAtg GGTG
gGtGGgtGcTaCTCTGTAAA CTTCTC
TcCCtCTTATTCctgCagcT AACG
cGtGGccaTTgCcCTGgAcA ACACGG
aGtGttGcTTTCatTGccAA GAA
TTCCACTTATTCctgCTTTT
```

Two arrays containing AGAP half- and full- sites:

```
aGAGGcGGTTTCTCgcTAcg ACGT
gGcGGAcGTTTCTCTGTAAA TATCG
TGAGGAGGTTTCTaTGcAAA CGAT
TGgGGtGcTTcCTtcGTAAt GTACA
TGAGGAGGTTcCcCaGTAgc GATC
gGtGGcGGTTTCatTcTctA AACTG
gGtGGcGtcTTCTggGTgAA ATCG
gGAGGtGaTTgCcCTGTAcA TACGC
TGAGGAGGTTTCatTcTAgg GATC
aGAGGAtGcTTCTaTGcAgg TACGT
TGAGGAGGTTgCgCTGTtgA ATAC
cGAGGAGGcTTCaCTtTcAt CGATA
cGgGGgcGTTTCTCTGTAAA GATC
cGAGGcGcgTTCTCTGTAAA TAGAA
TGAGGAGGTTTCTgaGTtgc GCGAT
cGAGGtGGaTTCTCTGTAAA
```

```
cGgGGgcGTTgCgCTGTtgA ACGT
cGAGGcGcgTTCTaTGcAAA TATCG
TGgGGtGcTTTCTCTGTAAA CGAT
gGcGGAcGTTTCTgaGTtgc GTACA
gGAGGAGaTTcCcCaGTAgc GATC
gGAGGtGaTTTCTCTGTAAA AACTG
TGAGGAGGTTTCTggGTgAA ATCG
cGAGGtGGaTTCatTcTAgg TACGC
aGAGGAtGcTTCTCTGTAAA GATC
TGAGGAGGTTTCaCTtTcAt TACGT
TGAGGAGGTTTCTaTGcAgg ATAC
TGAGGAGGTTTCatTcTctA CGATA
TGAGGAGGTTcCTtcGTAAt GATC
gGtGGcGtcTTCTCTGTAAA TAGAA
TGAGGAGGTTTCTCgcTAcg GCGAT
TGAGGAGGTTgCcCTGTAcA
```

Sequences of vectors:

pENDO-HE, containing wild-type I-AniI sequence between NcoI and NotI sites:

```
GTTGACGCCGGGCAAGAGCAACTCGGTCGCCGCATACACTATTCTCAGAATGACTTGGTTGAGTACTCACCAGTCACAGAAAAGCATCTTACGGATG
GCATGACAGTAAGAGAATTATGCAGTGCTGCCATAACCATGAGTGATAACACTGCGGCCAACTTACTTCTGACAACGATCGGAGGACCGAAGGAGCT
AACCGCTTTTTTGCACAACATGGGGGATCATGTAACTCGCCTTGATCGTTGGGAACCGGAGCTGAATGAAGCCATACCAAACGACGAGCGTGACACC
ACGATGCCTGCAGCAATGGCAACAACGTTGCGCAAACTATTAACTGGCGAACTACTTACTCTAGCTTCCCGGCAACAATTAATAGACTGGATGGAGG
CGGATAAAGTTGCAGGACCACTTCTGCGCTCGGCCCTTCCGGCTGGCTGGTTTATTGCTGATAAATCTGGAGCCGGTGAGCGTGGGTCTCGCGGTAT
CATTGCAGCACTGGGGCCAGATGGTAAGCCCTCCCGTATCGTAGTTATCTACACGACGGGGAGTCAGGCAACTATGGATGAACGAAATAGACAGATC
GCTGAGATAGGTGCCTCACTGATTAAGCATTGGTAACTGTCAGACCAAGTTTACTCATATATACTTTAGATTGATTTAAAACTTCATTTTTAATTTA
AAAGGATCTAGGTGAAGATCCTTTTTGATAATCTCATGACCAAAATCCCTTAACGTGAGTTTTCGTTCCACTGAGCGTCAGACCCCTTAATAAGATG
ATCTTCTTGAGATCGTTTTGGTCTGCGCGTAATCTCTTGCTCTGAAAACGAAAAAACCGCCTTGCAGGGCGGTTTTTCGAAGGTTCTCTGAGCTACC
AACTCTTTGAACCGAGGTAACTGGCTTGGAGGAGCGCAGTCACCAAAACTTGTCCTTTCAGTTTAGCCTTAACCGGCGCATGACTTCAAGACTAACT
CCTCTAAATCAATTACCAGTGGCTGCTGCCAGTGGTGCTTTTGCATGTCTTTCCGGGTTGGACTCAAGACGATAGTTACCGGATAAGGCGCAGCGGT
CGGACTGAACGGGGGGTTCGTGCATACAGTCCAGCTTGGAGCGAACTGCCTACCCGGAACTGAGTGTCAGGCGTGGAATGAGACAAACGCGGCCATA
ACAGCGGAATGACACCGGTAAACCGAAAGGCAGGAACAGGAGAGCGCACGAGGGAGCCGCCAGGGGGAAACGCCTGGTATCTTTATAGTCCTGTCGG
GTTTCGCCACCACTGATTTGAGCGTCAGATTTCGTGATGCTTGTCAGGGGGGCGGAGCCTATGGAAAAACGGCTTTGCCGCGGCCCTCTCACTTCCC
TGTTAAGTATCTTCCTGGCATCTTCCAGGAAATCTCCGCCCCGTTCGTAAGCCATTTCCGCTCGCCGCAGTCGAACGACCGAGCGTAGCGAGTCAGT
GAGCGAGGAAGCGGAATATATCCTGTATCACATATTCTGCTGACGCACCGGTGCAGCCTTTTTTCTCCTGCCACATGAAGCACTTCACTGACACCCT
```

CATCAGTGCCAACATAGTAAGCCAGTATACACTCCGCTAGCGCTGAGGTCTGCCTCGTGAAGAAGGTGTTGCTGACTCATACCAGGCCTGAATCGCC
CCATCATCCAGCCAGAAAGTGAGGGAGCCACGGTTGATGAGAGCTTTGTTGTAGGTGGACCAGTTGGTGATTTTGAACTTTTGCTTTGCCACGGAAC
GGTCTGCGTTGTCGGCATGCGCATAATGTGCCTGTCAAATGGACGAAGCAGGGATTCTGCAAACCCTATGCTACTCCGTCAAGCCGTCAATTGTCTG
ATTCGTTACCAATTATGACAACTTGACGGCTACATCATTCACTTTTTCTTCACAACCGGCACGGAACTCGCTCGGGCTGGCCCCGGTGCATTTTTTA
AATACCCGCGAGAAATAGAGTTGATCGTCAAAACCAACATTGCGACCGACGGTGGCGATAGGCATCCGGGTGGTGCTCAAAAGCAGCTTCGCCTGGC
TGATACGTTGGTCCTCGCGCCAGCTTAAGACGCTAATCCCTAACTGCTGGCGGAAAAGATGTGACAGACGCGACGGCGACAAGCAAACATGCTGTGC
GACGCTGGCGATATCAAAATTGCTGTCTGCCAGGTGATCGCTGATGTACTGACAAGCCTCGCGTACCCGATTATCCATCGGTGGATGGAGCGACTCG
TTAATCGCTTCCATGCGCCGCAGTAACAATTGCTCAAGCAGATTTATCGCCAGCAGCTCCGAATAGCGCCCTTCCCCTTGCCCGGCGTTAATGATTT
GCCCAAACAGGTCGCTGAAATGCGGCTGGTGCGCTTCATCCGGGCGAAAGAACCCCGTATTGGCAAATATTGACGGCCAGTTAAGCCATTCATGCCA
GTAGGCGCGCGGACGAAAGTAAACCCACTGGTGATACCATTCGCGAGCCTCCGGATGACGACCGTAGTGATGAATCTCTCCTGGCGGGAACAGCAAA
ATATCACCCGGTCGGCAAACAAATTCTCGTCCCTGATTTTTCACCACCCCCTGACCGCGAATGGTGAGATTGAGAATATAACCTTTCATTCCCAGCG
GTCGGTCGATAAAAAAATCGAGATAACCGTTGGCCTCAATCGGCGTTAAACCCGCCACCAGATGGGCATTAAACGAGTATCCCGGCAGCAGGGGATC
ATTTTGCGCTTCAGCCATACTTTTCATACTCCCGCCATTCAGAGAAGAAACCAATTGTCCATATTGCATCAGACATTGCCGTCACTGCGTCTTTTAC
TGGCTCTTCTCGCTAACCAAACCGGTAACCCCGCTTATTAAAAGCATTCTGTAACAAAGCGGGACCAAAGCCATGACAAAAACGCGTAACAAAAGTG
TCTATAATCACGGCAGAAAAGTCCACATTGATTATTTGCACGGCGTCACACTTTGCTATGCCATAGCATTTTTATCCATAAGATTAGCGGATCCTAC
CTGACGCTTTTTATCGCAACTCTCTACTGTTTCTCCATACCCGTTTTTTTGGGCTAGAAATAATTTTGTTTAACTTTAAGAAGGAGATATACCCATG
GGATCCGATCTTACATATGCCTACCTGGTAGGATTGTTCGAAGGTGATGGTTATTTCTCCATTACAAAGAAAGGTAAGTATCTGACCTATGAGCTGG
GTATTGAGCTGTCAATTAAGGATGTACAATTGATTTACAAGATCAAGAAGATTCTGGGAATTGGTATTGTAAGTTTTCGCAAACGCAATGAGATCGA
GATGGTAGCCCTGCGCATCCGCGACAAGAACCATCTGAAAAGTAAAATTCTGCCTATCTTTGAGAAATATCCCATGTTCTCTAATAAGCAATATGAC
TATCTGCGCTTTCGCAATGCACTGCTTTCAGGTATTATTTCTCTGGAAGATCTGCCTGATTATACTCGCAGTGATGAGCCTCTGAATTCTATCGAGT
CGATTATCAACACATCTTACTTCTCTGCCTGGCTGGTAGGCTTTATCGAAGCTGAAGGTTGTTTCAGCGTTTACAAACTGAACAAAGATGATGATTA
CCTGATCGCTAGTTTTGATATTGCTCAACGCGATGGGGATATTCTGATCTCAGCCATCCGTAAATACCTGTCTTTCACTACTAAAGTTTACCTGGAT
AAAACTAACTGTTCAAAGCTGAAAGTTACAAGTGTACGCTCAGTAGAGAACATCATTAAGTTCCTGCAAAACGCACCTGTAAAACTGCTGGGTAACA
AGAAGCTGCAATACCTGAAGTGGCTGAAACAGCTGCGTAAGATCTCTCGCTACTCAGAAAAGATCAAGATCCCTTCAAACTACTAAGCGGCCGCTCA
GAATTGGTTAATTGGTTGTAACACTGGCAGAGCATTACGCTGACTTGACGGGACGGCGGCTTTGTTGAATAAATCGAACTTTTGCTGAGTTGAAGGA
TCAGATCACGCATCTTCCCGACAACGCAGACCGTTCCGTGGCAAAGCAAAAGTTCAAAATCACCAACTGGTCCACCTACAACAAAGCTCTCATCAAC
CGTGGCTCCCTCACTTTCTGGCTGGATGATGGGGCGATTCAGGCCTGGTATGAGTCAGCAACACCTTCTTCACGAGGCAGACCTCAGCGCTCAAAGA
TGCAGGGGTAAAAGCTAACCGCATCTTTACCGACAAGGCATCCGGCAGTTCAACAGATCGGGAAGGGCTGGATTTGCTGAGGATGAAGGTGGAGGAA
GGTGATGTCATTCTGGTGAAGAAGCTCGACCGTCTTGGCCGCGACACCGCCGACATGATCCAACTGATAAAAGAGTTTGATGCTCAGGGTGTAGCGG
TTCGGTTTATTGACGACGGGATCAGTACCGACGGTGATATGGGGCAAATGGTGGTCACCATCCTGTCGGCTGTGGCACAGGCTGAACGCCGGAGGAT
CCTAGAGCGCACGAATGAGGGCCGACAGGAAGCAAAGCTGAAAGGAATCAAATTTGGCCGCAGGCGTACCGTGGACAGGAACGTCGTGCTGACGCTT
CATCAGAAGGGCACTGGTGCAACGGAAATTGCTCATCAGCTCAGTATTGCCCGCTCCACGGTTTATAAAATTCTTGAAGACGAAAGGGCCTCGTGAT
ACGCCTATTTTTATAGGTTAATGTCATGATAATAATGGTTTCTTAGACGTCAGGTGGCACTTTTCGGGGAAATGTGCGCGGAACCCCTATTTGTTTA
TTTTTCTAAATACATTCAAATATGTATCCGCTCATGAGACAATAACCCTGATAAATGCTTCAATAATATTGAAAAAGGAAGAGTATGAGTATTCAAC
ATTTCCGTGTCGCCCTTATTCCCTTTTTTGCGGCATTTTGCCTTCCTGTTTTTGCTCACCCAGAAACGCTGGTGAAAGTAAAAGATGCTGAAGATCA
GTTGGGTGCACGAGTGGGTTACATCGAACTGGATCTCAACAGCGGTAAGATCCTTGAGAGTTTTCGCCCCGAAGAACGTTTTCCAATGATGAGCACT
TTTAAAG

pCcdB, containing wild-type I-AniI target site between NheI and SacII:

ATCGATGCATAATGTGCCTGGACGTCGCGCAACGCAATTAATGTGAGTTAGCTCACTCATTAGGCACCCCAGGCTTTACACTTTATGCTTCCGGCTC
GTATGTTGTGTGGAATTGTGAGCGAATAACAATTTCACACAGGAAACAGCTATGACCATGATTACGCCAAGCTATTTAGGTGACACTATAGAATACT
CAAGCTATGCATCAAGCTTGGTACCGAGCTCGGATCCACTAGTAACGGCCGCCAGTGTGCTGGAATTCTGCAGATATCCATCACACTGGCGGCCGCT
CGAGCATGCATCTAGAGGGCCCAATTCGCCCTATTCGAAGTCGTATTACAATTCACTGGCCGTCGTTTTACAACGTCGTGACTGGGAAAACCCTGGC
GTTACCCAACTTAATCGCCTTGCAGCACATCCCCCTTTCGCCAGCTGGCGTAATAGCGAAGAGGCCCGCACCGATCGCCCTTCCCAACAGTTGCGCA
GCCTATACGTACGGCAGTTTAAGGTTTACACCTATAAAAGAGAGAGCCGTTATCGTCTGTTTGTGGATGTACAGAGTGATATTATTGACACGCCGGG
GCGACGGATGGTGATCCCCCTGGCCAGTGCACGTCTGCTGTCAGATAAAGTCTCCCGTGAACTTTACCCGGTGGTGCATATCGGGGATGAAAGCTGG
CGCATGATGACCACCGATATGGCCAGTGTGCCGGTCTCCGTTATCGGGGAAGAAGTGGCTGATCTCAGCCACCGCGAAAATGACATCAAAAACGCCA
TTAACCTGATGTTCTGGGGAATATAAGGCGCGCCTTTACAAAAATCAGATAAACGTGTAGATCTCGGATCAAACGCCATGAGCGGCCTCATTTCTTA
TTCTGAGTTACAACAGTCCGCACCGCTGTCCGGTAGCTCCTTCCGGTGGGCGCGGGGCATGACTATCGTCGCCGCACTTATGACTGTCTTCTTTATC
ATGCAACTCGTAGGACAGGTGCCGGCAGCGCCCAACAGTCCCCCGGCCACGGGGCCTGCCACCATACCCACGCCGAAACAAGCGCCCTGCACCATTA
TGTTCCGGATCTGCATCGCAGGATGCTGCTGGCTACCCTGTGGAACACCTACATCTGTATTAACGAAGCGCTAACCGTTTTTATCAGGCTCTGGGAG
GCAGAATAAATGATCATATCGTCAATTATTACCTCCACGGGGAGAGCCTGAGCAAACTGGCCTCAGGCATTTGAGAAGCACACGGTCACACTGCTTC
CGGTAGTCAATAAACCGGTAAACCAGCAATAGACATAAGCGGCTATTTAACGACCCTGCCCTGAACCGACGACCGGGTCGAATTTGCTTTCGAATTT
CTGCCATTCATCCGCTTATTATCACTTATTCAGGCGTAGCACCAGGCGTTTAAGGGCACCAATAACTGCCTTAAAAAATTACGCCCCGCCCTGCCA
CTCATCGCAGTACTGTTGTAATTCATTAAGCATTCTGCCGACATGGAAGCCATCACAGACGGCATGATGAACCTGAATCGCCAGCGGCATCAGCACC
TTGTCGCCTTGCGTATAATATTTGCCCATGGTGAAAACGGGGGCGAAGAAGTTGTCCATATTGGCCACGTTTAAATCAAAACTGGTGAAACTCACCC
AGGGATTGGCTGAGACGAAAAACATATTCTCAATAAACCCTTTAGGGAAATAGGCCAGGTTTTCACCGTAACACGCCACATCTTGCGAATATATGTG
TAGAAACTGCCGGAAATCGTCGTGGTATTCACTCCAGAGCGATGAAAACGTTTCAGTTTGCTCATGGAAAACGGTGTAACAAGGGTGAACACTATCC
CATATCACCAGCTCACCGTCTTTCATTGCCATACGGAATTCCGGATGAGCATTCATCAGGCGGGCAAGAATGTGAATAAAGGCCGGATAAAACTTGT
GCTTATTTTTCTTTACGGTCTTTAAAAAGGCCGTAATATCCAGCTGAACGGTCTGGTTATAGGTACATTGAGCAACTGACTGAAATGCCTCAAAATG
TTCTTTACGATGCCATTGGGATATATCAACGGTGGTATATCCAGTGATTTTTTTCTCCATTTTAGCTTCCTTAGCTCCTGAAAATCTCGATAACTTT
ATTTCATTATGGTGAAAGTTGGAACCTCTTACGTGCCGATCAACGTCTCATTTTCGCCAAAAGTTGGCCCAGGGCTTCCCGGTATCAACAGGGACAC
CAGGATTTATTTATTCTGCGAAGTGATCTTCCGTCACAGGTATTTATTCGGCGCAAAGTGCGTCGGGTGATGCTGCCAACTTACTGATTTAGTGTAT
GATGGTGTTTTTGAGGTGCTCCAGTGGCTTCTGTTTCTATCAGCTGTCCCTCCTGTTCAGCTACTGACGGGGTGGTGCGTAACGGCAAAAGCACCGC
CGGACATCAGCGCTAGCTGAGGAGGTTTCTCTGTAATGAGGAGGTTTCTCTGTAACCGCGGAGACAGATCGCTGAGATAGGTGCCTCACTGATTAAG
CATTGGTAACTGTCAGACCAAGTTTACTCATATATACTTTAGATTGATTTAAAACTTCATTTTTAATTTAAAAGGATCTAGGTGAAGATCCTTTTTG
ATAATCTCATGACCAAAATCCCTTAACGTGAGTTTTCGTTCCACTGAGCGTCAGACCCCGTAGAAAAGATCAAAGGATCTTCTTGAGATCCTTTTTT
TCTGCGCGTAATCTGCTGCTTGCAAACAAAAAAACCACCGCTACCAGCGGTGGTTTGTTTGCCGGATCAAGAGCTACCAACTCTTTTTCCGAAGGTA
ACTGGCTTCAGCAGAGCGCAGATACCAAATACTGTCCTTCTAGTGTAGCCGTAGTTAGGCCACCACTTCAAGAACTCTGTAGCACCGCCTACATACC

TCGCTCTGCTAATCCTGTTACCAGTGGCTGCTGCCAGTGGCGATAAGTCGTGTCTTACCGGGTTGGACTCAAGACGATAGTTACCGGATAAGGCGCA
GCGGTCGGGCTGAACGGGGGGTTCGTGCACACAGCCCAGCTTGGAGCGAACGACCTACACCGAACTGAGATACCTACAGCGTGAGCTATGAGAAAGC
GCCACGCTTCCCGAAGGGAGAAAGGCGGACAGGTATCCGGTAAGCGGCAGGGTCGGAACAGGAGAGCGCACGAGGGAGCTTCCAGGGGGAAACGCCT
GGTATCTTTATAGTCCTGTCGGGTTTCGCCACCTCTGACTTGAGCGTCGATTTTTGTGATGCTCGTCAGGGGG

pET15-HE, ORFs were inserted between NcoI and NotI sites:

TTCTCATGTTTGACAGCTTATCATCGATAAGCTTTAATGCGGTAGTTTATCACAGTTAAATTGCTAACGCAGTCAGGCACCGTGTATGAAATCTAAC
AATGCGCTCATCGTCATCCTCGGCACCGTCACCCTGGATGCTGTAGGCATAGGCTTGGTTATGCCGGTACTGCCGGGCCTCTTGCGGGATATCCGGA
TATAGTTCCTCCTTTCAGCAAAAAACCCCTCAAGACCCGTTTAGAGGCCCCAAGGGGTTATGCTAGTTATTGCTCAGCGGTGGCAGCAGCCAACTCA
GCTTCCTTTCGGGCTTTGTTAGCAGCCGGATCCTCGAGCTAGCGGCCGCTAAGCCTGAGGCTAGCGCCATGGAGCTGCCGCGCGGCACCAGGCCGCT
GCTATGATGATGATGATGATGGCTGCTAGCCATAGTATATCTCCTTCTTAAAGTTAAACAAAATTATTTCTAGAGGGGAATTGTTATCCGCTCACAA
TTCCCCTATAGTGAGTCGTATTAATTTCGCGGGATCGAGATCTCGATCCTCTACGCCGGACGCATCGTGGCCGGCATCACCGGCGCCACAGGTGCGG
TTGCTGGCGCCTATATCGCCGACATCACCGATGGGGAAGATCGGGCTCGCCACTTCGGGCTCATGAGCGCTTGTTTCGGCGTGGGTATGGTGGCAGG
CCCCGTGGCCGGGGGACTGTTGGGCGCCATCTCCTTGCATGCACCATTCCTTGCGGCGGCGGTGCTCAACGGCCTCAACCTACTACTGGGCTGCTTC
CTAATGCAGGAGTCGCATAAGGGAGAGCGTCGAGATCCCGGACACCATCGAATGGCGCAAAACCTTTCGCGGTATGGCATGATAGCGCCCGGAAGAG
AGTCAATTCAGGGTGGTGAATGTGAAACCAGTAACGTTATACGATGTCGCAGAGTATGCCGGTGTCTCTTATCAGACCGTTTCCCGCGTGGTGAACC
AGGCCAGCCACGTTTCTGCGAAAACGCGGGAAAAAGTGGAAGCGGCGATGGCGGAGCTGAATTACATTCCCAACCGCGTGGCACAACAACTGGCGGG
CAAACAGTCGTTGCTGATTGGCGTTGCCACCTCCAGTCTGGCCCTGCACGCGCCGTCGCAAATTGTCGCGGCGATTAAATCTCGCGCCGATCAACTG
GGTGCCAGCGTGGTGGTGTCGATGGTAGAACGAAGCGGCGTCGAAGCCTGTAAAGCGGCGGTGCACAATCTTCTCGCGCAACGCGTCAGTGGGCTGA
TCATTAACTATCCGCTGGATGACCAGGATGCCATTGCTGTGGAAGCTGCCTGCACTAATGTTCCGGCGTTATTTCTTGATGTCTCTGACCAGACACC
CATCAACAGTATTATTTTCTCCCATGAAGACGGTACGCGACTGGGCGTGGAGCATCTGGTCGCATTGGGTCACCAGCAAATCGCGCTGTTAGCGGGC
CCATTAAGTTCTGTCTCGGCGCGTCTGCGTCTGGCTGGCTGGCATAAATATCTCACTCGCAATCAAATTCAGCCGATAGCGGAACGGGAAGGCGACT
GGAGTGCCATGTCCGGTTTTCAACAAACCATGCAAATGCTGAATGAGGGCATCGTTCCCACTGCGATGCTGGTTGCCAACGATCAGATGGCGCTGGG
CGCAATGCGCGCCATTACCGAGTCCGGGCTGCGCGTTGGTGCGGATATCTCGGTAGTGGGATACGACGATACCGAAGACAGCTCATGTTATATCCCG
CCGTTAACCACCATCAAACAGGATTTTCGCCTGCTGGGGCAAACCAGCGTGGACCGCTTGCTGCAACTCTCTCAGGGCCAGGCGGTGAAGGGCAATC
AGCTGTTGCCCGTCTCACTGGTGAAAAGAAAAACCACCCTGGCGCCCAATACGCAAACCGCCTCTCCCCGCGCGTTGGCCGATTCATTAATGCAGCT
GGCACGACAGGTTTCCCGACTGGAAAGCGGGCAGTGAGCGCAACGCAATTAATGTAAGTTAGCTCACTCATTAGGCACCGGGATCTCGACCGATGCC
CTTGAGAGCCTTCAACCCAGTCAGCTCCTTCCGGTGGGCGCGGGGCATGACTATCGTCGCCGCACTTATGACTGTCTTCTTTATCATGCAACTCGTA
GGACAGGTGCCGGCAGCGCTCTGGGTCATTTTCGGCGAGGACCGCTTTCGCTGGAGCGCGACGATGATCGGCCTGTCGCTTGCGGTATTCGGAATCT
TGCACGCCCTCGCTCAAGCCTTCGTCACTGGTCCCGCCACCAAACGTTTCGGCGAGAAGCAGGCCATTATCGCCGGCATGGCGGCCGACGCGCTGGG
CTACGTCTTGCTGGCGTTCGCGACGCGAGGCTGGATGGCCTTCCCCATTATGATTCTTCTCGCTTCCGGCGGCATCGGGATGCCCGCGTTGCAGGCC
ATGCTGTCCAGGCAGGTAGATGACGACCATCAGGGACAGCTTCAAGGATCGCTCGCGGCTCTTACCAGCCTAACTTCGATCACTGGACCGCTGATCG
TCACGGCGATTTATGCCGCCTCGGCGAGCACATGGAACGGGTTGGCATGGATTGTAGGCGCCGCCCTATACCTTGTCTGCCTCCCCGCGTTGCGTCG
CGGTGCATGGAGCCGGGCCACCTCGACCTGAATGGAAGCCGGCGGCACCTCGCTAACGGATTCACCACTCCAAGAATTGGAGCCAATCAATTCTTGC
GGAGAACTGTGAATGCGCAAACCAACCCTTGGCAGAACATATCCATCGCGTCCGCCATCTCCAGCAGCCGCACGCGGCGCATCTCGGGCAGCGTTGG
GTCCTGGCCACGGGTGCGCATGATCGTGCTCCTGTCGTTGAGGACCCGGCTAGGCTGGCGGGGTTGCCTTACTGGTTAGCAGAATGAATCACCGATA
CGCGAGCGAACGTGAAGCGACTGCTGCTGCAAAACGTCTGCGACCTGAGCAACAACATGAATGGTCTTCGGTTTCCGTGTTTCGTAAAGTCTGGAAA
CGCGGAAGTCAGCGCCCTGCACCATTATGTTCCGGATCTGCATCGCAGGATGCTGCTGGCTACCCTGTGGAACACCTACATCTGTATTAACGAAGCG
CTGGCATTGACCCTGAGTGATTTTTCTCTGGTCCCGCCGCATCCATACCGCCAGTTGTTTACCCTCACAACGTTCCAGTAACCGGGCATGTTCATCA
TCAGTAACCCGTATCGTGAGCATCCTCTCTCGTTTCATCGGTATCATTACCCCCATGAACAGAAATCCCCCTTACACGGAGGCATCAGTGACCAAAC
AGGAAAAAACCGCCCTTAACATGGCCCGCTTTATCAGAAGCCAGACATTAACGCTTCTGGAGAAACTCAACGAGCTGGACGCGGATGAACAGGCAGA
CATCTGTGAATCGCTTCACGACCACGCTGATGAGCTTTACCGCAGCTGCCTCGCGCGTTTCGGTGATGACGGTGAAAACCTCTGACACATGCAGCTC
CCGGAGACGGTCACAGCTTGTCTGTAAGCGGATGCCGGGAGCAGACAAGCCCGTCAGGGCGCGTCAGCGGGTGTTGGCGGGTGTCGGGGCGCAGCCA
TGACCCAGTCACGTAGCGATAGCGGAGTGTATACTGGCTTAACTATGCGGCATCAGAGCAGATTGTACTGAGAGTGCACCATATATGCGGTGTGAAA
TACCGCACAGATGCGTAAGGAGAAAATACCGCATCAGGCGCTCTTCCGCTTCCTCGCTCACTGACTCGCTGCGCTCGGTCGTTCGGCTGCGGCGAGC
GGTATCAGCTCACTCAAAGGCGGTAATACGGTTATCCACAGAATCAGGGGATAACGCAGGAAAGAACATGTGAGCAAAAGGCCAGCAAAAGGCCAGG
AACCGTAAAAAGGCCGCGTTGCTGGCGTTTTTCCATAGGCTCCGCCCCCCTGACGAGCATCACAAAAATCGACGCTCAAGTCAGAGGTGGCGAAACC
CGACAGGACTATAAAGATACCAGGCGTTTCCCCCTGGAAGCTCCCTCGTGCGCTCTCCTGTTCCGACCCTGCCGCTTACCGGATACCTGTCCGCCTT
TCTCCCTTCGGGAAGCGTGGCGCTTTCTCATAGCTCACGCTGTAGGTATCTCAGTTCGGTGTAGGTCGTTCGCTCCAAGCTGGGCTGTGTGCACGAA
CCCCCCGTTCAGCCCGACCGCTGCGCCTTATCCGGTAACTATCGTCTTGAGTCCAACCCGGTAAGACACGACTTATCGCCACTGGCAGCAGCCACTG
GTAACAGGATTAGCAGAGCGAGGTATGTAGGCGGTGCTACAGAGTTCTTGAAGTGGTGGCCTAACTACGGCTACACTAGAAGGACAGTATTTGGTAT
CTGCGCTCTGCTGAAGCCAGTTACCTTCGGAAAAAGAGTTGGTAGCTCTTGATCCGGCAAACAAACCACCGCTGGTAGCGGTGGTTTTTTTGTTTGC
AAGCAGCAGATTACGCGCAGAAAAAAAGGATCTCAAGAAGATCCTTTGATCTTTTCTACGGGGTCTGACGCTCAGTGGAACGAAAACTCACGTTAAG
GGATTTTGGTCATGAGATTATCAAAAAGGATCTTCACCTAGATCCTTTTAAATTAAAAATGAAGTTTTAAATCAATCTAAAGTATATATGAGTAAAC
TTGGTCTGACAGTTACCAATGCTTAATCAGTGAGGCACCTATCTCAGCGATCTGTCTATTTCGTTCATCCATAGTTGCCTGACTCCCCGTCGTGTAG
ATAACTACGATACGGGAGGGCTTACCATCTGGCCCCAGTGCTGCAATGATACCGCGAGACCCACGCTCACCGGCTCCAGATTTATCAGCAATAAACC
AGCCAGCCGGAAGGGCCGAGCGCAGAAGTGGTCCTGCAACTTTATCCGCCTCCATCCAGTCTATTAATTGTTGCCGGGAAGCTAGAGTAAGTAGTTC
GCCAGTTAATAGTTTGCGCAACGTTGTTGCCATTGCTGCAGGCATCGTGGTGTCACGCTCGTCGTTTGGTATGGCTTCATTCAGCTCCGGTTCCCAA
CGATCAAGGCGAGTTACATGATCCCCCATGTTGTGCAAAAAAGCGGTTAGCTCCTTCGGTCCTCCGATCGTTGTCAGAAGTAAGTTGGCCGCAGTGT
TATCACTCATGGTTATGGCAGCACTGCATAATTCTCTTACTGTCATGCCATCCGTAAGATGCTTTTCTGTGACTGGTGAGTACTCAACCAAGTCATT
CTGAGAATAGTGTATGCGGCGACCGAGTTGCTCTTGCCCGGCGTCAACACGGGATAATACCGCGCCACATAGCAGAACTTTAAAAGTGCTCATCATT
GGAAAACGTTCTTCGGGGCGAAAACTCTCAAGGATCTTACCGCTGTTGAGATCCAGTTCGATGTAACCCACTCGTGCACCCAACTGATCTTCAGCAT
CTTTTACTTTCACCAGCGTTTCTGGGTGAGCAAAAACAGGAAGGCAAAATGCCGCAAAAAAGGGAATAAGGGCGACACGGAAATGTTGAATACTCAT
ACTCTTCCTTTTTCAATATTATTGAAGCATTTATCAGGGTTATTGTCTCATGAGCGGATACATATTTGAATGTATTTAGAAAAATAAACAAATAGGG
GTTCCGCGCACATTTCCCCGAAAAGTGCCACCTGACGTCTAAGAAACCATTATTATCATGACATTAACCTATAAAAATAGGCGTATCACGAGGCCCT
TTCGTCTTCAAGAA

Computational modeling

Specificity prediction

Specificity was calculated with the publicly available application rosettaDNA (26, 61). For these calculations, the variant sequence (or wild-type sequence) was modeled with each of the four possible nucleotides at the DNA position of interest and the total energy of the complex was computed. Residues in a 6 Å region (defined by the z_cutoff flag in the command line arguments) surrounding the target base-pair were allowed to repack, meaning they could change in conformation but not in sequence. A total of 56 models were predicted for each nucleotide, and the average energy over all 56 was used in the specificity calculation. Specificity was defined for the computational models by the Boltzmann weight of the specific complex, as follows and as in previous work (21, 26, 61):

$Specificity_n = [e\verb|^|(-(E_n-E_{target}) / k_BT)] / \Sigma[e\verb|^|(-(E_n-E_{target}) / k_BT)]$, where the sum is over the quantity in the numerator for all four bases. The value of k_BT was set to 1 based on previous work correlating Rosetta Energy Units (REUs) with kcal/mol (62).

For example, the predicted energies of the models for a single run of the -8G_P1 variant are -727.782 for an adenine substitution, -725.740 for cytosine, -729.516 for guanine, -713.981 for thymine. The calculation of specificity for adenine is as follows:

$Specificity_{ade} = [e\verb|^|(-(-727.782 + 729.516) / 1)] / ([e\verb|^|(-(-727.782 + 729.516) / 1)] + [e\verb|^|(-(-725.740 + 729.516) / 1)] + [e\verb|^|(-(-729.516 + 729.516) / 1)] + [e\verb|^|(-(-727.713.981 + 729.516) / 1)])$ = 0.147 for adenine (and if the calculation is repeated for the other bases: 0.019 for cytosine, 0.8336 for guanine, and 1.49E-07 for thymine)

Design calculation

In order to assess whether the computational design methods could recover selected amino acids, design was completed for all single base-pair substitutions in the I-AniI target site. The designed amino acids were compared to the amino acids derived from selection for the same base-pair substitutions. The motif-biased design data were collected with the motif_dna_packer_design application (33). However, this version of the protocol is written with trunk Rosetta and includes some minor changes over the trunk version previously described (33) improved the results, making them more closely resemble the optimal results achieved with the protocol used for the majority of the previous work (33). For example, an orientation-dependent desolvation term that has been described (33, 63) is included in the energy function used in this work, whereas it was previously not available in trunk Rosetta.

Calculations were carried out using the structures 2qoj and chain A of 3eh8 as starting scaffolds. Residues in a 6 Å region surrounding the target base-pair were allowed to design. As in past work, if the variant amino acid was in the top three amino acids by frequency in the set of 56 repeated design runs, then the position was considered successfully redesigned (33). For the few variants with loop length changes the predictions did not incorporate loop-modeling steps, and

instead the sequence of the region was aligned and only the relevant interface positions were allowed to design or were substituted to generate the model used in specificity prediction.

Energy function and command lines used for design and specificity prediction, all flags are described in detail in reference 33:

Energy function:

Weight set:

```
METHOD_WEIGHTS ref  -0.3 -0.7 -0.75 -0.51 0.95 -0.2 0.8 -0.7 -1.1 -0.65 -0.9
-0.8 -0.5 -0.6 -0.45 -0.9 -1.0 -0.7 2.3 1.1

special_rot 1.0
fa_atr 0.95
fa_rep 0.44
fa_sol 0.65
fa_intra_rep 0.004
hack_elec 0.5
fa_plane 0
fa_dun 0.56
ref 1
hbond_lr_bb 1.17
hbond_sr_bb 1.17
hbond_bb_sc 1.17
hbond_sc 1.17
lk_ball 0.325
lk_ball_iso -0.325
p_aa_pp 0.64
dslf_ss_dst 0.5
dslf_cs_ang 2
dslf_ss_dih 5
dslf_ca_dih 5
pro_close 1.0
```

To go with this optimized weight set, the atom_properties and lys.params files are modified exactly as described in the supplement of previous work on protein-DNA design (33). These modifications to the atom_properties (more important than lysine changes) are as follows:

```
Phos     P    2.1500    0.5850   -4.1000    3.5000   14.7000
Narg     N    1.7500    0.2384  -10.0000    6.0000   11.2000 DONOR ORBITALS
NH2O     N    1.7500    0.2384   -7.8000    3.5000   11.2000 DONOR ORBITALS
Nlys     N    1.7500    0.2384  -16.0000    6.0000   11.2000 DONOR
ONH2     O    1.5500    0.1591   -5.8500    3.5000   10.8000 ACCEPTOR SP2_HYBRID ORBITALS
```

Command line arguments used for design with motif_dna_packer_design application:

```
-dna::specificity::exclude_dna_dna false
-mute all
-run_motifs
-dtest 2.0
-z1 0.97
-r1 1.0
-z2 0.97
-r2 1.0
-motifs::rotlevel 8
-motifs::list_motifs /work/sthyme/list_August2011Motifs_noduplication_IAniI
-motifs::output_file ./XXXX.motifs
-packing::max_rotbump_energy 10.0
```

```
-patch_selectors SPECIAL_ROT
-probe_specificity 3
-binding
-score:output_residue_energies
-run:output_hbond_info
-run:min_type dfpmin_armijo
-run:min_tolerance 0.0001
-ndruns 1
-dna::design::z_cutoff 6.0
-score::weights /work/sthyme/weights/trunk_lkball.wts
-file:s /work/sthyme/INPUTPDBS_ANII/2QOJ.pdb
-dna::design::dna_defs X.408.ADE
-in:ignore_unrecognized_res
-database /work/sthyme/trunk_rosetta_2012/rosetta_database/
-ex1
-ex2
-ex1aro::level 6
-ex2aro::level 6
-exdna::level 4
-extrachi_cutoff 0
-jd2:dd_parser
-overwrite
```

Command line arguments used for modeling fixed sequences and calculating energies for specificity prediction with the rosettaDNA application:

```
-dna::specificity::exclude_dna_dna false
-mute all
-packing::max_rotbump_energy 10.0
-dna:design:repack_only true
-patch_selectors SPECIAL_ROT
-score:output_residue_energies
-run:output_hbond_info
-run:min_type dfpmin_armijo
-run:min_tolerance 0.0001
-ndruns 1
-dna::design::z_cutoff 6.0
-score::weights /work/sthyme/weights/trunk_lkball.wts
-file:s /work/sthyme/INPUTPDBS_ANII/2QOJ.pdb
-dna::design::dna_defs X.408.ADE
-in:ignore_unrecognized_res
-database /work/sthyme/trunk_rosetta_2012/rosetta_database/
-ex1
-ex2
-ex3
-ex4
-ex1aro::level 6
-ex2aro::level 6
-exdna::level 4
-extrachi_cutoff 0
-jd2:dd_parser
-parser:protocol ../xml
-overwrite
```

Corresponding XML file for using the rosettaDNA application:

```
<dock_design>
  <TASKOPERATIONS>
    <InitializeFromCommandline name=IFC/>
    <IncludeCurrent name=IC/>
    <RestrictDesignToProteinDNAInterface name=DnaInt z_cutoff=6.0 dna_defs=X.408.ADE/>
    <OperateOnCertainResidues name=AUTOprot>
      <AddBehaviorRLT behavior=AUTO/>
      <ResidueHasProperty property=PROTEIN/>
    </OperateOnCertainResidues>
    <OperateOnCertainResidues name=ProtNoDes>
      <RestrictToRepackingRLT/>
      <ResidueHasProperty property=PROTEIN/>
    </OperateOnCertainResidues>
```

```
    <OperateOnCertainResidues name=DnaNoPack>
      <PreventRepackingRLT/>
      <ResidueHasProperty property=DNA/>
    </OperateOnCertainResidues>
  </TASKOPERATIONS>
  <SCOREFXNS>
    <LKB weights=trunk_lkball/>
  </SCOREFXNS>
  <FILTERS>
    <FalseFilter name=falsefilter/>
  </FILTERS>
  <MOVERS>
    <DesignProteinBackboneAroundDNA name=bb_lk scorefxn=LKB
task_operations=IFC,IC,AUTOprot,ProtNoDes,DnaInt type=ccd gapspan=4 spread=3
cycles_outer=3 cycles_inner=1 temp_initial=2 temp_final=0.6/>
    <DnaInterfacePacker name=DnaPack scorefxn=LKB
task_operations=IFC,IC,AUTOprot,ProtNoDes,DnaInt probe_specificity=1 binding=1/>
  </MOVERS>
  <PROTOCOLS>
    <Add mover_name=DnaPack/>
  </PROTOCOLS>
</dock_design>
```

When predicting the specificity of variants, the sequence of the starting scaffold PDB was changed with a script and the exact same protocol was used as for specificity prediction with the wild-type endonuclease sequence. Scripts for are available upon request for generating these variant PDBs, setting up organized directories for large-scale specificity prediction of many variants, and analyzing both design and specificity prediction data. The majority of the arguments in the command lines for both protocols are described in more detail in the supplementary material of the publication that details the motif-based design method (33).

Example PSSM file used for I-AniI target site searches:
Colors: Wild-Type, Tolerated Substitution, Engineered Variant Substitution
Values used are based on specificity data for I-AniI (12)

```
key A C G T
-10  2 1 2 -1
-9   100 50 -1 100
-8   -1 50 1 1
-7   30 100 -1 100
-6   100 1 -1 2
-5   -1 1 1 1
-4   100 2 -1 2
-3   1 1 -1 3
-2   1 1 1 -1
-1   1000 20 10 -1
1    1 1 1 -1
2    1000 -1 1000 10
3    2 2 2 -1
4    2 -1 2 2
5    1 1 1 -1
6    30 1 -1 50
7    2 4 30 -1
8    -1 1 1 1
9    -1 2 1 1
10   -1 1 1 1
```

Supplementary Figures

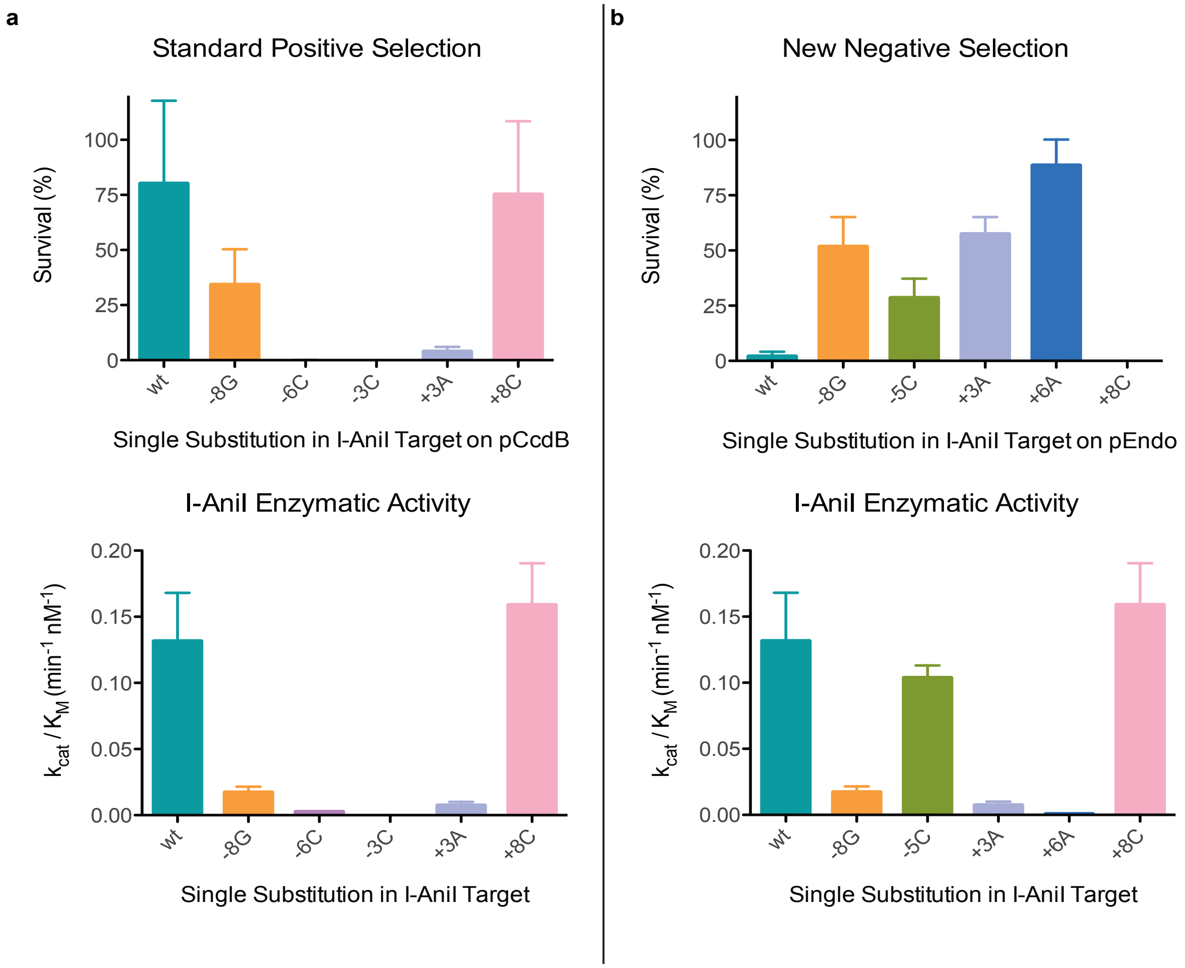

Figure S1. a) A comparison between survivals in the standard bacterial selection system (19) and kinetic data (12) for several single base-pair substitutions in the I-AniI target site. In this experiment, the target site is on the pCcdB plasmid and the M5 variant (32) of I-AniI is on the pEndo. The M5 or M4 variants (Figure 1) were used for all selection experiments in this work because they survive significantly better against single or double (tandem, not on opposite sides of the plasmid) wild-type target sites than the Y2 variant (9% for Y2 with two sites versus 44% with M5, completed side-by-side). High survival indicates cutting of the pCcdB plasmid and should correlate with a high k_{cat}/K_M. The selection system displays a wide dynamic range and survival closely matches the kinetic data for these single base-pair substitutions. **b)** A comparison between survivals with the new negative selection component of the bacterial selection system and kinetic data for several single base-pair substitutions in the I-AniI target site. In this experiment, the varying target site is on the pEndo plasmid and the pCcdB plasmid contains the wild-type target site. High levels of survival indicate that the M5 enzyme does not cleave the target site on the pEndo plasmid and that the pCcdB plasmid with the wild-type site is cleaved. This modified selection system also displays a wide dynamic range and the survival negatively correlates with the kinetic data.

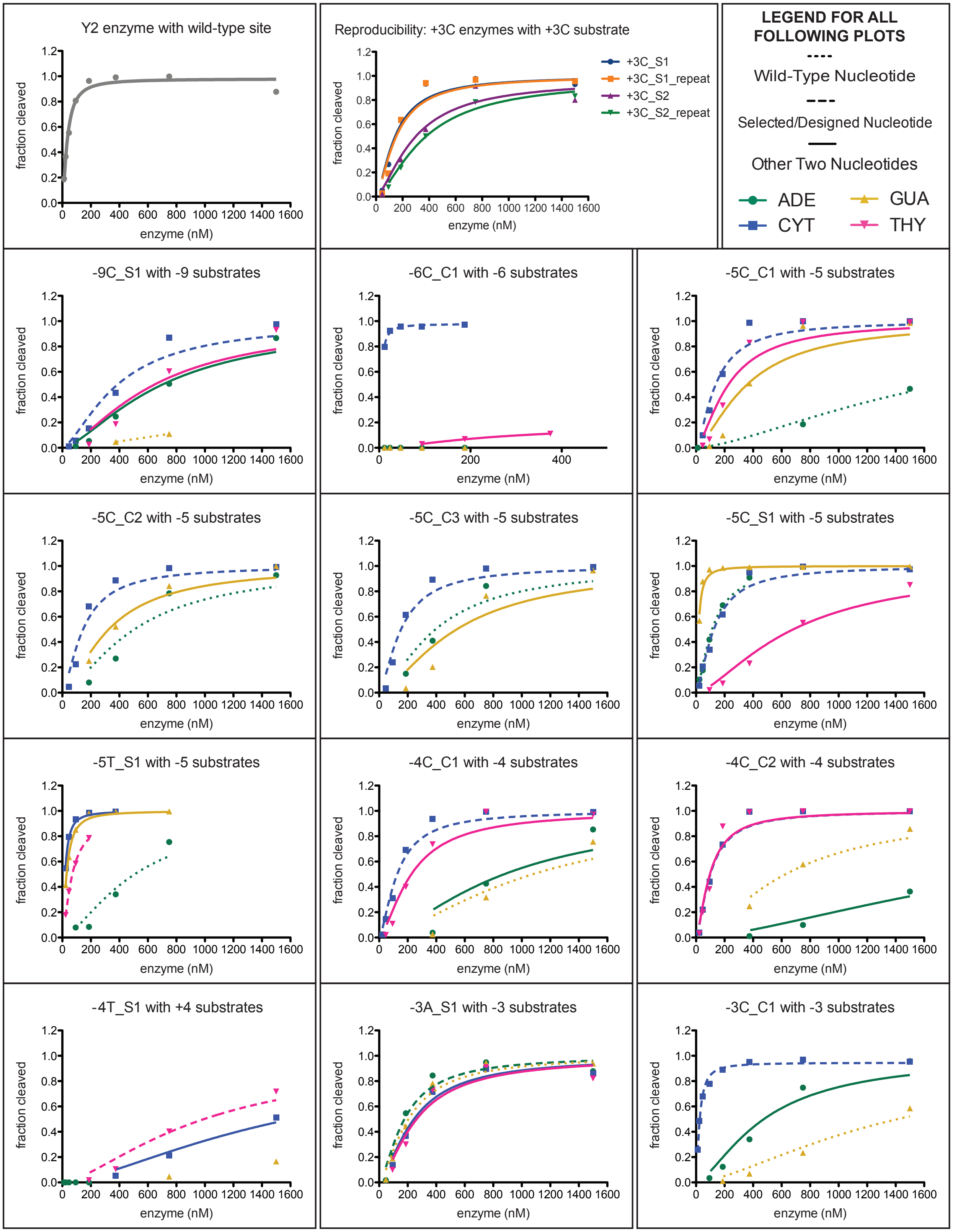

Y2 enzyme with wild-type site
Reproducibility: +3C enzymes with +3C substrate
+3C_S1
+3C_S1_repeat
+3C_S2
+3C_S2_repeat
LEGEND FOR ALL FOLLOWING PLOTS
Wild-Type Nucleotide
Selected/Designed Nucleotide
Other Two Nucleotides
ADE
GUA
CYT
THY
-9C_S1 with -9 substrates
-6C_C1 with -6 substrates
-5C_C1 with -5 substrates
-5C_C2 with -5 substrates
-5C_C3 with -5 substrates
-5C_S1 with -5 substrates
-5T_S1 with -5 substrates
-4C_C1 with -4 substrates
-4C_C2 with -4 substrates
-4T_S1 with +4 substrates
-3A_S1 with -3 substrates
-3C_C1 with -3 substrates
fraction cleaved
enzyme (nM)

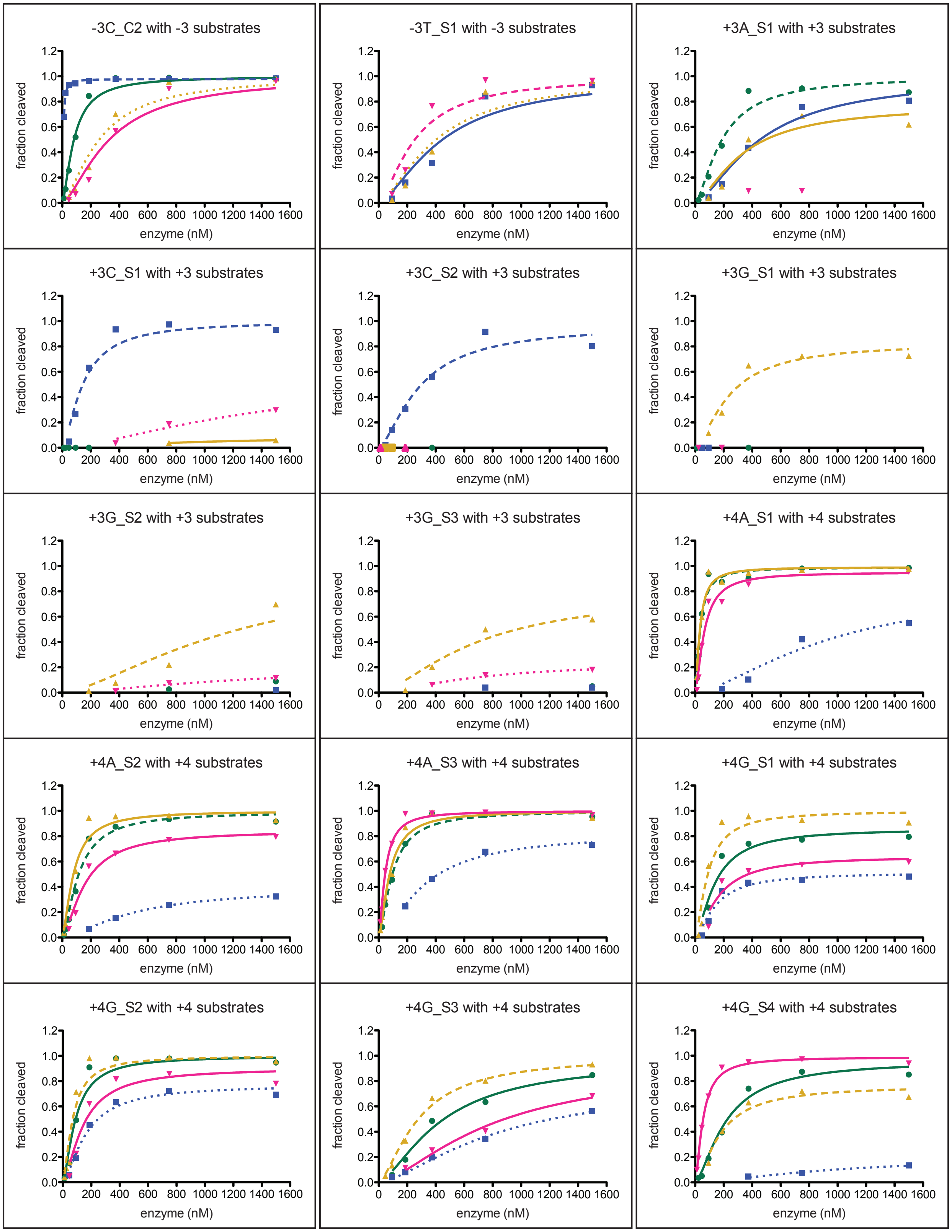
-3C_C2 with -3 substrates
-3T_S1 with -3 substrates
+3A_S1 with +3 substrates
+3C_S1 with +3 substrates
+3C_S2 with +3 substrates
+3G_S1 with +3 substrates
+3G_S2 with +3 substrates
+3G_S3 with +3 substrates
+4A_S1 with +4 substrates
+4A_S2 with +4 substrates
+4A_S3 with +4 substrates
+4G_S1 with +4 substrates
+4G_S2 with +4 substrates
+4G_S3 with +4 substrates
+4G_S4 with +4 substrates
fraction cleaved
0.0 0.2 0.4 0.6 0.8 1.0 1.2
enzyme (nM)
0 200 400 600 800 1000 1200 1400 1600

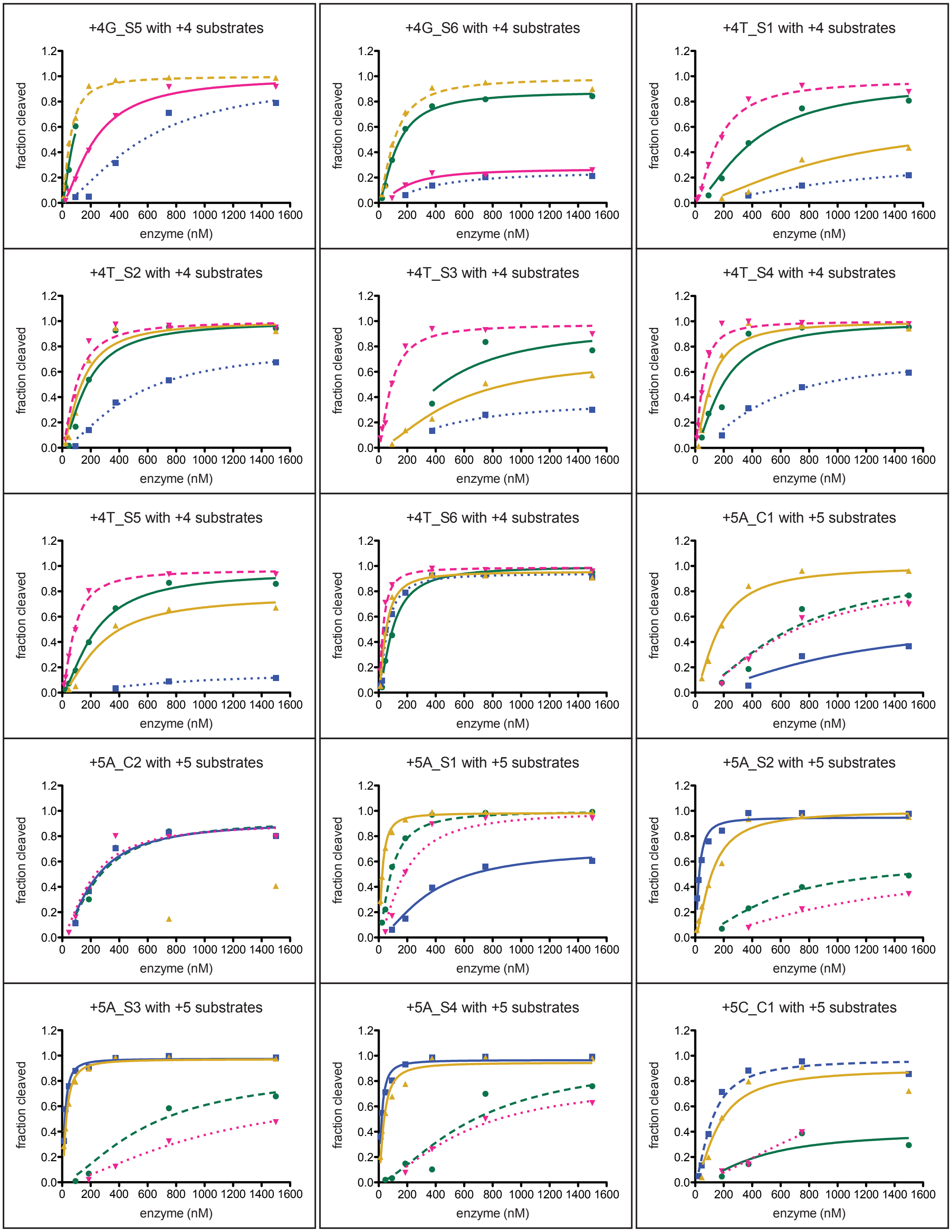
+4G_S5 with +4 substrates
+4G_S6 with +4 substrates
+4T_S1 with +4 substrates
+4T_S2 with +4 substrates
+4T_S3 with +4 substrates
+4T_S4 with +4 substrates
+4T_S5 with +4 substrates
+4T_S6 with +4 substrates
+5A_C1 with +5 substrates
+5A_C2 with +5 substrates
+5A_S1 with +5 substrates
+5A_S2 with +5 substrates
+5A_S3 with +5 substrates
+5A_S4 with +5 substrates
+5C_C1 with +5 substrates
fraction cleaved
0.0
0.2
0.4
0.6
0.8
1.0
1.2
enzyme (nM)
0
200
400
600
800
1000
1200
1400
1600

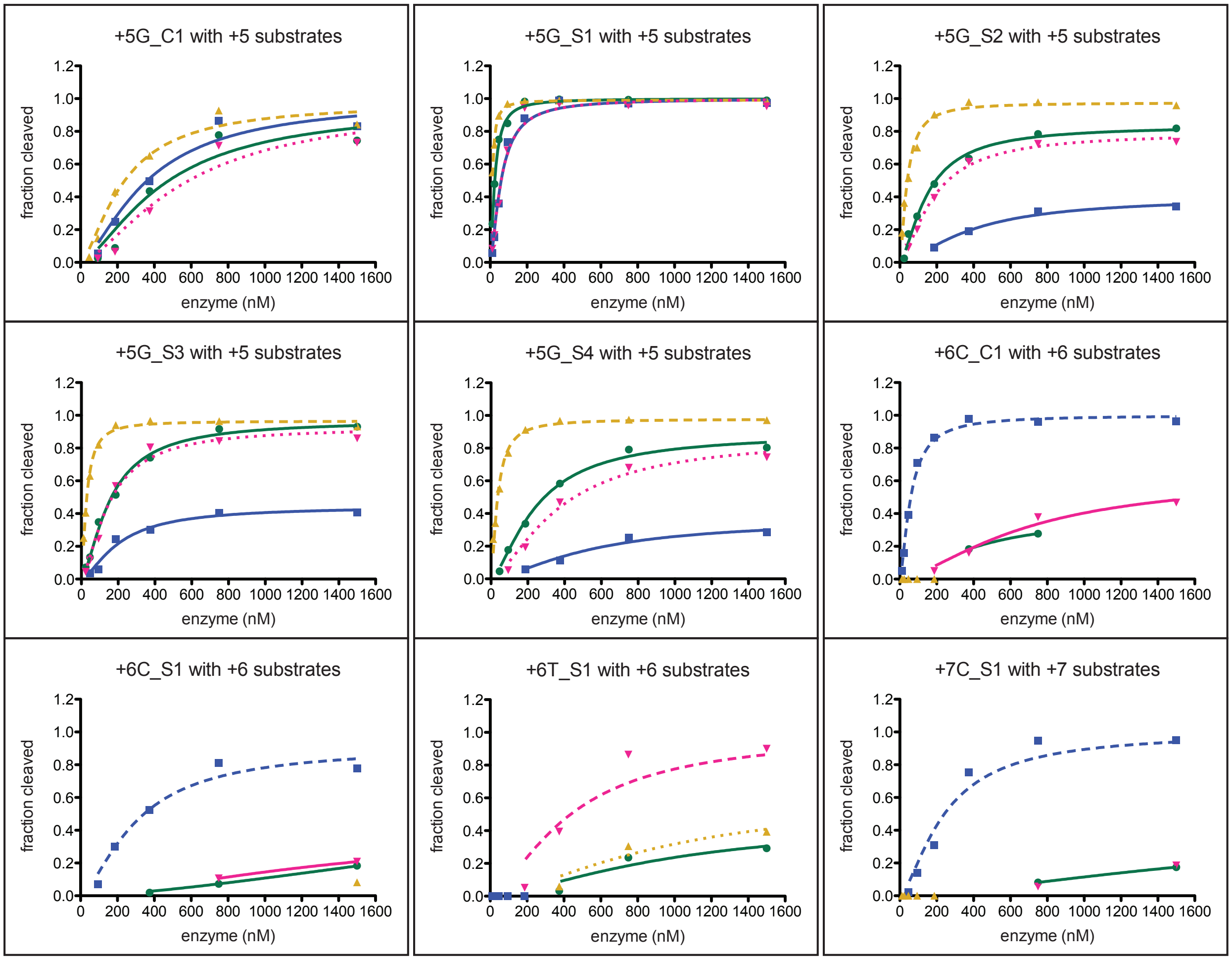

Figure S2. Cleavage data for selected endonucleases with each of the four bases at the targeted position. The activity level of the Y2 variant (32) of I-AniI with the wild-type target is shown in the first panel for comparison with these engineered enzymes. The second panel shows the high level of reproducibility for these cleavage experiments. The selection experiments all included a negative selection component against the wild-type I-AniI site. Thus it is expected that these enzymes will show high levels of cleavage of the targeted single base-pair substitution and reduced levels of wild-type site cleavage. This data is summarized in Table S1 as $EC_{1/2max}$ and specificity values.

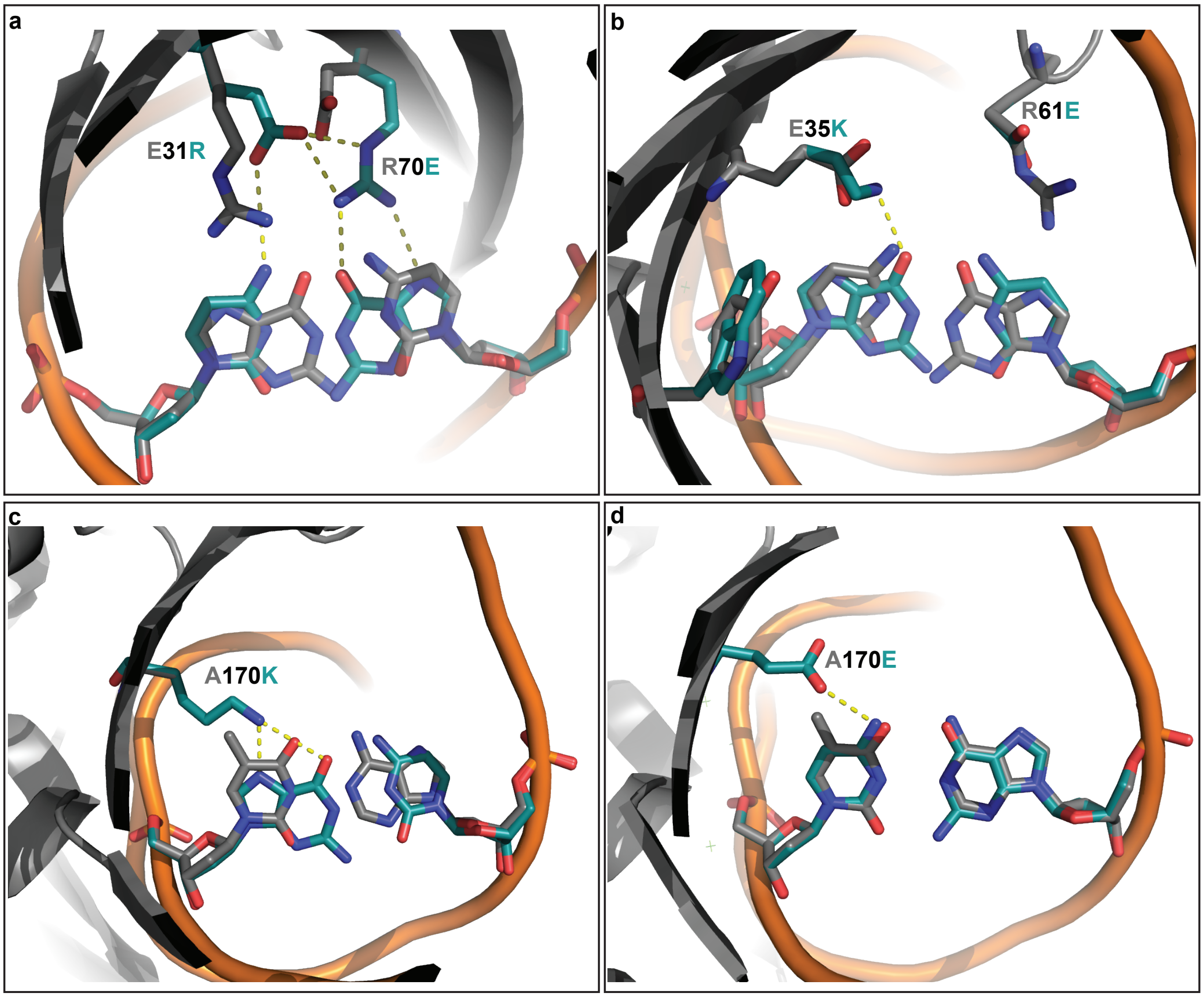

Figure S3. Computational models of motif interactions for single base-pair substitutions in the I-AniI (2qoj) target site show that the most specific I-AniI variants contain at least one strong, direct hydrogen-bonding contact. The presence of these contacts was the inspiration for the development of motif-biased design procedures (33), which enrich for native-like protein-DNA interactions. Each of the residues shown in the structures in this figure is classified as a motif contact by the computational methods. The lynchpin residue in the -6C and -3C variants was identified by previous computational methods (12), and libraries that combined the computational information with randomization of the surrounding residues increased both the activity and specificity of these two enzymes. The +3C and +3G variants both came from screens of fully randomized libraries using the modified directed evolution system that selects for specificity. **a)** The highly specific -6C_C1 variant contains two contacts, the computationally derived E31R and the R70E mutation that was identified by a selection with E31R fixed. **b)** The -3C_C1 and -3C_C2 variants contain the designed mutation E35K, and selection was used to alter the sequence and length of a neighboring loop for significantly enhanced activity and specificity. The selected loop sequences contain multiple potential strong contacts, included R61E and R61D (Table S1). **c)** The +3G_S1 variant contains A170K and was identified from a library of four fully randomized amino acids. **d)** The +3C_S1 variant contains A170E and was identified from a library of four fully randomized amino acids.

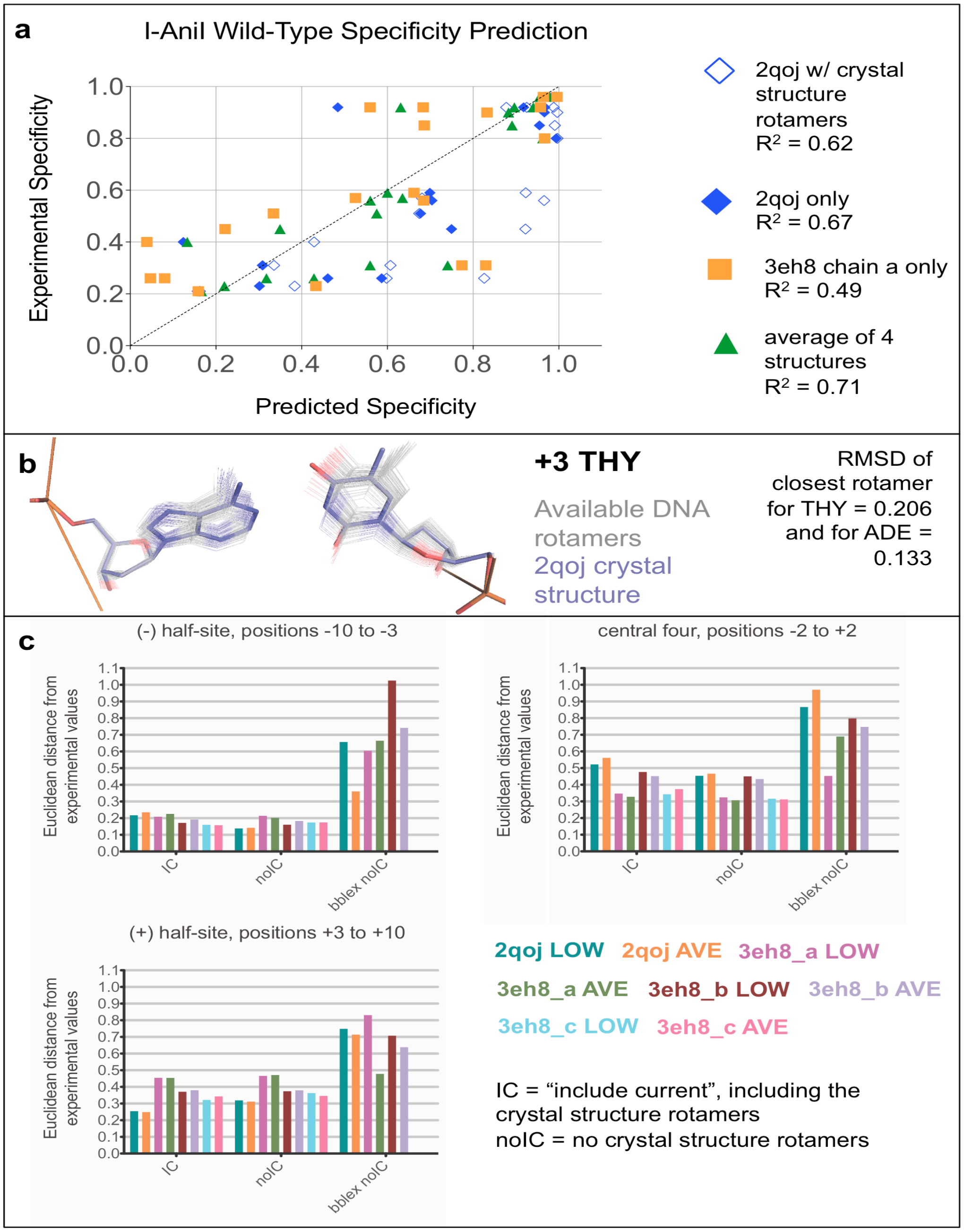

Figure S4. a) Fixed backbone calculations of specificity for each wild-type base in the I-AniI target site were compared to the experimentally-measured specificity. The calculated specificity was averaged across four starting structures (2qoj, and 3eh8, chains a, b, and c) and compared to the specificity predicted for the single 3eh8 structure (chain a) and the single 2qoj structure with and without the addition of crystal structure rotamers. The dashed black line represents y equals x, corresponding to perfect prediction. **b)** The crystal structure DNA nucleotide (blue sticks) at position +3T in the I-AniI target site is not included in the set of available DNA rotamers (gray lines). **c)** Comparison of fixed and flexible protein backbone predictions, where a lower Euclidean distance indicates that the prediction better matches the experimental data. Specificity calculations using the average over the 56 runs (ave) and also using the lowest energy structure of the 56 (low) are compared.

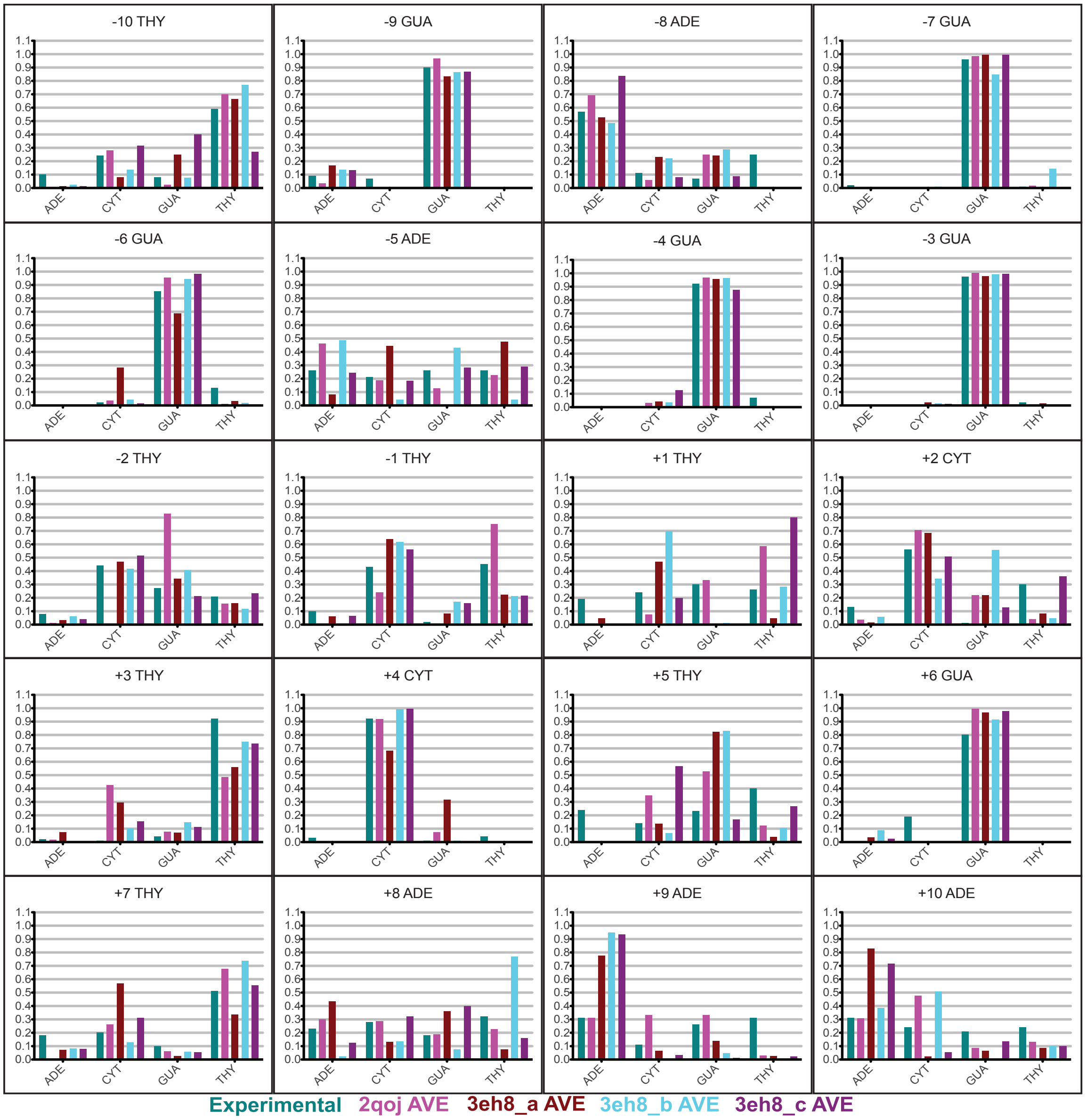

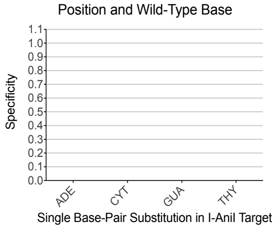

Figure S5. Specificity predictions compared with experimental specificity data (12) for each position in the I-AniI target site. The y-axis displays specificity, and low values mean the base-pair is poorly cut. Predictions were collected on four I-AniI structures. Highly specific positions, such as -4, were successfully predicted with all four structures. The most common cause of mis-predicted specificity is clashes that are not resolved with the fixed backbone model, such as in the case of +5A. Experimentally, +5A is similar to the other base-pairs at position +5. However, in the computational models the methyl group of the thymine paired with +5A clashes with the I-AniI protein in all four starting structures.

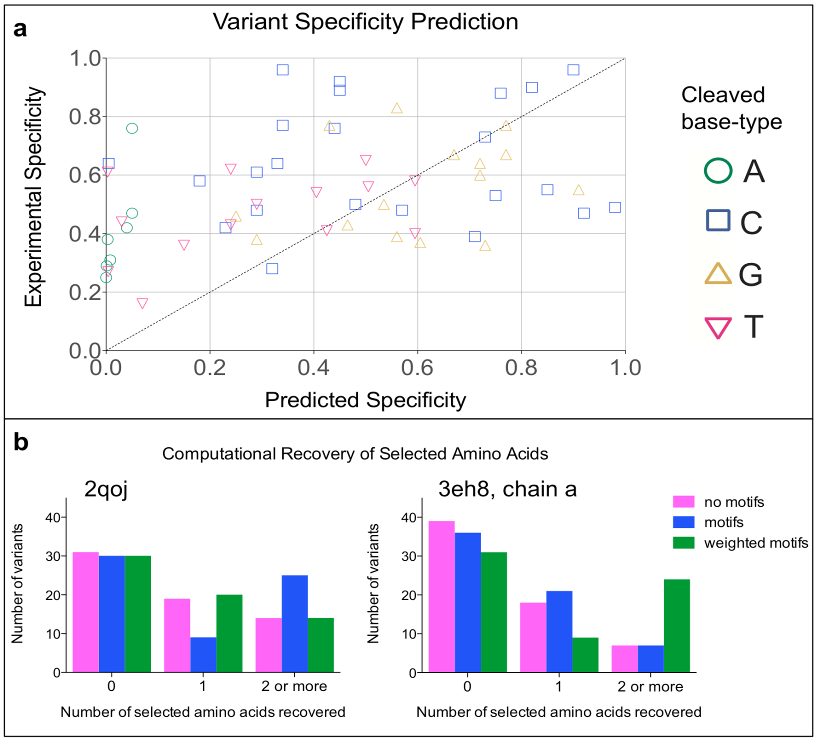

Figure S6. a) The variants are the same enzymes shown in Figure 3b and both color and shape correspond to the nucleotide type being targeted. The specificity predictions are averaged from the specificity values completed using two crystal structures, 2qoj and chain a of 3eh8 (separate values in Table S1). The dashed black line represents y equals x, corresponding to perfect prediction. **b)** Recovery of selected amino acids with computational design – without motif rotamers included in the design process, with motif rotamers, and with motif rotamers that have an energetic weight (-1.25 REUs) – using the crystal structures 2qoj and chain a of 3eh8.

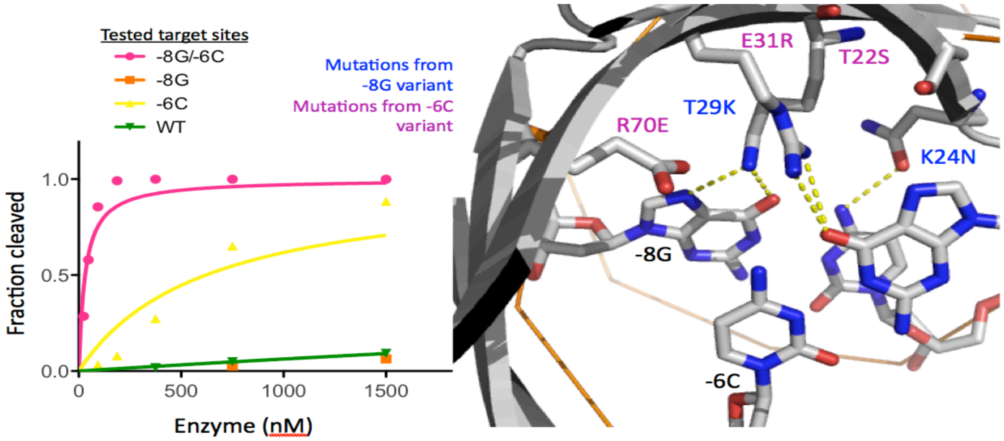

Figure S7. Two computationally derived endonuclease variants (12) can be combined without using selection for further sequence optimization. The -6C_C1 and -8G_P1 I-AniI variants are separated by only the -8G position. The main contact to -7G (R72) is maintained, maintaining the high specificity at -7 while successfully switching the specificity at -6 and -8. The variant with all five mutations targeting the two base-pair switches shows specific cleavage of the intended site.

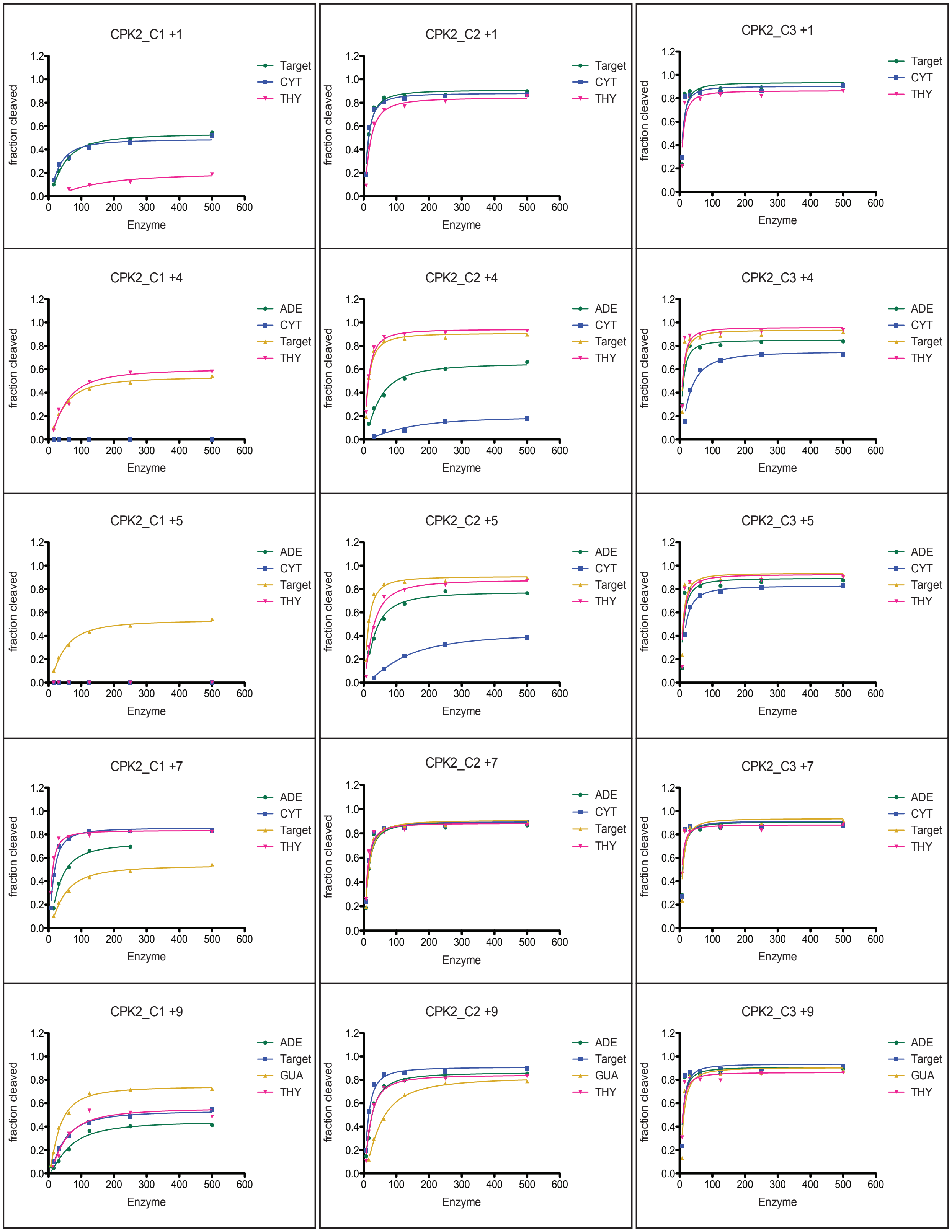

CPK2_C1 +1
Target
CYT
THY
CPK2_C2 +1
Target
CYT
THY
CPK2_C3 +1
Target
CYT
THY
CPK2_C1 +4
ADE
CYT
Target
THY
CPK2_C2 +4
ADE
CYT
Target
THY
CPK2_C3 +4
ADE
CYT
Target
THY
CPK2_C1 +5
ADE
CYT
Target
THY
CPK2_C2 +5
ADE
CYT
Target
THY
CPK2_C3 +5
ADE
CYT
Target
THY
CPK2_C1 +7
ADE
CYT
Target
THY
CPK2_C2 +7
ADE
CYT
Target
THY
CPK2_C3 +7
ADE
CYT
Target
THY
CPK2_C1 +9
ADE
Target
GUA
THY
CPK2_C2 +9
ADE
Target
GUA
THY
CPK2_C3 +9
ADE
Target
GUA
THY
fraction cleaved
0.0
0.2
0.4
0.6
0.8
1.0
1.2
Enzyme
0
100
200
300
400
500
600

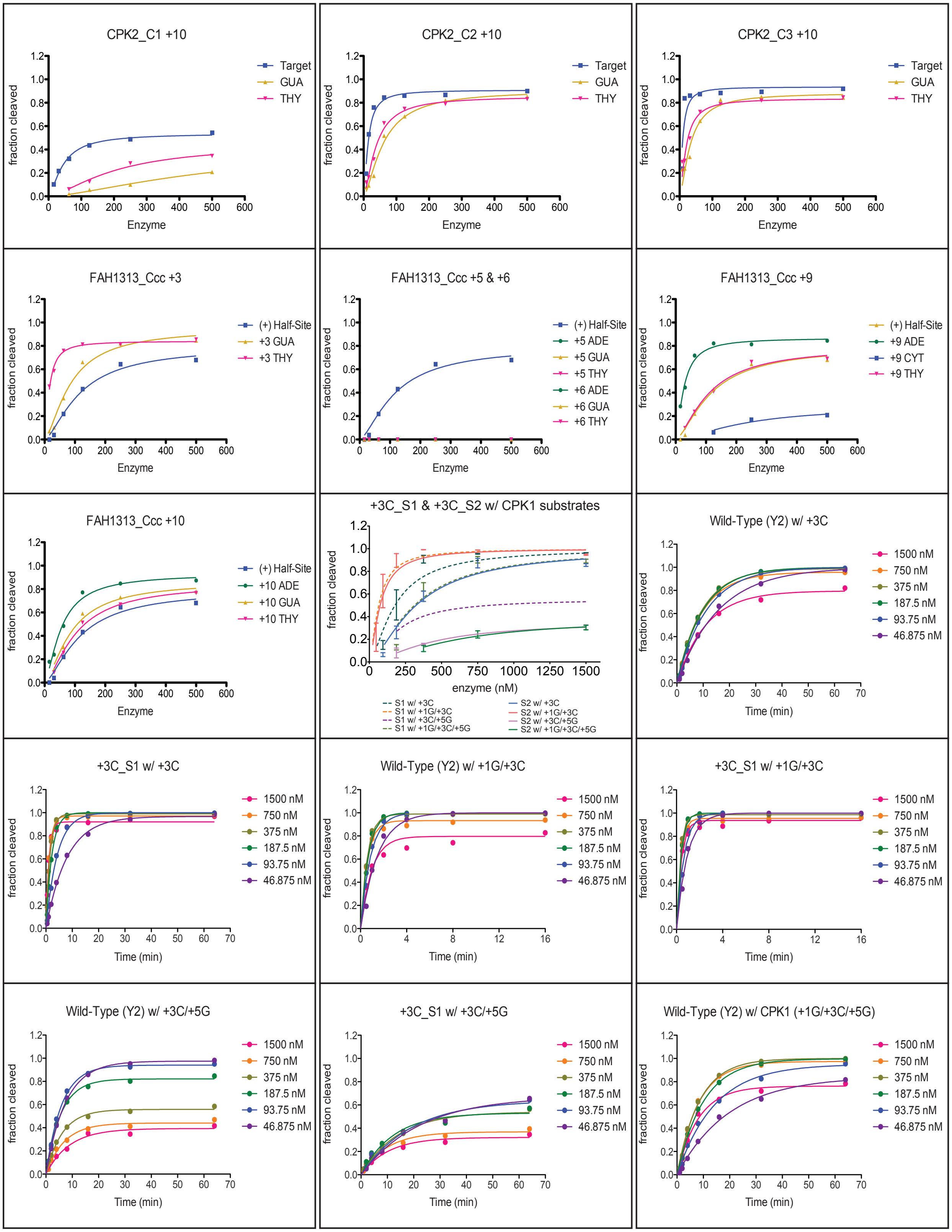

CPK2_C1 +10
CPK2_C2 +10
CPK2_C3 +10
fraction cleaved
Enzyme
Target
GUA
THY
FAH1313_Ccc +3
(+) Half-Site
+3 GUA
+3 THY
FAH1313_Ccc +5 & +6
+5 ADE
+5 GUA
+5 THY
+6 ADE
+6 GUA
+6 THY
FAH1313_Ccc +9
+9 ADE
+9 CYT
+9 THY
FAH1313_Ccc +10
+10 ADE
+10 GUA
+10 THY
+3C_S1 & +3C_S2 w/ CPK1 substrates
enzyme (nM)
S1 w/ +3C
S1 w/ +1G/+3C
S1 w/ +3C/+5G
S1 w/ +1G/+3C/+5G
S2 w/ +3C
S2 w/ +1G/+3C
S2 w/ +3C/+5G
S2 w/ +1G/+3C/+5G
Wild-Type (Y2) w/ +3C
+3C_S1 w/ +3C
Wild-Type (Y2) w/ +1G/+3C
+3C_S1 w/ +1G/+3C
Wild-Type (Y2) w/ +3C/+5G
+3C_S1 w/ +3C/+5G
Wild-Type (Y2) w/ CPK1 (+1G/+3C/+5G)
Time (min)
1500 nM
750 nM
375 nM
187.5 nM
93.75 nM
46.875 nM

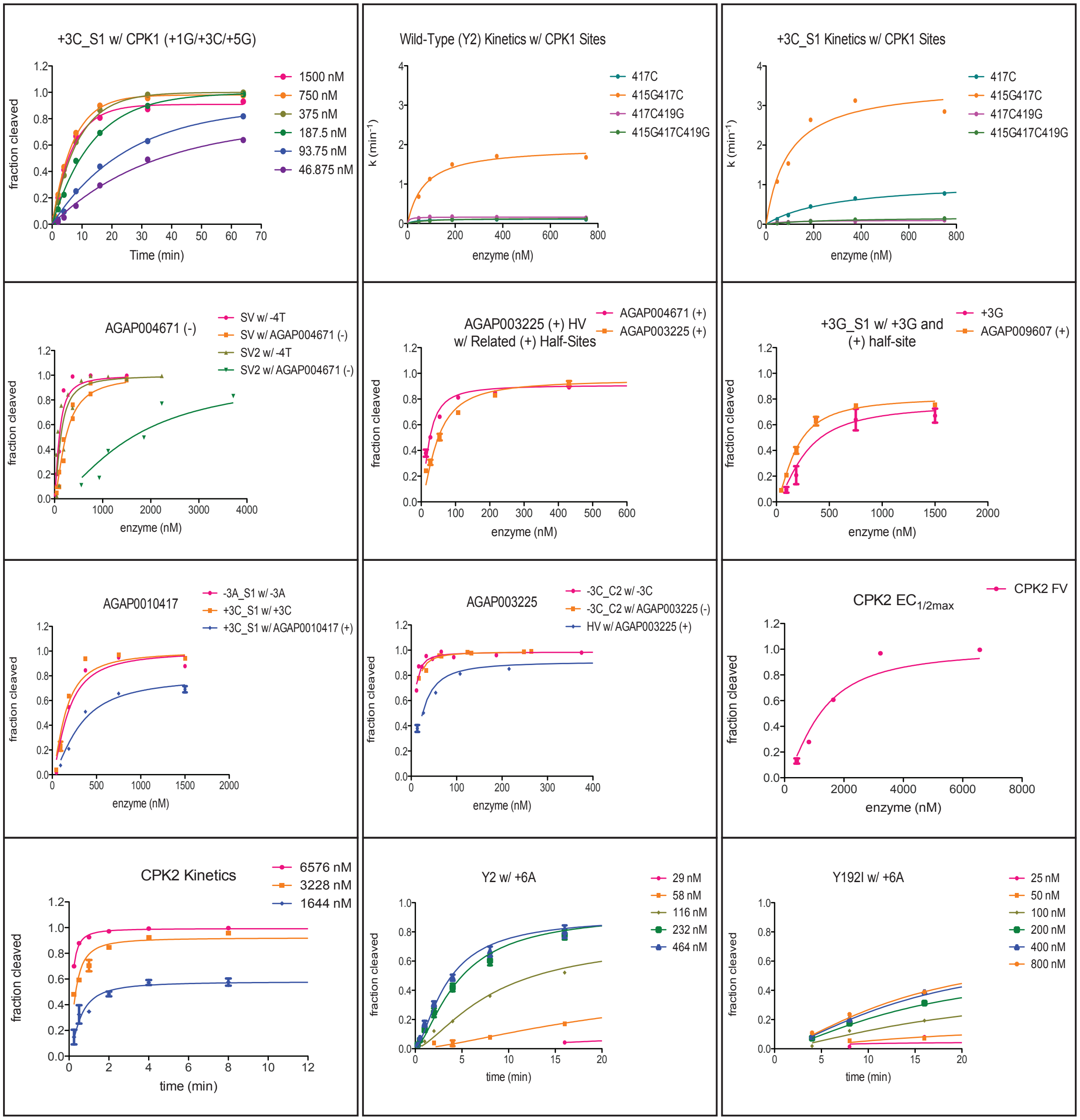

Figure S8. Cleavage data for endonucleases targeting multiple base-pair pockets and full sites.

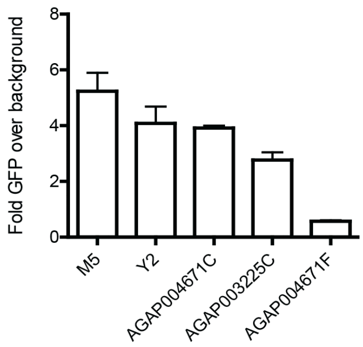

Figure S9. Endonucleases targeting the (+) half-site changes in two mosquito sites show activity in human cells. The level of cleavage of these two variants is comparable to cleavage by the M5 and Y2 variants of the I-AniI endonuclease (32) with the wild-type target site. An unsuccessful variant for the full AGAP004671 site, containing mutations known to abrogate cleavage on the site, is included to demonstrate the level of background in this assay. The two C-terminal variants were built on the M5 background. The assay was completed as previously described (42, 46).

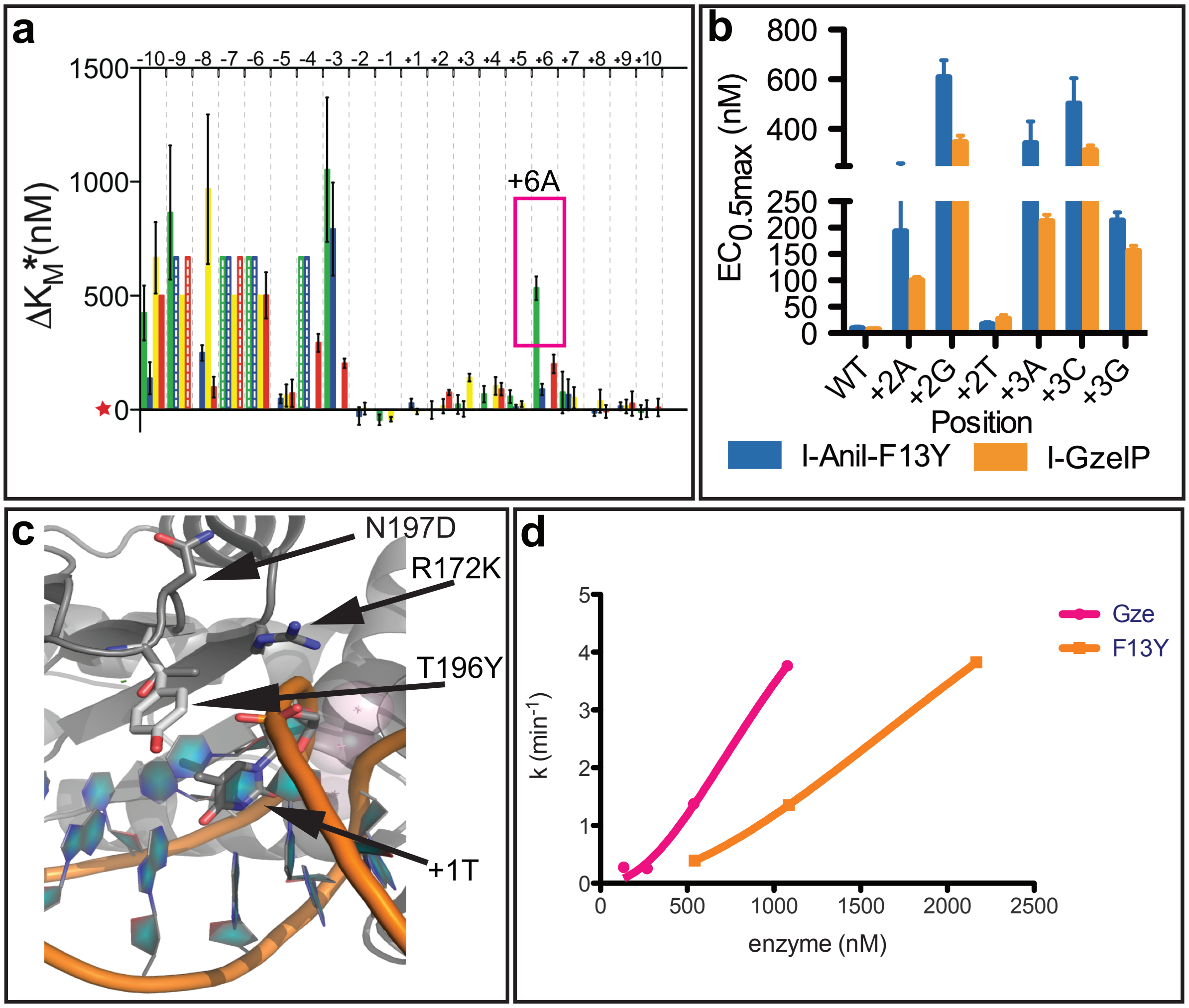

Figure S10. Evidence for a role of C-terminal loop 190-199 in formation of the ground-state reaction complex. **a)** Differences in binding affinity (K_M*) between I-AniI (Y2) with the wild-type I-AniI target and each possible single base-pair substitution (12). I-AniI has decreased binding affinity (increased K_M*) with +6A compared to the wild-type +6G. **b)** Activity ($EC_{1/2max}$) data for a hybrid enzyme built by transferring mutations at positions 172, 196, and 197 to the I-AniI scaffold (34). The enzyme is more active than the comparable I-AniI-F13Y starting scaffold (F13Y mutation only in base enzyme) with sites containing single base-pair substitutions near the transplanted mutations. **c)** Visualization of the mutations in this hybrid enzyme. T196Y may be increasing the hydrophobic interactions in the interface and be responsible for the better activity of the hybrid. **d)** Preliminary kinetic data for the hybrid variant compared to the I-AniI-F13Y starting scaffold with the same target site indicates that the improvement is likely due to increasing binding affinity (decreasing K_M*).

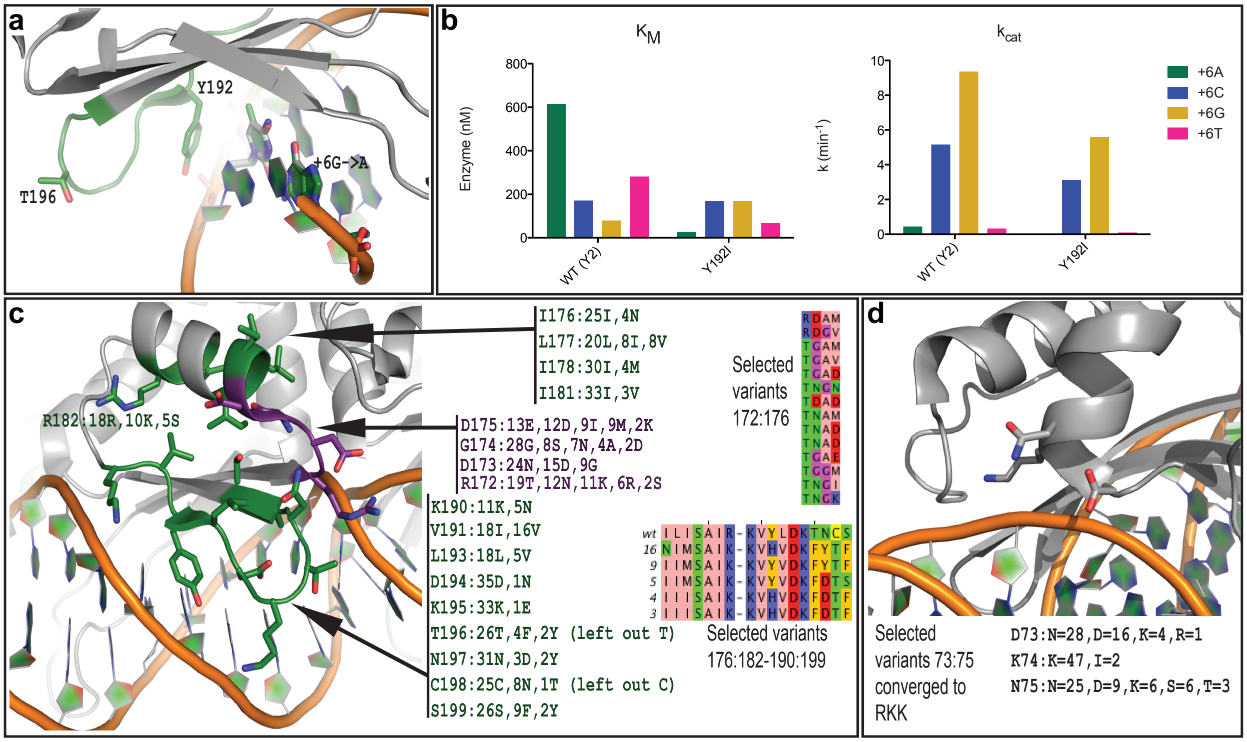

Figure S11. Mutations from I-AniI homologues that alter catalysis and binding. **a)** The loop containing Y192I and T196, another mutation implicated in ground state complex formation (Figure S10), is shown in green. The methyl group of the thymine on the DNA strand opposite to +6A may be destabilizing the loop and ground state complex. **b)** Kinetic analysis of the Y2 (WT) I-AniI endonuclease and a Y2-based variant with the Y192I mutation identified in homologous enzymes (34). Substitutions in the (+) half of the I-AniI target site mainly modulate k_{cat}* and not K_M*. The adenine substitution at position +6 is an exception, with a much higher K_M* than any other substitution in the (+) half-site (Figure S10). The Y192I substitution alters the binding of +6 substitutions, significantly lowering the K_M* for +6A. **c)** All homologues with over approximately 50% identity to I-AniI (34) were analyzed and the mutations in the loop from K190 to S199 and supporting residues from R172 to I181 were used to design limited libraries. The amino acid variation included in these libraries is shown on the structure. Not every mutation observed in the set of analyzed homologues was included in the libraries, and the frequencies that each type was seen are also shown (ie, D175:13E indicates that there were 13 glutamates seen at position 175). One library was built for the loop from R172 to D175 (purple), and a second was built with the remaining region spanning I176 to S199 (green). These libraries were screened against the -6C single base-pair substitution that significantly decreases binding affinity without altering catalysis. The sequences identified with the selection process are shown as alignments. **d)** Additional libraries were built from mutations identified in homologues in the N-terminal region from positions 73-75. This region is one of only a small number of regions that contact the DNA backbone and not the bases, making it a good candidate for modulation of non-specific interactions. The sequences included in the libraries are shown below the image of the area. The library was also screened against the -6C substitution and the sequence converged completely to D73R, K74K, and N75K.

Supplementary Discussion A. Rosetta computational predictions of experimental data.

The single base-pair specificity switches we describe in this paper provide a valuable benchmark for evaluating and guiding the improvement of computational design methods. Both specificity prediction and sequence design calculations were carried out for these I-AniI variants, as well as for all positions in the wild-type I-AniI interface, and compared to experimental data to assess the accuracy of the computational model. For the specificity calculations, the base in the crystal structure DNA targeted by the variant enzyme was substituted with each of the four possible nucleotides at the position and the energies of the four complexes were compared. The protein positions surrounding this position were kept fixed in sequence, either the sequence of the evolved variant or the wild-type protein sequence, for specificity calculations, but allowed to vary in design calculations.

We compared the experimentally determined specificity of I-AniI to specificity predicted for 1) four single I-AniI structures, 2) the average of the four, and 3) with and without inclusion of sidechain and base conformations from the starting crystal structure (Figure S4a, Figure S5). The predictions were most accurate for positions with high specificity and direct contacts, while there was significantly less agreement for positions with lower specificity. Supplementation of the standard rotamer libraries used to model sidechain (69) and base conformations (63) with conformations from the starting crystal structures caused an inaccurate bias for the crystal structure base-pair (Figure S4a). In calculations including DNA base conformations, the crystal structure nucleotide was almost always incorporated at one or both strands of the targeted position, resulting in inaccurately high specificity prediction. At the one target site position that benefited from inclusion of the crystal structure rotamer, none of the Rosetta-generated DNA rotamers was similar enough to produce the low energy interactions with the +3 base-pair that correspond to high specificity (Figure S4b). These results indicate that the DNA rotamers generated are not sufficient for accurate modeling of specificity, and it will be important to improve these rotamers for future work.

The average specificity value calculated over the four structures matched the experimental data better than the single structure, suggesting the protein backbone of the starting structure also biases this calculation (Figure S4a). The bias observed here can in principle be resolved by incorporating backbone flexibility (64–67) but this is complicated as the levels of structural movement must be enough to resolve clashes that erroneously penalize tolerated sequence changes, but not so much that the specificity signal is lost in the energetic noise of these movements or that inaccurate energetic minima are favored. Flexible backbone calculations performed much worse than did fixed backbone calculations for recapitulation of I-AniI wild-type specificity data (Figure S4c).

Calculations employing the wild-type I-AniI crystal structures were less successful in recapitulating the cleavage specificities (Figure S6a, Table S1) and amino acid sequences (Figure S6b) of the engineered variants, likely also because of changes in either the protein or DNA backbone positions not modeled in the calculations. The recently introduced motif-biased design approach (33), in which motif rotamers are incorporated into the Rosetta rotamer library and given an energy bonus, was more successful in recovering the sequences of the variants given their cleavage specificities than design without motifs. The majority of motif interactions are

direct, hydrogen-bonding contacts, and the model more accurately identifies these interactions than indirect contacts. However, even with the motifs, the recapitulation of this experimental benchmark was significantly less successful than in similar calculations recovering the sequences of crystal structures (33).

A key issue in computational modeling and design of new protein sequences starting from a static crystal structure is how to add enough flexibility to resolve small incompatibilities that are normally tolerated by dynamic proteins, but not allow so much that inaccurate minima are found for repulsive interactions that should not be tolerated. The minimal movement used in this work, only rotamers on both sides of the interface, was not enough to resolve clashes in some cases, particularly when attempted to recapitulate the specificity of engineered variants. Our finding that the different I-AniI structures can produce significantly different results (Figure 4a, Figure 5) indicates that combining information from multiple experimentally determined backbones would be more successful than using a single starting point. Multiple starting crystal structures are rarely available, so an algorithm that produces multiple experimentally relevant backbones based on the original electron density would be very useful (68). Since the repulsive interactions that need to be resolved are often arising between backbone and side-chain atoms, another potential option would be to eliminate the repulsive interaction energy term only between backbone side-chain atoms during fixed backbone modeling stages.

Supplementary Discussion B. Improving the binding affinity of I-AniI.

Selections to improve the overall activity of I-AniI (without changing the target sequence) have been successful in the past (32). The Y2 (S111Y, F13Y) and M5 (Y2 with I55V, F91I, S92T) variants of I-AniI that were used in all of the assays and libraries screened in this work were identified using the original version of the bacterial selection system that only screens for activity (19, 32). Before I-AniI was improved with these mutations, it showed essentially no survival in the bacterial system, even when four copies of its target site were included on pCcdB. The enzyme that was engineered to cleave the full CPK2 site has poor binding affinity compared to its wild-type precursor (Figure 6c). Selections to improve activity and binding for the wild-type endonuclease have the potential to identify mutations that can be incorporated into engineered enzymes like this one. The previously identified Y2 mutations, S111Y and F13Y, are both in the N-terminal domain that is associated with substrate binding and ground state complex formation (12). These findings indicated that the most promising regions for improving these same characteristics were other N-terminal areas that are near the DNA backbone, since the goal is non-specific affinity rather than direct base contacts. However, it was noticed I-AniI showed decreased binding affinity (K_M*) when the +6 position in its target sequence was mutated from guanine to adenine (Figure S10a). This result is surprising because DNA substitutions in the rest of (+) half of I-AniI interface do not affect K_M* or formation of the ground state complex (12). Therefore, it might be possible to modulate K_M* through mutations in the C- terminal domain of the complex. Further support for this insight came from examining a homologue-derived hybrid enzyme with mutations at positions 172, 196, and 197, all in the C-terminal domain (34). This variant showed increased activity over the wild-type I-AniI and preliminary kinetic data indicated the improvement was due to decreasing K_M* (Figure S10). A key feature of this enzyme was the mutation of T196 to a tyrosine, significantly increasing hydrophobic interaction between the protein and DNA on the (+) half of the target site (Figure S10c).

While it is unclear exactly how the +6A mutation decreases binding affinity, one potential result of the base substitution is an increase in ground state repulsive interactions between the protein and the methyl group of the thymine paired with +6A. This methyl group is near residue Y192, which is on the same loop as the T196 substitution in the homologue-derived variant with increased activity (Figure S10). Position Y192 is mutated to an isoleucine in homologous endonucleases with a +5A single base-pair substitution (34), which also places a methyl group near this protein position. To further explore the role of this C-terminal loop from position 190 to 199, kinetic analysis was completed with the Y192I variant and all four base-pairs at position +6. If the thymine methyl is disrupting the interaction of Y192 in the ground state complex, then the isoleucine mutant should not display the high K_M* seen for the tyrosine with the +6A substitution. While the pattern of k_{cat}* remained relatively similar for the Y192I endonuclease, albeit slightly reduced for all bases, the pattern of K_M* shifted dramatically. The Y192I mutation does result in significantly tighter binding of the +6A substitution, while affinity for the wild-type nucleotide remained relatively similar (Figure S11). This result provides evidence that a region of the I-AniI C-terminal domain is involved in interactions in the ground state complex, in contrast to the rest of the domain that was shown to play a role in forming the transition state complex.

Even though the previously identified I-AniI activating mutations (M5 and Y2) are all in the N-terminal domain, our results indicate that it is possible to improve binding affinity through mutations in one region of the C-terminal domain. After selection was used to identify the M5 and Y2, it was observed that some of these activating mutations were in I-AniI homologues. With the goal of further improving the binding affinity of I-AniI variants, homologues with over 50% identity to I-AniI were analyzed in the N-terminal domain and the C-terminal region that has been implicated in ground state complex formation. Three libraries were built and screened for activity using bacterial cells containing a site with the -6C substitution. This particular substitution is known to substantially reduce I-AniI binding affinity in enzyme assays (12) and the most active I-AniI variant (M5) was shown to have effectively no survival in these cells (Figure S1). Thus, variants with increased binding affinity greater than M5 will be selected from these libraries. The amino acids included in the starting libraries are shown in Figures S11c and S11d. The selected enzymes from the N-terminal library and the smaller C-terminal library (172:175) showed survivals of approximately 100% against the -6C cell line that previously was not cleaved by M5. The other C-terminal library also showed improved survival, albeit not as significantly as the smaller libraries. The sequences of these high-surviving variants are also shown in Figures S11c and S11d. The N-terminal library converged completely to positively charged amino acids. The C-terminal libraries did not converge to specific sequences, but they showed trends for particular positions such as the mutation of R172 to a threonine. While these particular mutations may not provide exactly what is needed to increase the binding affinity of the CPK2 variant, screening libraries based on homologous enzymes did significantly improve I-AniI (M5) activity against the -6C containing substrate.

Table S1. Data for all single base-pair I-AniI variants described in this paper. Cleavage data is from the graphs in Figure S2. Endonucleases are named according to the position and base-pair of intended cleavage and the method used to engineer their new specificities (see further description of naming system below and in Figure 1). The "_P" or "previously published" enzymes that have k_{cat}/K_M activity data (highlighted in green) are from Thyme, S. B. *et al.* **Nature** (2009) (12) and the other published enzymes are from Szeto, M. D. *et al.* **JBC** (2011) (34). Sequences of variants that were selected, but not expressed and tested with cleavage assays, are also included in this table.

Targeted Position in the I-AniI Site and Selected Variant Name	Starting Library, X = all 20 Aas (either *NNN* or *NNS*)	Library Survival (0 - 1), round 2	Target Site		$EC_{1/2max}$ (nM), or k_{cat}/K_M for some _P enzymes	Cleavage Plateau (f_{max}), NA for k_{cat}/K_M	Cleavage Specificity; see methods for calculation	Predicted Specificity Showing Data for 2qoj / 3eh8_a; see supplemental methods
**Selected variants, S = selected from random library, C = selected from computationally guided library, P = previously published**								
-9C_S1, K23R, K24A, T29I	K23X, K24X, G25X, T29X	0.17	Target		388 ± 119	~1	0.42	0.31/0.31
			-9C	A	703 ± 231	~1	0.23	0.17/0.32
				G	1482 ± ?	0.41	0.11	0.38/0.37
				T	648 ± 412	~1	0.25	0.14/4E-2
Untested -9C variants, same library as -9C_S1	residue numbers in alignment: 23, 24, 25, 29		wt KKGT<br>3of5 RAGI<br>1of5 RCGC<br>1of5 RAGV					
Untested -9T variants	K23X, K24X, G25X, T29X	0.04	23, 24, 25, 29		wt KKGT<br>7of15 RAGT<br>2of15 RAGV<br>1of15 RAGQ	1of15 RAGI<br>1of15 KAGV<br>1of15 SAGQ<br>1of15 GAGT	1of15 RCGQ	
Untested -8C variants	K23X, K24H, G25X, T29X, L112X	0.34	23, 24, 25, 29, 112		wt KKGTL<br>2of8 RHHNL<br>1of8 RHGTL<br>1of8 RHSTA	1of8 SHGNA<br>1of8 GHGGA<br>1of8 AHGGA<br>1of8 RHRQA		
-8G_P1, K24N, T29K	NA, test of the method	0.50 for single clone test	Target		31 ± 5	0.89	0.92	0.82/0.02
			-8G	A	1107 ± 195	~1	0.03	0.11/2E-3
				C	1218 ± 333	0.91	0.02	0.07/0.94
				T	1019 ± 107	0.96	0.03	2E-6/0.03
Untested -8G variants, used as a control for selection method	K24X, T29X, E31X, R72X	0.35	24, 29, 31, 72		wt KTER<br>5of17 SHER<br>3of17 NRER<br>2of17 NQER	1of17 NKER<br>1of17 NTER<br>1of17 NKCS<br>1of17 NKTQ	1of17 NQTR<br>1of17 NTTR<br>1of17 NNER	
-8G_P2 K24N, L28V, T29K	NA	NA	Target		24 ± 3	0.94	0.92	0.81/0.03
			-8G	A	692 ± 370	0.69	0.03	0.11/2E-3
				C	956 ± 300	~1	0.02	0.08/0.94
				T	750 ± 170	~1	0.03	9E-7/0.04
-8T_P1, K24N, T29Q	NA, $EC_{1/2max}$ collected for -8T only = 20 nM, Thyme *et al*, 2009	NA	Target		5.3E-2 ± 2E-2	NA	0.61	1E-6/7E-3
			-8T	A	1E-3 ± 9E-5	NA	0.01	0.25/2E-4
				C	2E-3 ± 2E-4	NA	0.02	0.10/0.99
				G	3.1E-2 ± 9E-3	NA	0.36	0.65/4E-3
-6C_P1, T29S, E31R, R70L	NA	<<0.0001 for single clone	Target		2.7E-2 ± 3E-3	NA	0.92	0.75/0.62
			-6C	A	4E-4 ± 8E-5	NA	0.01	6E-7/3E-6
				G	1E-3 ± 8E-5	NA	0.03	0.20/0.36
				T	1E-3 ± 2E-6	NA	0.03	0.05/0.03
-6C_C1, T22S, E31R, R70E	S20X, T22X, E31X/R (2 libs), S57X, R70X	0.46/0.15 (round 2), 0.34/0.97 (round 3)	Target		4 ± 0.3	0.98	0.96	0.62/0.19
			-6C	A	>260	-	0.02	7E-6/1E-4
				G	>260	-	0.02	0.32/0.72
				T	260 ± 25	0.18	0.02	0.06/0.09
Untested -6C variants, same libraries as -6C_C1	22, 31, 70<br>wt TER<br>15of20 SRE<br>2of20 SRT<br>1of20 ATT	1of20 GTT<br>1of20 TLT						
-6T_P1, A68Y, L69F, R70K, I71V	NA	NA	Target		135 ± 18	0.98	0.16	0.10/0.05
			-6T	A	176 ± 43	0.31	0.12	4E-8/1E-6
				C	>750	-	0.03	0.22/0.34
				G	31 ± 11	~1	0.69	0.68/0.62

-6T_P2, P1 mutations + 57-65 = GFRKRTLV +SGVVS+ K	NA	NA	Target		28 ± 4	0.99	0.44	6E-3/0.06
			-6T	A	208 ± 45	1	0.06	1E-8/2E-6
				C	112 ± 21	1	0.11	0.03/0.31
				G	32 ± 3	0.96	0.39	0.97/0.31
-5C_C1, Y18C, S20K, G33S	Y18X, S20K, T22X, G33X, E31X	0.62	Target		138 ± 33	~1	0.48	0.43/0.56
			-5C	A	1752 ± 1091	~1	0.04	9E-4/3E-11
				G	359 ± 183	~1	0.19	0.05/4E-5
				T	227 ± 79	~1	0.29	0.51/0.44
-5C_C2, Y18S, S20M, G33R	Y18X, S20X, G33R, E31X, A68X	0.62	Target		146 ± 35	~1	0.39	0.73/0.98
			-5C	A	505 ± 282	~1	0.11	0.03/4E-8
				G	326 ± 87	~1	0.17	0.17/3E-5
				T	~170	-	0.33	0.08/0.01
-5C_C3, Y18S, S20L, G33R, A68M	Y18X, S20X, G33R, E31X, A68X	0.62	Target		153 ± 33	~1	0.49	0.99/0.99
			-5C	A	395 ± 175	~1	0.19	6E-4/7E-7
				G	543 ± 400	~1	0.14	2E-3/4E-8
				T	~395	-	0.19	8E-3/1E-3
-5C_S1, Y18S, S20M, G33S, S57Y, A68C	Y18X, S20X, G33X, S57X, 59X, A68X	0.90	Target		125 ± 18	~1	0.10	0.77/0.99
			-5C	A	113 ± 13	~1	0.11	0.09/3E-4
				G	17 ± 2	~1	0.77	0.07/2E-3
				T	682 ± 192	~1	0.02	0.07/9E-8
Untested -5C variants, same libraries as above 5C selections	18, 20, 31, 33, 57, 68	wt YSEGSA<br>2of3 SMESYC<br>1of3 CMEASA<br>9of26 YGERSS			6of26 SMERSA<br>4of26 SKDTSA<br>3of26 SLERSM<br>3of26 CKESSA	1of26 SSCRSE		
Untested -5G variants	Y18X, S20X, E31X/wt, G33X, S57X/wt, 59X, A68X/R	0.65	18, 20, 31, 33, 57, 59, 68		wt YSEGSA<br>2of6 AAENSR<br>1of6 SAEVCR<br>1of6 CLECSL	1of6 TNTESR<br>1of6 ANEVSR<br>1of6 SGCDSR<br>4of4 SAESSR	1of4 AQEASR<br>1of4 ASEISR<br>2of6 SLENSR<br>1of6 SAEASR	1of6 CVENTR<br>1of6 AMECHR<br>1of4 SSETSR<br>1of4 SHENSR
-5T_P1, Y18F, S20T, I21V, T29A, A68S	NA	NA	Target		39 ± 10	0.97	0.50	0.28/0.50
			-5T	A	200 ± 9	~1	0.10	0.35/0.04
				C	73 ± 15	0.94	0.27	0.17/0.46
				G	135 ± 4	~1	0.14	0.20/6E-4
-5T_S1, Y18C, S20M, G33S	Y18X, S20X, G33X, S57X, 59X, A68X	0.53	Target		64 ± 3	0.94	0.16	0.54/0.43
			-5T	A	507 ± 327	~1	0.02	0.01/6E-5
				C	20 ± 1	~1	0.50	0.38/0.57
				G	30 ± 2	~1	0.33	0.07/1E-3
-4C_C1, Y18C, G33K, R59T, A68R	Y18X, S20X, G33K, R59X, A68X	0.46	Target		100 ± 14	~1	0.47	0.75/0.97
			-4C	A	2433 ± 4431	~1	0.02	6E-3/4E-4
				G	627 ± 267	~1	0.07	0.25/0.03
				T	97 ± 22	~1	0.48	2E-8/7E-5
-4C_C2, Y18C, G33K, R59S	Y18X, S20X, G33K, R59X, A68X	0.46	Target		126 ± 20	~1	0.55	0.62/0.94
			-4C	A	876 ± 1130	~1	0.08	0.11/9E-3
				G	1078 ± 1434	~1	0.06	0.27/0.05
				T	226 ± 55	~1	0.31	5E-7/7E-5
Untested -4C variants, same library as -4C_C1 and C2	18, 20, 33, 35, 59, 68	wt YSGERA<br>7of26 CSKESA<br>6of26 TSKETR<br>4of26 CSKEAA			3of26 CSKESR<br>2of26 MSRESA<br>2of26 MSREQA<br>1of26 YGKESA	1of26 CSKESQ<br>3of4 FSGAER<br>1of4 FSRAER		
-4T_S1, Y18G, S20Y, G33S, R59A, A68R	Y18X, S20X, G33X, R59X, A68X	<0.01	Target		993 ± 488	~1	0.35	4E-8/4E-5
			-4T	A	>1605	NA	0.22	0.38/0.21
				C	1605 ± 1210	~1	0.22	0.57/0.45
				G	>1605	NA	0.22	0.05/0.34
-3A_S1, Y18W, E35A, 58-65 = LRQHDRG-	Y18W, E35K, 58X, 60X, 61*VAS*, 62-65 X or deleted	>1	Target		177 ± 38	~1	0.31	5E-6/0.01
			-3A	C	240 ± 46	0.99	0.23	0.02/0.25
				G	205 ± 34	~1	0.26	0.96/0.53
				T	259 ± 79	0.99	0.21	0.02/0.20
Untested -3A variants, same library as -3A_S1	18, 35, 58-66	wt YEFRKRNEIEM<br>6 WALRQHGDRGM<br>3 WSYRPKLE--M<br>1 WAFRKEPQP-M			1 WGERTDTQ--M<br>1 WAVRPNNG--M<br>1 WAVRAETN--M<br>1 WAHRHHSTSTM	1 WSYRRHTN--M<br>1 WCMRVDTS--M		
-3C_P1, Y18W, E35K, R61Q	NA	0	Target		1.2E-2 ± 1E-4	NA	0.76	0.75/0.85
			-3C	A	2E-3 ± 8E-5	NA	0.13	1E-5/2E-3
				G	1E-3 ± 3E-5	NA	0.06	0.24/0.07
				T	7E-4 ± 7E-5	NA	0.05	0.02/0.09

-3C_C1, Y18W, E35K, (58-65) = YREQDG--	Y18W, E35K, 58X, 60X, 61*VAS*, 62-65 X or deleted	0.23 (round 3), 0.72 (round 4)	Target		24	0.95	0.90	0.83/0.83
			-3C	A	476	~1	0.05	6E-6/2E-3
				G	1413	~1	0.02	0.16/0.08
				T	>476	-	0.05	6E-3/0.09
-3C_C2, Y18W, E35K, (58-65) = FRPDG---	Y18W, E35K, 58X, 60X, 61*VAS*, 62-65 X or deleted	0.28 (round 3), 0.81 (round 4)	Target		7	0.98	0.88	0.48/0.87
			-3C	A	82	~1	0.08	3E-5/1E-3
				G	259	~1	0.02	0.51/0.05
				T	331	~1	0.02	0.01/0.08
Untested -3C variants, same libraries as -3C_C1 and _C2	58-66	WT FRKRNEIEM<br>3of10 FRPDG---M<br>1 IRPEG---M<br>1 VRKDG---M<br>1 FRNDG---M<br>1 FRKEG---M<br>1 FRPHG---M<br>1 FRSDG---M<br>1 MRPDN---M<br>2of13 YREQDG--M<br>1 TREKEG--M<br>1 IRDGGR--M<br>1 IRPHDG--M<br>1 FRPETN--M<br>1 YRPNEG--M<br>1 YRKNDG--M<br>1 YREHDA--M<br>1 VRSQEG--M<br>1 FRAHEG--M<br>1 ERRDDN--M<br>1 FRPEQG--M<br>1of2 FREDGGG-M<br>1 VRPDGNG-M<br>1of9 HRMEGGRGM<br>1 FRIERAGEM<br>1 ERLESGRWM<br>1 YRDEGRKGM<br>1 TRTEGARDM<br>1 QRGEGKHAM<br>1 VRGEGCRTM<br>1 YRSDNGRGM<br>1 YRWEGKRRM						
-3T_S1, Y18W, E35S, 58-65 = HRHDD---	Y18W, E35K, 58X, 60X, 61*VAS*, 62-65 X or deleted	0.25	Target		254 ± 96	~1	0.36	0.02/0.20
			-3T	A	>448	-	0.21	9E-6/0.02
				C	448 ± 179	~1	0.21	0.05/0.25
				G	404 ± 163	~1	0.23	0.93/0.54
Untested -3T variants, same library as -3T_S1	18, 35, 58-66	wt YEFRKRNEIEM<br>8of11 WSHRHDD---M<br>3of11 WAHRHDD---M						
+3A_S1, A170N, D194A, K200C	C150X, A170X, D194X, K200X	0.41	Target		189 ± 38	~1	0.42	0.03/0.13
			+3A	C	454 ± 98	~1	0.17	0.77/0.57
				G	331 ± 129	0.77	0.24	0.03/0.12
				T	>454	-	0.17	0.17/0.17
Untested +3A variants, same library as +3A_S1	170, 192, 194, 200	wt AYDK<br>9of15 LYAV<br>2of15 NYAC<br>2of15 CYAS<br>1of15 SYAV<br>1of15 CSRS						
+3C_P1, I164V, K200N	NA	NA	Target		14 ± 6	0.93	0.28	0.49/0.37
			+3C	A	146 ± 5	0.85	0.03	0.03/0.10
				G	155 ± 13	0.67	0.03	0.05/0.07
				T	6 ± 0.3	0.98	0.55	0.43/0.46
+3C_S1, A170E, D194K, K200A	C150X, A170X, D194X, K200X	0.59	Target		145 ± 33	~1	0.73	0.93/0.72
			+3C	A	>1407	-	0.08	0.03/0.14
				G	888 ± ?	0.09	0.12	0.04/0.14
				T	1407 ± 931	0.58	0.08	0.01/8E-5
+3C_S2, A170E, D194G, K200R	C150X, A170X, D194X, K200X	>1	Target		288 ± 68	0.97	0.64	0.66/0.10
			+3C	A	>1500	-	0.12	0.03/0.21
				G	>1500	-	0.12	0.12/0.68
				T	>1500	-	0.12	0.19/3E-3
Untested +3C variants, same library as +3C_S1 and S2	170, 192, 194, 200	wt AYDK<br>2of13 EYAV<br>2of13 EYAC<br>2of13 EYGR<br>2of13 WYGA<br>2of13 EYAV<br>1of13 EYSR<br>1of13 EYKA<br>1of13 TYCN<br>7of7 WRAV						
+3G_S1, A170K, Y192G, K200V	C150X, A170X, D194X, K200X	0.62	Target		240 ± 61	0.83	0.68	0.06/0.54
			+3G	A	>1500	-	0.11	0.02/0.43
				C	>1500	-	0.11	0.33/0.03
				T	>1500	-	0.11	0.58/1E-9
+3G_S2, A170V, D194S, K200R	C150X, A170X, D194X, K200X	0.40	Target		1245 ± 1173	~1	~0.25	0.28/0.45
			+3G	A	>1318	-	~0.25	0.03/0.07
				C	>1318	-	~0.25	0.40/0.07
				T	1318 ± 1100	0.21	~0.25	0.29/0.41
+3G_S3, A170V, D194G, K200R	C150X, A170X, D194X, K200X	0.82	Target		697 ± 296	0.80	~0.25	0.29/0.40
			+3G	A	>686	-	~0.25	0.02/0.07
				C	>686	-	~0.25	0.41/0.08
				T	686 ± 158	0.24	~0.25	0.28/0.45
Untested +3G variants, same library as +3G_S1-S3	170, 192, 194, 200	wt AYDK<br>8of22 NYAC<br>5of22 LWDR<br>3of22 KYAC<br>3of22 VYSR<br>2of22 KGDR<br>1of22 VYGR						
+4A_S1, C150S, D168G, I178V, K200Y	C150X, D168X, Y192X, D194X, K200X	0.53	Target		35 ± 5	0.99	0.38	4E-3/2E-3
			+4A	C	984 ± 475	0.87	0.01	0.41/0.08
				G	33 ± 4	0.99	0.40	0.26/0.05
				T	63 ± 9	0.95	0.21	0.32/0.87

+4A_S2, D168S, Y192V, D194M, K200I	D168X, Y192X, D194X, K200X	0.67	Target		115 ± 16	0.99	0.29	2E-3/9E-4
			+4A	C	492 ± 20	0.39	0.07	0.32/0.23
				G	79 ± 13	~1	0.43	0.58/0.34
				T	159 ± 28	0.85	0.21	0.10/0.43
+4A_S3, D168A, K190R, K200L	C150X, D168X, Y192X, D194X, K200X	0.53	Target		96 ± 10	~1	0.21	3E-3/1E-3
			+4A	C	316 ± 31	0.83	0.07	0.28/0.20
				G	77 ± 9	~1	0.27	0.58/0.44
				T	45 ± 4	~1	0.46	0.14/0.36
Untested +4A variants, same libraries as +4A_S1-S3	150, 168, 192, 194, 200	wt CDYDK<br>8of18 CNYDA<br>2of18 CNGAT<br>1of18 CSVMI	1of18 SGYDM<br>1of18 SGYDY<br>1of18 CAYDR<br>1of18 CAYDL		1of18 CAGDL<br>1of18 CNGCI<br>1of18 CSAYL			
+4G_S1, D168S, Y192G, D194G, K200Y	D168X, Y192X, D194X, K200X	0.31	Target		89 ± 23	~1	0.36	0.53/0.93
			+4G	A	140 ± 36	0.86	0.23	6E-3/2E-3
				C	147 ± 34	0.51	0.22	0.43/0.06
				T	161 ± 52	0.64	0.21	0.03/7E-3
+4G_S2, D168S, Y192V, D194G, K200V	D168X, Y192X, D194X, K200X	0.31	Target		69 ± 17	~1	0.38	0.11/0.47
			+4G	A	90 ± 18	~1	0.29	3E-3/2E-3
				C	165 ± 24	0.77	0.16	0.29/0.19
				T	144 ± 37	0.90	0.18	0.60/0.34
+4G_S3, D168G, Y192G, K200R	D168X, Y192X, D194X, K200X	0.31	Target		278 ± 26	~1	0.43	0.40/0.53
			+4G	A	440 ± 66	0.97	0.27	3E-3/2E-3
				C	837 ± 84	0.79	0.14	0.20/0.26
				T	797 ± 147	0.93	0.15	0.39/0.21
+4G_S4, D168A, Y192A, D194W, K200V	D168X, Y192X, D194X, K200X	0.28	Target		177 ± 35	0.76	0.19	2E-3/2E-3
			+4G	A	216 ± 29	0.96	0.16	0.02/0.01
				C	986 ± 282	0.20	0.03	0.71/0.77
				T	55 ± 4	0.99	0.62	0.26/0.22
+4G_S5, D168N, Y192V, K200C	D168X, Y192X, D194X, K200X	0.31	Target		55 ± 5	~1	0.50	0.35/0.72
			+4G	A	81 ± 33	~1	0.34	0.02/5E-3
				C	571 ± 188	~1	0.05	0.59/0.27
				T	228 ± 19	~1	0.12	0.03/2E-5
+4G_S6, D168N, Y192C, D194G, K200V	D168X, Y192X, D194X, K200X	0.28	Target		108 ± 14	0.99	0.37	0.49/0.72
			+4G	A	127 ± 8	0.88	0.31	0.02/2E-3
				C	337 ± 58	0.25	0.12	0.48/0.28
				T	192 ± 55	0.27	0.21	0.03/3E-5
Untested +4G variants, same libraries as +4G_S1-S6	168, 192, 194, 200	wt DYDK<br>3of11 AAWV<br>2of11 MYDR<br>2of11 NCGV	1of11 SGGY<br>1of11 SVGV<br>1of11 GGDR<br>1of11 NVDC					
+4T_P1, K200R, also increases activity on +3G	NA	NA	Target		17 ± 5	0.95	0.27	8E-3/8E-6
			+4T	A	118 ± 4	~1	0.04	9E-3/8E-4
				C	7 ± 1	0.98	0.64	0.69/0.56
				G	115 ± 9	~1	0.04	0.29/0.44
+4T_S1, D168A, Y192T, D194A, K200F	D168X, Y192X, D194X, K200X	0.34	Target		161 ± 16	0.97	0.58	0.65/0.54
			+4T	A	401 ± 63	0.96	0.23	2E-3/1E-3
				C	1016 ± 122	0.34	0.09	0.26/0.15
				G	903 ± 403	0.67	0.10	0.09/0.31
+4T_S2, D168A, Y192S, K200F	D168X, Y192X, D194X, K200X	0.34	Target		100 ± 19	~1	0.40	0.67/0.52
			+4T	A	173 ± 48	~1	0.23	3E-3/2E-3
				C	478 ± 67	0.80	0.08	0.25/0.14
				G	135 ± 27	~1	0.29	0.09/0.34
+4T_S3, D168A, Y192H, K200L	D168X, Y192X, D194X, K200X	0.34	Target		87 ± 11	0.98	0.65	0.57/0.43
			+4T	A	421 ± 326	0.97	0.13	3E-3/1E-3
				C	485 ± 135	0.37	0.12	0.29/0.19
				G	525 ± 111	0.72	0.11	0.14/0.38
+4T_S4, D168G, Y192W, D194A, K200Y	D168X, Y192X, D194X, K200X	0.34	Target		51 ± 6	~1	0.54	0.25/0.56
			+4T	A	193 ± 58	~1	0.14	6E-3/7E-5
				C	480 ± 76	0.71	0.06	0.41/0.29
				G	109 ± 19	~1	0.26	0.34/0.15
+4T_S5, D168A, Y192V, D194L, K200L	D168X, Y192X, D194X, K200X	0.96	Target		83 ± 6	0.97	0.56	0.61/0.40
			+4T	A	229 ± 19	0.96	0.20	3E-3/2E-3
				C	728 ± 272	0.16	0.06	0.28/0.18
				G	266 ± 74	0.77	0.18	0.12/0.42

+4T_S6, D168A	C150X, D168X, Y192X, D194X, K200X	0.50	Target		30 ± 2	0.99	0.41	0.43/0.42
			+4T	A	97 ± 10	~1	0.13	5E-3/2E-3
				C	57 ± 8	0.94	0.22	0.51/0.40
				G	49 ± 5	0.96	0.25	0.06/0.18
Untested +4T variants, same libraries as +4T_S1-S6	168, 192, 194, 200	wt DYDK<br>3of15 AYDK<br>2of15 ACLC<br>2of15 AVLL	2of15 AHDL<br>1of15 ASDF<br>1of15 ATAF<br>1of15 AVAF	1of15 GWAY<br>1of15 CCLC<br>1of15 AGDL<br>3of6 KADH	3of6 KLDH			
+5A_C1, S152G, S166A, D168Q	S152X, Y154X, S166X, D168Q, K202X	0.11	Target		668 ± 320	~1	0.15	2E-16/1E-11
			+5A	C	943 ± 744	0.57	0.10	0.34/0.04
				G	165 ± 20	~1	0.60	0.50/0.94
				T	644 ± 191	0.93	0.15	0.16/0.02
+5A_C2, S166A, D168S, T189P, K202Q	S166X, D168X, T189X, T204X, K202Q	0.38	Target		249 ± 64	0.94	0.25	1E-16/5E-12
			+5A	C	229 ± 52	0.92	0.27	0.63/0.35
				G	>375	-	0.17	0.12/0.31
				T	203 ± 52	0.91	0.31	0.26/0.35
+5A_S1, D168Q	S152X, D168X, Y192X, K202X	0.47	Target		85 ± 8	~1	0.19	7E-17/4E-11
			+5A	C	361 ± 60	0.71	0.05	0.31/0.05
				G	24 ± 1	0.98	0.67	0.60/0.94
				T	179 ± 42	~1	0.09	0.09/0.02
+5A_S2, S152C, D168E, N226S	S152X, D168X, Y192X, K202X	0.47	Target		539 ± 93	0.61	0.04	1E-16/6E-12
			+5A	C	26 ± 4	0.95	0.77	0.44/0.24
				G	115 ± 15	~1	0.17	0.48/0.75
				T	1056 ± 318	0.55	0.02	0.08/7E-3
+5A_S3, S152I, D168E	S152X, D168X, Y192X, K202X	0.27	Target		597 ± 243	0.89	0.02	5E-17/1E-10
			+5A	C	19 ± 1	0.98	0.58	0.31/0.05
				G	28 ± 3	0.97	0.39	0.69/0.95
				T	1008 ± 281	0.75	0.01	2E-4/1E-6
+5A_S4, D168E	S152X, D168X, Y192X, K202X	0.47	Target		674 ± 335	~1	0.02	5E-16/3E-11
			+5A	C	20 ± 2	0.97	0.61	0.41/0.17
				G	35 ± 6	0.95	0.35	0.47/0.80
				T	611 ± 115	0.81	0.02	0.13/0.03
Untested +5A variants, same libraries as all +5A variants	152, 166, 168, 189, 202, 204	wt SSDTKT<br>3of11 TAQTKT<br>2of11 SAQTKT<br>2of11 GAQTKT	2of11 CAQTKT<br>1of11 TSQTKT<br>1of11 GHQTKT<br>3of5 SASPQA	1of5 SASPQT<br>1of5 SAAPQT<br>8of14 ISETKT<br>2of14 SSQTKT	1of14 CSETKT<br>1of14 VSETKT<br>1of14 SSETKT<br>1of14 LSETKT			
+5C_C1, S152T, D168E, Y192R	S152X, S166X, D168X, Y192R/K (2 libraries)	0.51	Target		116 ± 18	0.97	0.48	0.39/0.18
			+5C	A	436 ± 325	0.41	0.13	2E-3/2E-14
				G	164 ± 48	0.90	0.34	0.61/0.82
				T	1042 ± 798	~1	0.05	1E-4/5E-4
Untested +5C variants, same library as +5C_C1	152, 166, 168, 192	wt SSDY<br>15of16 PSEY<br>1of16 SSEY<br>7of20 CVDK	2of20 ISER<br>2of20 TSER<br>2of20 TAER<br>1of20 VCDK	1of20 CCDK<br>1of20 GYDK<br>1of20 CAEK<br>1of20 SSER	1of20 GFDK<br>1of20 SSEY			
+5G_C1, S152C, S166K, Y192H, K202S	S152X, S166K, D168X, Y192X, K202X	>1	Target		232 ± 43	0.98	0.39	0.91/0.21
			+5G	A	451 ± 168	0.96	0.20	5E-10/1E-10
				C	362 ± 89	~1	0.25	0.09/0.79
				T	569 ± 204	0.98	0.16	2E-3/4E-3
+5G_S1, S152L	S152X, D168X, Y192X, K202X	0.39	Target		11 ± 0.5	0.99	0.55	0.89/0.93
			+5G	A	25 ± 1	~1	0.24	2E-16/6E-12
				C	59 ± 6	~1	0.10	0.08/0.08
				T	60 ± 6	~1	0.10	1E-12/6E-15
+5G_S2, S152C, D168Q	S152X, D168X, Y192X, K202X	>1	Target		39 ± 4	0.98	0.64	0.48/0.95
			+5G	A	150 ± 12	0.84	0.17	2E-16/9E-11
				C	387 ± 54	0.40	0.06	0.45/0.05
				T	182 ± 11	0.79	0.14	0.08/6E-3
+5G_S3, S152T, D168N	S152X, D168X, Y192X, K202X	>1	Target		28 ± 2	0.97	0.67	0.55/0.78
			+5G	A	157 ± 12	0.97	0.12	5E-19/1E-11
				C	207 ± 37	0.44	0.09	0.45/0.22
				T	151 ± 17	0.93	0.12	9E-5/2E-2
+5G_S4, S152T, D168Q	S152X, D168X, Y192X, K202X	>1	Target		35	0.89	0.77	0.62/0.92
			+5G	A	247	0.37	0.11	3E-16/7E-11
				C	548	0.98	0.05	0.39/0.08
				T	372	0.87	0.07	1E-4/1E-3

Untested +5G variants, same libraries as all +5G variants	152, 166, 168, 192, 202				wt SSDYK<br>2of12 CKQEG<br>1of12 TKDWS<br>1of12 CKDGA	1of12 CKNAD<br>1of12 CKDVA<br>1of12 CKDGA<br>1of12 CKDAA	1of12 CKDHS<br>1of12 TKDCQ<br>1of12 CKDQA<br>1of12 SKDCA	4of11 LSDYK<br>2of11 TSNYK<br>2of11 TSEYK<br>1of11 TSQYK<br>1of11 CSQYK<br>1of11 ASEYK
+6C_C1, Y154W, S166G, Y192R, K202Q	Y154X, S166X, T189X, Y192R, K202X	>1	Target		59 ± 4	~1	0.76	0.88/1E-3
			+6C	A	403 ± ?	0.39	0.11	0.02/5E-3
				G	>691	-	0.07	0.11/0.99
				T	691 ± 180	0.64	0.07	2E-6/2E-45
+6C_S1, S152C, Y154W, S166A, L234Q	S152X, Y154X, S166X, K202X	0.60	Target		303 ± 64	0.91	0.64	9E-3/1E-3
			+6C	A	>1585	-	0.12	4E-3/0.02
				G	>1585	-	0.12	0.99/0.98
				T	1585	0.43	0.12	5E-12/8E-12
Untested +6C variants, same libraries as all +6C variants	152, 154, 166, 192, 202				wt SYSTYK<br>3of17 SWGPRL<br>3of17 SWGARQ<br>2of17 SWGQRQ	2of17 SYAPRL<br>1of17 SYRTYG<br>1of17 SYCRRQ<br>1of17 SYAPRQ	1of17 SWAPRK<br>1of17 SYARRQ<br>1of17 SYAPRA<br>1of17 SWAPRQ	4of7 CWATYK<br>2of7 CWSTYK<br>1of7 SWGTYK
+6T_S1, S152A, Y154A, S166I, T189P, K202H	S152X, Y154X, S166X, T189X, K202X	<0.001	Target		437 ± 247	~1	0.43	0.25/0.23
			+6T	A	989 ± 944	0.47	0.19	0.10/0.02
				C	>989	-	0.19	0.27/0.68
				G	968 ± 751	0.62	0.19	0.38/0.07
+7A_P1, mutations + extended loop 153-164	NA	NA	Target		8 ± 2	1	0.47	2E-5/0.10
			+7A	C	74 ± 4	0.97	0.05	0.35/0.52
				G	328 ± 23	~1	0.01	0.08/0.05
				T	8 ± 1	0.98	0.47	0.57/0.33
+7A_P2, mutations + shorter loop 155-163	NA	NA	Target		20 ± 7	0.93	0.76	2E-5/0.10
			+7A	C	588 ± 151	~1	0.03	0.31/0.58
				G	459 ± 190	0.75	0.03	0.07/0.07
				T	86 ± 30	0.94	0.18	0.62/0.24
+7C_S1, T189V, T204R	Y154X, L156X, T189X, T204X	0.30	Target		242 ± 57	~1	0.75	0.31/0.75
			+7C	A	2111 ± ?	0.47	0.09	4E-5/0.14
				G	>2111	-	0.09	0.06/0.09
				T	>2111	-	0.09	0.63/0.02
Untested +7C variants, same library as +7C_S1	189, 204<br>wt TT<br>4of11 PR<br>4of11 VR<br>3of11 TR							
Untested +7G variants, several motif-based libraries	154X/R, 156X, 157X/wt, 166X/wt, 164X/R, 202X/wt, 204X	0.51	154, 156, 157, 164, 166, 202, 204		wt YLNISKT<br>2of6 RKAISKA<br>1of6 YWRRSKE<br>1of6 RKTISKA	1of6 YRRRSKE<br>1of6 YRRISKE<br>7of7 RLNRSQA<br>5of8 GVNRREG	1of8 RVNRSQT<br>1of8 RLNRSQA<br>1of8 SVNRRQG	
Untested +8A variants	L156X, N157X, I164X, T189X, T204X	0.53	156, 157, 164, 189, 204		wt LNITT<br>4of16 QPSTS<br>1of16 HAATS<br>1of16 QPSTS	1of16 GRITG<br>1of16 VRLTQ<br>1of16 PVATA<br>1of16 PGTTN	1of16 LATPN<br>1of16 LAATC<br>1of16 SATGA<br>1of16 GAVTV	1of16 VALAA<br>1of16 GAATT
+8C_P1, L156Q, I164R, T204S	NA, $EC_{1/2max}$ collected for +8C only = 2.9 nM, Thyme *et al*, 2009	NA	Target		1.2E-1 ± 4E-3	NA	0.78	0.92/0.87
			+8C	A	7E-3 ± 1E-3	NA	0.05	1E-4/5E-3
				G	1.8E-2 ± 9E-3	NA	0.12	0.01/0.04
				T	9E-3 ± 7E-4	NA	0.06	0.07/0.09
Untested +8C variants, used as a control for selection method	L156X, I164X, T204X	0.31	156, 164, 204		wt LIT<br>5of27 CRS<br>4of27 PAG<br>4of27 QRS	3of27 PSA<br>2of27 TRS<br>2of27 VSN<br>1of27 HCH	1of27 PCH<br>1of27 HAG<br>1of27 NAN<br>1of27 NVL	1of27 SAC<br>1of27 NVS
Untested +8C variants	L156X, N157X, I164X, T189X, T204X	0.75	156, 157, 164, 189, 204		wt LNITT<br>3of15 SGATG<br>1of15 AVRTD<br>1of15 PAAVG	1of15 VGSTH<br>1of15 VCATN<br>1of15 SLSTH<br>1of15 ESSSA	1of15 SVHGS<br>1of15 PRNTH<br>1of15 PIRTG<br>1of15 SSASV	1of15 ARGTT<br>1of15 WGSSS
Untested +8G variants	L156X, N157X, I164X, T189X, T204X	0.69	156, 157, 164, 189, 204		wt LNITT<br>1of7 HCAPD<br>1of7 LQTTE<br>1of7 WRRTE	1of7 GNSPC<br>1of7 LRHTE<br>1of7 SRCRD<br>1of7 RAATQ		

Table S2. Data and notes on all the multiple base-pair I-AniI variants described in this paper. The related graphs of cleavage data is in Figure S8. The data includes k_{cat}/K_M, % cleavage, and $EC_{1/2max}$. Sequences of variants that were not selected are also included, as well as starting libraries and corresponding survivals.

Selected Variant Name and Tested Sequence	Starting Library Examples, X = all 20 AAs (either *NNN* or *NNS*), and other codons use the accepted code for degerate base-pairs to define the amino acids included in the library. Example results from these different libraries are shown.	Library Survival (0 - 1), round 2 or round 3	Target Site		$EC_{1/2max}$ (nM), % cleavage, or k_{cat}/K_M for some enzymes	Cleavage Plateau (f_{max}), NA for % cleavage or k_{cat}/K_M
CPK2_N<br>Y18C, T22S, G33K, S57T, R59T, A68H, R70E, enzyme expressed extremely poorly, so values in columns for activity data are percent cleavage with two concentrations, the highest possible and then 1/4 of that - corresponding to approximately 1,700 nM for the lower value.	1) Y18C, S20X, T22S, E31R, G33*MAG*, R59T, M66X, A68X, R70E<br>2) Y18C, T22S, E31R, G33MAG, S57X, R59X, A68X, R70E<br>3) Y18C, T22S, E31R, G33K, S57WCC, R59ASS, M66T, A68SWC, R70E<br><br>left: 18, 20, 22, 31, 33, 57, 59, 66, 68, 70 from libraries 1 and 2 mixed<br>right: 18, 22, 31, 33, 57, 59, 66, 68, 70 from library 3<br><br>2of10 CLSRKSTTLE<br>2of10 CSSRKSTNDE<br>1of10 CSSRKETMLE<br>1of10 CSSRRSTTVE<br>1of10 CSSRKSMMVE<br>1of10 CSSRKARMLE<br>1of10 CSSRQTRMLE<br>1of10 CSSRQGSMAE<br><br>4of7 CSRKTTTHE<br>1of7 CSRKSSTVE<br>1of7 CSRKTRTVE<br>1of7 CSRKTSTVE	0.11 for R2 of libraries 1 and 2 mixed	Target		0.79, 0.36	NA
			-6C	A	0, 0	NA
				G	0, 0	NA
				T	0, 0	NA
			Target		0.79, 0.36	NA
			-5A	C	0, 0	NA
				G	0.15, 0	NA
				T	NA	NA
			Target		0.79, 0.36	NA
			-4T	A	0.66, 0	NA
				C	0.82, 0.42	NA
				G	0.77, 0	NA
			Target		0.79, 0.36	NA
			-2G	A	0.74, 0.30	NA
				C	0.28, 0	NA
				T	0.78, 0.23	NA
			Target		0.79, 0.36	NA
			+1A	C	0.65, 0	NA
				G	0.76, 0.17	NA
				T	0.72, 0.23	NA
CPK2_Cgg, +4G and +5G pocket, untested	1) S152*WBS*, S166*ARS*, D168*VVS*, 192X, K200X, K202X<br>2) S152C, S166K, D168*GSC*, 192X, 194X, K200X, K202*BMJ*<br>3) S152*WBS*, S166X, D168K, 192X, K200H, K202X<br><br>152, 166, 168, 192, 200, 202<br>1of6 CKAERS<br>1of6 TKAGRD<br>1of6 SKAVRD<br><br>1of6 CKAERS<br>1of6 CKGVSQ<br>1of6 CKAERS	0.29 for R2 of all three libraries mixed	NA			
CPK2_C1, S152C, K155R, L156K, Y162H, I164V, S166K, D168A, T189S, Y192G, K200R, K202D	The following three libraries are some of the first based on the Cgg pocket library results and expanding to try and target the full C half-<br>1) S152C, L156X, I164X, S166K, D168A, Y192*GDG*, K200R, K202*BWS*, T204X<br>2) S152C, I164X, S166K, D168A, T189X, Y192*GDG*, K200R, K202*BWS*, T204X<br>3) S152C, K155X, L156X, I164X, S166K, D168A, Y192*GDG*, K200R, K202*BWS*, T204X<br>152, 155, 156, 164, 166, 168, 189, 192, 200, 202, 204<br>3of24 CKLRKAFGRLV<br>2of24 CKLWKATGRLI<br>1of24 CKLRKAGGRLA<br>1of24 CKLWKAPGRFM<br>1of24 CKALKATGRHT<br>1of24 CKLGKAAGRQL<br>1of24 CKLVKADGRDI<br>1of24 CKLLKAGGRDL<br>1of24 CKLGKDDERYA<br>1of24 CKLFKAGERLI<br>1of24 CKLYKAGGRQE<br><br>1of24 CKLYKAGGRVM<br>1of24 CKLEKARVRDM<br>1of24 CTALKATGRHY<br>1of24 CFSAKATGRYL<br>1of24 CSGLKATVRVV<br>1of24 CGMCKATGRLY<br>1of24 CHLCKATERQV<br>1of24 CETPKATVREA<br>1of24 CFSAKATGRYL<br>1of24 CREIKATVRLI<br>4) S152C, K155X, L156*RNG*, I164*BNS*, S166K, D168A, T189*BNC*, Y192*GRG*, K200R, K202*BWS*, T204*DWS*<br>5) Same as 4, but with Y162X added<br>152, 155, 156, 162, 164, 166, 168, 189, 192, 200, 202, 204<br>4of15 CTEHWKACERFL<br>3of15 CEKYQKAAGRLN<br>2of15 CQTYPKACERFI<br>2of15 CYRYVKAYEREE<br><br>1of15 CPVYHKASERLD<br>1of15 CTTYVKASGRLV<br>1of15 CFTYPKADERET<br>1of15 CPGYCKALERVV	Survivals for these initial libraries (1-5) was lower than 0.01	Target		44	0.54
			+1A	C	31	0.49
				G	NA	NA
				T	135	0.20
			Target		44	0.54
			+4G	A	>750	NA
				C	>750	NA
				T	50	0.61
			Target		44	0.54
			+5G	A	>750	NA
				C	>750	NA
				T	>750	NA
			Target		44	0.54
			+7G	A	32	0.73
				C	15	0.86
				T	10	0.83
			Target		44	0.54
			+9C	A	62	0.45
				G	32	0.75
				T	49	0.56
			Target		44	0.54
			+10C	A	NA	NA
				G	511	0.42
				T	207	0.45

CPK2_C2, S152C, K155R, L156K, Y162H, I164V, S166K, D168A, T189S, Y192G, K200R, K202D, T204A

6) S152C, K155R, L156X, Y162H, I164X, S166K, D168A, T189S, Y192G, K200R, K202E, T204X
7) S152C, K155R, L156K, Y162H, I164V, S166X, D168X, T189S, Y192G, K200R, K202X, T204A
8) S152X, K155R, L156K, Y162H, I164V, S166K, D168A, T189S, Y192G, K200R, K202X, T204X
Library 6 results, Library 7 results, Library 8 results (positions above)

Library 6

2of8 CRDHPKASGRES
2of8 CRSHRKASGREK
1of8 CRDHPKASGRET
1of8 CRAHVKASGRET
1of8 CRVHNKASGREG
1of8 CRNHEKASGRED

Library 7

10of16 CRKHVKSSGRDA
4of16 CRKHVKGSGRDA
1of16 CRKHVKASGRDA
1of16 CRKHVKMSGRDA

Library 8

12of14 CRKHVKASGRDA
1of14 CRKHVKASGRDV
1of14 CRKHVKASGRDT

Libraries 6-8 showed the extreme importance of K202D, D168S, and T204A for high survival

Library 6 had survival of 0.03, library 7 had a survival of 0.47, and library 8 had a survival of 0.36

Target			14	0.91
+1A	C		13	0.88
	G		NA	NA
	T		18	0.84
Target			14	0.91
+4G	A		45	0.65
	C		124	0.20
	T		14	0.94
Target			14	0.91
+5G	A		31	0.78
	C		120	0.43
	T		227	0.88
Target			14	0.91
+7G	A		13	0.89
	C		12	0.90
	T		10	0.88
Target			14	0.91
+9C	A		21	0.86
	G		49	0.82
	T		20	0.84
Target			14	0.91
+10C	A		NA	NA
	G		61	0.91
	T		38	0.86

CPK2_C3, S152C, K155R, L156K, Y162H, I164V, S166K, D168S, T189S, Y192G, K200R, K202D, T204A

9) S152C, K155R, L156K, N157X, D158X, D160X, D161X, Y162H, I164V, S166K, D168S, T189S, Y192G, K200R, K202D, T204A
10) S152C, K155R, L156K,Y162H, I164V, S166K, D168S, A170X, T189S, Y192G, D194X, T196X, C198X, K200R, K202D, T204A
11) S152C, K155R, L156K,Y162H, I164V, S166K, D168S, T189S, Y192X, D194X, K200X, K202D, T204A
Library 9 results, Library 10 results, Library 11 result (positions above)

Library 9

1of14 CRKSSETEHVKSSGRDA
1of14 CRKSPESRHVKSSGRDA
1of14 CRKSPEWAHVKSSGRDA
1of14 CRKCPETGHVKSSGRDA
1of14 CRKSIENGHVKSSGRDA
1of14 CRKAIESEHVKSSGRDA
1of14 CRKALESRHVKSSGRDA
1of14 CRKAVESRHVKSSGRDA
1of14 CRKAPESCHVKSSGRDA
1of14 CRKSLESSHVKSSGRDA
1of14 CRKSRESSHVKSSGRDA
1of14 CRKSVEVSHVKSSGRDA
1of14 CRKALESHHVKSSGRDA
1of14 CRKALESHHVKSSGRDA

Library 10

3of7 CRKHVKSGSGEPFRDA
1of7 CRKHVKSASGEPLRDA
1of7 CRKHVKSASGSDLRDA
1of7 CRKHVKSGSGDNFRDA
1of7 CRKHVKSGSGEGFRDA

Library 11

7of24 CRKHVKSSTDRDA
5of24 CRKHVKSSSDRDA
3of24 CRKHVKSSYDRDA
3of24 CRKHVKSSHDRDA
2of24 CRKHVKSSCDRDA
1of24 CRKHVKSSVDRDA
1of24 CRKHVKSSYMRDA
1of24 CRKHVKSSQDRDA
1of24 CRKHVKSSYCRDA

Libraries 9-11 showed the importance of keeping K200R and D194D (Library 11), that A170 should be A or G and C198 should be hydrophobic (Library 10), and that there is a preferred sequence for loop 157-161 (Library 9)

The tested proteins C_1 to C_3 were from libraries 6-8.

Library 9 had a survival of 0.67, and library 10 had a survival of 0.29

Target			9	0.94
+1A	C		8	0.90
	G		NA	NA
	T		9	0.87
Target			9	0.94
+4G	A		7	0.84
	C		29	0.75
	T		9	0.96
Target			9	0.94
+5G	A		11	0.89
	C		15	0.83
	T		10	0.93
Target			9	0.94
+7G	A		8	0.91
	C		8	0.92
	T		6	0.88
Target			9	094
+9C	A		9	0.91
	G		11	0.91
	T		8	0.86
Target			9	0.94
+10C	A		NA	NA
	G		33	0.88
	T		20	0.84

FAH1313_Ccc, +5C and +6C pocket, S152T, S166G, T189P, Y192K, K202L

S152*DYC*, Y154*TRS*, S166*GBC*, D168*GAS*, T189*MCC*, T192*MRG*, K200*HWG*

152, 166, 189, 192, 200 (154 and 168 stayed wild-type, Y and D)

4of9 TGPRL
2of9 TAPKL
1of9 AGPRL

1of9 SGPRL
1of9 TSPKL

2of4 TGPKL
2of4 TGPRL

Survival for one selection was 0.33, for the other it was 0.48. They were both from the same library.

Target			117	0.80
+3C	A		NA	NA
	G		85	0.95
	T		15	0.84
Target			117	0.80
+5C	A		>750	NA
	G		>750	NA
	T		>750	NA
Target			117	0.80
+6C	A		>750	NA
	G		>750	NA
	T		>750	NA
Target			117	0.80
+9G	A		27	0.87
	C		234	0.29
	T		110	0.79

+3C_S1 with +1/+3/+5 substrates	Substrates are labeled 1-4. 1=+3C, 2=+1G/+3C, 3=+3C/+5G, and 4=+1G/+3C/+5G This data is associated with Figure 5. The kinetics section is shown as k_{cat}/K_M values to show the individual components of the measure.		1	178	~1
			2	65	~1
			3	197	0.56
			4	312	~1
+3C_S2 with +1/+3/+5 substrates			1	324	~1
			2	75	~1
			3	432	0.36
			4	585	0.39
+3C_S1 kinetics with +1/+3/+5 substrates			1	1.1/294	NA
			2	3.6/95	NA
			3	0.10/72	NA
			4	0.18/256	NA
Y2 kinetics with +1/+3/+5 substrates			1	0.11/33	NA
			2	2.0/71	NA
			3	0.17/6	NA
			4	0.14/84	NA
SV	-4C C1: Y18C, G33K, R59T, A68R	-4T		97	~1
SV2	-4C C2: Y18C, G33K, R59S	-4T		226/58	~1/~1
SV	-4C C1: Y18C, G33K, R59T, A68R	AGAP004671 (-)		270/199	~1/~1
SV2	-4C C2: Y18C, G33K, R59S	AGAP004671 (-)		1392/1866	~1/~1
Untested AGAP004671 (-) half-site variants	These loop libraries were built in the context of SV2, which we know now is a very poor cutter of the (-) half-site. These sequences should be retested in the context of the alternative variant (-4C_C1). The survival was <1% against the entire (-) half-site. These loops were additionally tested in the context of E63E/I64N/E65R from a loop library over the -2 position, but the length and sequence of this loop should also be further randomized.	wt_23to27 - KKGKY<br>5of14 MQRSKA<br>3of14 GQRGKA<br>2of14 SQQAKA<br>2of14 DKDRKA<br>2of14 YHDRKA			
HV	S152V, D168S, T189V, K200H, T204R	AGAP004671 (+)		50/43	0.96/0.94
Human Cells HV	L156D, D168S, T189V, K200H, T204R	AGAP004671 (+)		NA	NA
Notes on AGAP004671 (+)	The sequence used in enzyme assays (Figure 6) was selected for the highly similar AGAP003225 (+) half-site. The sequence specific to AGAP004671 (+) is the version that was tested in human cells (Figure S9, and see above row). Selection survival is 72%.				
SV	+3G S1: A170K, Y192G, K200V	+3G		240/420	0.83/0.73
SV	+3G S1: A170K, Y192G, K200V	AGAP009607 (+)		197/175	0.82/0.82
SV	-3A S1: Y13W, E35A, 58-65 = LRQHDRG-	-3A		177	~1
SV	+3C S1: A170E, D194K, K200A	+3C		145/153	~1/~1
SV	+3C S1: A170E, D194K, K200A	AGAP010417 (+)		318/298	0.82/0.77
SV	-3C C2: Y18W, E35K, 58-65 = FRPDG---	-3C		6.6/4.6	0.98/~1
SV	-3C C2: Y18W, E35K, 58-65 = FRPDG---	AGAP003225 (-)		7.4/7.3	0.98/1
HV	S152V, D168S, T189V, K200H, T204R	AGAP003225 (+)		24/20	0.91/0.91
Human Cells HV	S152V, D168S, T189V, K200H, T204R	AGAP003225 (+)		NA	NA
AGAP003225 FV	Y18W, E35K, 58-65 = TRPDG---, S152T, D168S, T189V, K200H, T204R, and base mutations to provide stability/activity, but not specificity: F13Y, K49D, I53V, I55V, S79N, I81L, E86D, F91I, S92T, R102K, S111Y, L112S, L232K	$EC_{1/2max}$		121/141	0.92/0.93
CPK2 FV	Y18C, T22S, E31R, G33K, R59T, A68H, R70E, S152C, 155-168: RKALESSHLVAKFS, T189S, Y192S, D194I, T196R, C198I, K200R, K202D, T204A, and same base mutations as AGAP003225	$EC_{1/2max}$		1185/1204	~1/~1
		K_M		>3,000	NA
		k_{cat}		>5	NA
notes on AGAP009607 (-)	Need to determine whether -4C_C1 and/or -4C_C2 can cleave -5G/-4C. Then the mutations for -8G_P1 (K24N, T29K) need to be combined with -4C mutations. This variant should cleave all (-) half substitutions except -10C, requiring loop remodeling.				
notes on AGAP009607 (+)	A sequence similar to the tested single variant +3G_S1 showed survival of 32%. This sequence was A170K, D194A, C198A, K200C.				
notes on AGAP010417	Suprisingly, the +3C_S1 variant cleaves the (+) half-site more poorly than the +3C base alone. This is surprising because we also showed that +3C_S1 cleaves +1G/+3C better than +3C (Figure 5). Therefore, good cleavage of this half-site likely needs randomization over the third substition, +9C.				
notes on AGAP008878 (-)	Need to dermine whether -3T_S1 can cleave -3T/-2C. Then the mutations for -8T_P1 (K24N, T29Q) need to be combined with the -3T S1 sequence. This variant should cleave all (-) half substitutions except for -10G, requiring loop remodeling.				
notes on AGAP008878 (+)	This site includes the +4G/+5G substitutions in the CPK2 (+) half-site. It is cleaved with 50-100% survival by variations of the enzyme for this pocket. The variant showing up the most often in libraries based on the CPK2 information is: S152C, L156R, I164F, S166K, D168G, Y192V, K200R, K202D				
notes on AGAP002800 (-)	Combining -8G_P1 and -3C_C2 will almost certainly yield a highly active variant for this entire half-site, as -5T is cleaved as well as the wild-type nucleotide at position -5.				
notes on AGAP002800 (+)	Need to determine whether any of the variants the cleave +4T can tolerate +5C. Additionally, the +1C central four substitutions may result in unexpected specificity preferences.				
Y192I with +6 substrates	This data is associated with Figures S10 and S11 and Supplementary Discussion B. The kinetics section is shown as kcat/KM values to show the individual components of the measure. The Y2 kinetic data is from Thyme, et al, Nature 2009.	Target		0.049/28	NA
		+6A	C	3.13/168	NA
			G	5.59/170	NA
			T	0.11/68	NA
